# TLR1‐Regulated Ferroptosis Gene Decreases the Occurrence of Restless Legs Syndrome

**DOI:** 10.1002/brb3.71038

**Published:** 2025-11-11

**Authors:** Qunshan Chen, Xin Men

**Affiliations:** ^1^ Pain Management Centre, Department of Anesthesiology The Second Affiliated Hospital of Zhejiang University School of Medicine Hangzhou Zhejiang Province China; ^2^ Department of Anesthesiology Hangzhou Women's Hospital (Hangzhou Maternity and Child Health Care Hospital) Hangzhou Zhejiang Province China

**Keywords:** ferroptosis, Mendelian randomization, restless legs syndrome

## Abstract

**Background:**

Restless legs syndrome (RLS) is a sensory motor neuropathy that is frequently encountered. Transcriptome analysis has revealed the role of ferroptosis in the pathogenesis of RLS. However, the role of ferroptosis in RLS and the upstream regulatory molecules governing ferroptosis remain unknown.

**Methods:**

This study aims to explore the causal relationship and mechanisms between ferroptosis genes, upstream genes, and RLS using Mendelian Randomization (MR). This study employed two‐sample MR analysis and mediation analysis, utilizing RLS GWAS data from the Finngen database, ferroptosis genes data from the decode database, and upstream genes data from the the UK Biobank Pharma Proteomics Project. A two‐sample MR analysis was used to evaluate the causal relationship between the ferroptosis genes and RLS. Then, mediation analysis was conducted to explore the mediating effect of upstream genes on the relationship between expression of ferroptosis genes and RLS.

**Results:**

Our study found a significant positive correlation between high expression of the FURIN gene and a reduced risk of RLS. Further exploration of upstream genes regulating ferroptosis genes revealed that these genes indirectly influence the occurrence of RLS by regulating the expression of the FURIN gene. Mediator effect analysis showed that TLR1 indirectly reduces the occurrence of RLS by regulating the expression of the FURIN gene, with the mediator effect of FURIN gene expression accounting for 17.9% of the total effect. Sensitivity analysis supported our findings, indicating high statistical robustness.

**Conclusion:**

This study highlights the role of TLR1 in regulating FURIN during the progression of RLS, suggesting the potential clinical application of FURIN as a therapeutic target for the early diagnosis and treatment of RLS.

## Graphical abstract

This study aims to explore the causal relationship and mechanisms between ferroptosis genes, upstream genes, and RLS. A two‐sample MR analysis was used to evaluate the causal relationship between the ferroptosis genes and RLS. Then, mediation analysis was conducted to explore the mediating effect of upstream genes on the relationship between expression of ferroptosis genes and RLS. The result may be a new therapeutic target for further RLS study and offers novel ideas about the relationship between RLS and ferroptosis.

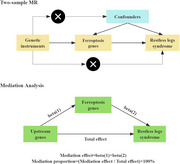

**Abbreviations**
AD: Alzheimer's disease
APP: Amyloid‐β precursor protein
CI: Confidence interval
DA: Dopaminergic
DSS: Dextran sulfate sodium
FURIN: Furin, paired basic amino acid cleaving enzyme
GPR37: G protein‐coupled receptor 37
GPX4: Glutathione peroxidase 4
GSH: Glutathione
GWAS: Genome‐wide association study
IV: Instrumental variable
IVW: Inverse variance weighting
LD: Linkage disequilibrium
MMP‐3: Matrix metalloprotease‐3
MR: Mendelian Randomization
MR–Egger: A method for detecting and adjusting for pleiotropy in Mendelian Randomization
MR–PRESSO: Mendelian Randomization Pleiotropy Residuals and Outlier
OR: Odds ratio
PD: Parkinson's disease
PLMs: Periodic limb movements
PQTL: Protein quantitative trait loci
RLS: Restless legs syndrome
ROS: Reactive oxygen species
SD: Standard deviation
SNP: Single nucleotide polymorphism
TLR1: Toll‐like receptor 1
UKB‐PPP: UK Biobank Pharma Proteomics Project

## 1 Introduction

Restless legs syndrome (RLS) is a sensory motor neuropathy that is frequently encountered. It is distinguished by an inexorable compulsion to move the legs, which is most pronounced during periods of inactivity and at night (Gossard et al. 2021). An involuntary movement of the legs during sleep is frequently associated with RLS. This condition is known as periodic limb movements during sleep (PLMs) (Cederberg et al. 2024). The age of onset for RLS is highly variable, with symptoms having the potential to manifest from childhood to over 80 years of age (Winkelmann et al. 2000). However, it is frequently underdiagnosed in the general population. Because of the criteria employed to define the condition in research, prevalence estimates for RLS are subject to significant variation. It is estimated that the prevalence of clinically significant RLS ranges from 2% to 3% (Allen et al. 2011). RLS is more prevalent in women and as individuals age. Pregnant women are more likely than normal to experience RLS during the third trimester, as the risk increases as the number of live births increases (Manconi et al. 2004). Many patients with RLS experience sleep onset or maintenance insomnia, which can result in decreased quality of life (Earley and Silber 2010), depression, and a higher risk of suicide (Para et al. 2019; Zhuang et al. 2019). The pathogenesis of RLS, however, remains uncertain. There is a growing body of evidence that suggests that the development of RLS is influenced by dopamine neurotransmission abnormalities and iron deficiency in the brain.

Transcriptome analysis has revealed the role of ferroptosis in the pathogenesis of RLS (Mogavero et al. 2024). It has recently been discovered that ferroptosis is an iron‐dependent form of cell death (Jiang et al. 2021). It is initiated by iron‐dependent lipid peroxidation and ultimately results in cell death. Ferroptosis has been shown to have a significant role in the emergence of several neurodegenerative diseases (Yan et al. 2021). Neurodegeneration, reactive oxygen species (ROS), and mitochondrial damage result from ferroptosis. The pathology of Alzheimer's disease (AD) and Parkinson's disease (PD) has been observed to include abnormal iron metabolism and lipid peroxidation (Yan et al. 2021). In AD and PD, iron accumulation has been reported in afflicted brain regions (Belaidi and Bush 2016; Masaldan et al. 2019). Reduced ferritin levels in cerebrospinal fluid are linked to RLS (Khachatryan et al. 2022). Singleton suggested that the way mitophagy, dopamine metabolism, and the immune system work together is key to understanding how Parkinson's disease develops (Singleton and Hardy 2019). Neurodegeneration involves networks like mitophagy, ferroptosis, and p53 signaling pathways (Mogavero et al. 2024). These different neurodegenerative pathways are linked to RLS; they all lead to problems with dopamine that are typical in both RLS and neurodegenerative diseases, which helps to understand why iron supplements work for RLS (Mogavero et al. 2024). However, the role of ferroptosis in RLS and the upstream regulatory genes governing ferroptosis remain unknown.

Initially, we utilized a dual protein quantitative trait loci (pQTL) database to identify genes that regulate ferroptosis and their upstream genes. A two‐sample Mendelian randomization (MR) analysis was then conducted to investigate the causal relationship between these genes and RLS. By reducing the confounding biases that are inherent in observational studies, this method aims to infer causal relationships between genetic variants and complex traits. As the last step, mediation analysis was employed to understand how upstream genes affect ferroptosis genes in RLS, which is divided into direct and indirect effects. It is expected that these results will provide valuable insights into early diagnosis and precise treatment of RLS.

## 2 Methods

### 2.1 Data Source

**FIGURE 1:**
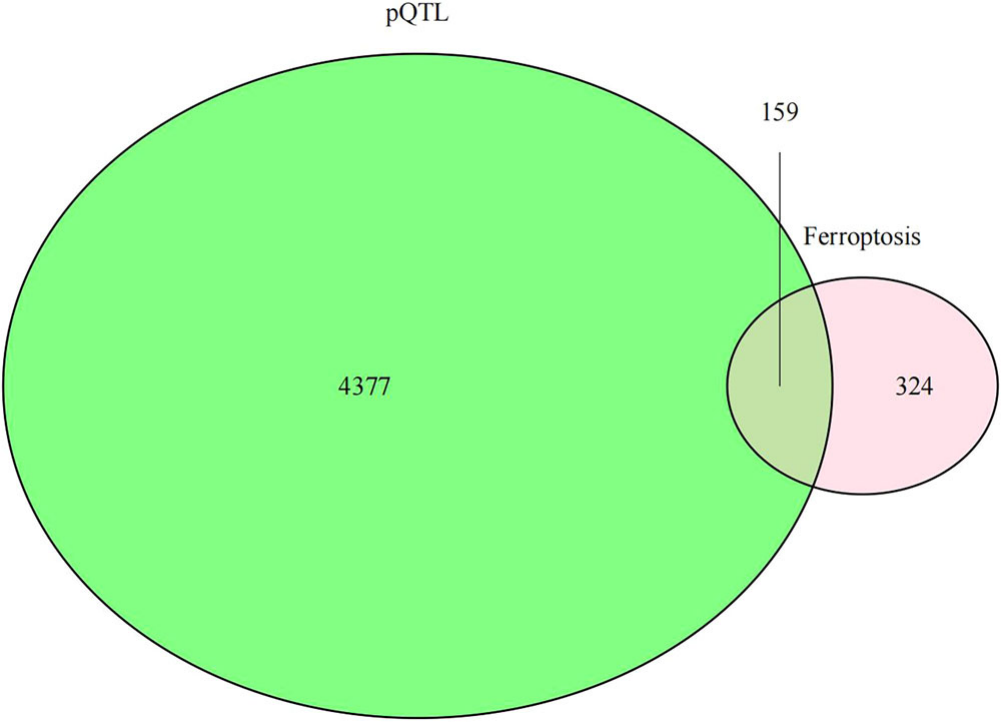
Choose pQTL of ferroptosis genes to investigate the causal relationship between genes and RLS.

We obtained the GWAS data for RLS from the FinnGen database. GWAS ID R12_G6_RLS.gz was assigned to RLS, which includes 4599 RLS patients and 495,749 control samples. The RLS data covered both European males and females and contained almost 500,000 SNPs. 483 ferroptosis genes were obtained from the FerrDb database (http://www.zhounan.org/ferrdb/current/). From the decode database, we acquired data on 159 pQTLs related to ferroptosis genes (https://www.decode.com/summarydata/, Figure 1). Genome‐wide association studies (GWASs) were conducted in the past using 4907 aptamers to measure plasma protein levels in 35,559 Icelanders. In their study, 18,084 sequence variants were identified that were linked to plasma protein levels (protein quantitative trait loci; pQTLs). Next, we investigated genes that were linked to RLS in the UK Biobank Pharma Proteomics Project (UKB‐PPP, https://www.synapse.org/Synapse:syn51364943/wiki/622119). This collaboration meticulously investigated the plasma proteomic characteristics of a cohort of 54,219 UKB participants. Through exhaustive pQTL mapping of 2923 proteins and detailed analysis, we identified a total of 14,287 significant genetic associations (Table S1).

### 2.2 MR Analysis of the Relationship Between Ferroptosis and RLS

**FIGURE 2:**
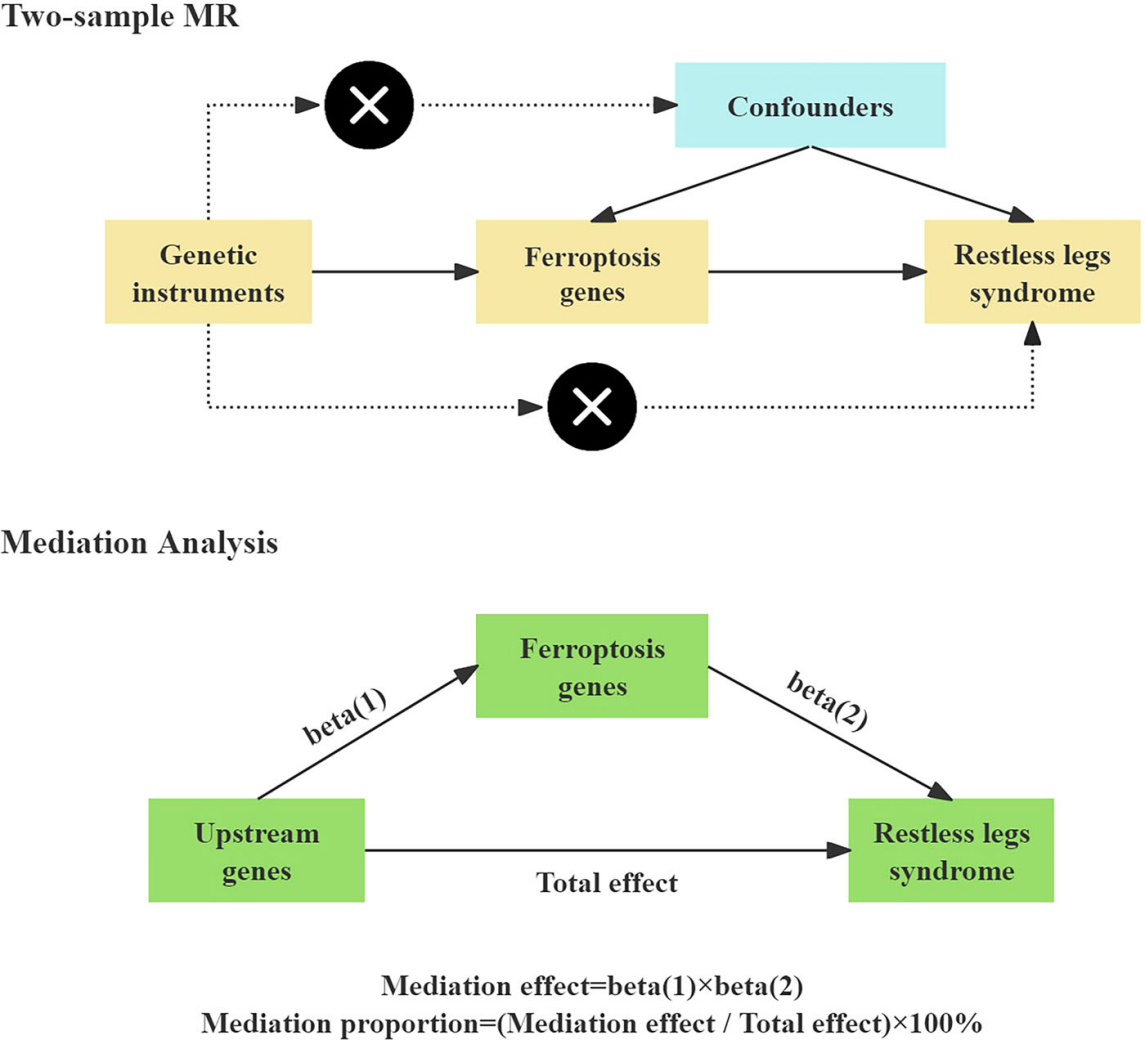
The flowchart of our study design. MR, Mendelian randomization.

The flowchart for this study is shown in Figure 2.

RLS was utilized as the outcome variable while employing the FinnGen database. The causal relationships between the incidence of RLS and the expression of ferroptosis genes were investigated through two‐sample MR analysis. The selection of pertinent SNPs as instrumental variables (IVs) was based on the relevant inclusion criteria. Three foundational assumptions must be met to conduct MR analysis: (1) IVs must be highly correlated with exposure; (2) IVs must not be influenced by any other factors; and (3) horizontal pleiotropy must not occur.

To estimate the potential causal relationship between genetic prediction of ferroptosis genes and RLS, this study used the R software package “TwoSampleMR” (version 0.6.8). To prevent weak instrumental bias, single nucleotide polymorphisms (SNPs) that had a substantial correlation with gene expression (*p* < 5 × 10^−8^) were chosen as instrumental variables (IVs) with an F statistic > 10 (Pierce et al. 2011). In order to establish a set of mutually independent SNPs, the 1000 Genomes Project European population was employed to aggregate the SNPs of each gene with a linkage disequilibrium (LD) threshold of r2< 0.1 and a clustering window of 10,000 kb (Gkatzionis et al. 2023). Using the Wald ratio method for ferroptosis genes containing one SNP was used; with the inverse variance weighting (IVW) method as the primary analysis method, we used MR Egger, weighted median, simple mode, and weighted mode for genes containing two or more SNPs. In the analysis, we calculated the odds ratio (OR) for the increase in RLS risk corresponding to each standard deviation (SD) increase in ferroptosis gene levels.

### 2.3 Sensitivity Analysis

We conducted numerous sensitivity analyses to confirm the study's robustness. The heterogeneity of genetic variants was evaluated using Cochran's Q test, which indicates that there is no significant heterogeneity if the p‐value is larger than 0.05 (Hemani et al. 2018). Horizontal pleiotropy was evaluated using the MR–Egger regression intercept. There is no evidence of horizontal pleiotropy when the p‐value is greater than 0.05 (Hemani et al. 2018). MR Pleiotropy Residual and Outlier (MR–PRESSO) was implemented to identify prospective outliers that may be affected by pleiotropy. For the global test, a p‐value greater than 0.05 indicates the absence of horizontal pleiotropic outliers. A leave‐one‐out analysis was done by repeating the MR analysis after removing one IV at a time to see how each IV affects the overall result.

### 2.4 Mediation Analysis of pQTL Genes—Ferroptosis‐—RLS Relationship

Ferroptosis and upstream genes were investigated for their direct and indirect effects on RLS using the two‐step Mendelian randomization (TSMR) method. According to the TSMR procedure, it is presumed that the mediator and the exposure are not in interaction. Two additional estimates were obtained from the univariate MR analysis in addition to the fundamental effect estimate of the upstream genes on RLS (Beta_all). The two factors included the impact of the mediator (ferroptosis) on RLS (Beta2) and the effect of the exposure (upstream genes) on the mediator (ferroptosis) (Beta1).

## 3 Results

### 3.1 MR Analysis of the Relationship Between Ferroptosis and Restless Legs Syndrome

**FIGURE 3:**
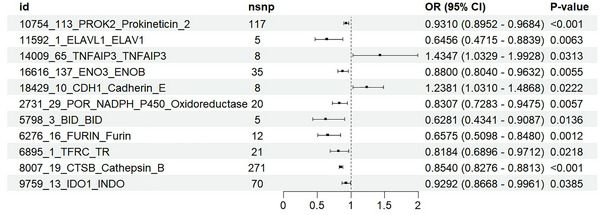
Forest plots showed the causal associations between ferroptosis genes and RLS by the IVW method. IVW: inverse variance weighting; CI: confidence interval.

In this study, the IVW method was used to assess the causal effect of ferroptosis genes on RLS. A total of 159 genes were used as exposure. IVW analysis identified 11 ferroptosis genes significantly associated with RLS (PROK2, ELAVL1, TNFAIP3, ENO3, CDH1, POR_NADPH, BID, FURIN, TFRC, CTSB, IDO1), as shown in Figure 3. Sensitivity analysis showed that only ENO3 and FURIN did not show evidence of heterogeneity or horizontal pleiotropy in Cochran's Q test and MR–Egger regression analysis (Table S3). The MR–PRESSO test did not detect any outliers, and the results of the leave‐one‐out analysis were robust, further supporting the reliability of our MR analysis results (Table S3 and Figures S1 and S2). All selected SNPs had F‐statistics greater than 10, indicating that these SNPs are reliable instrumental variables (IVs) in this study (Table S4). Results from the MR–Egger, weighted median, weighted mode, and simple mode methods are presented in Table S2 and Figures S1 and S2. This figure illustrates the causal influence of ferroptosis genes on RLS risk, as assessed by the IVW method. ENO3 and FURIN are negatively associated with RLS, suggesting a potential protective effect.

### 3.2 Mediation Analysis of Upstream Genes—Ferroptosis‐—RLS Relationship

**FIGURE 4:**
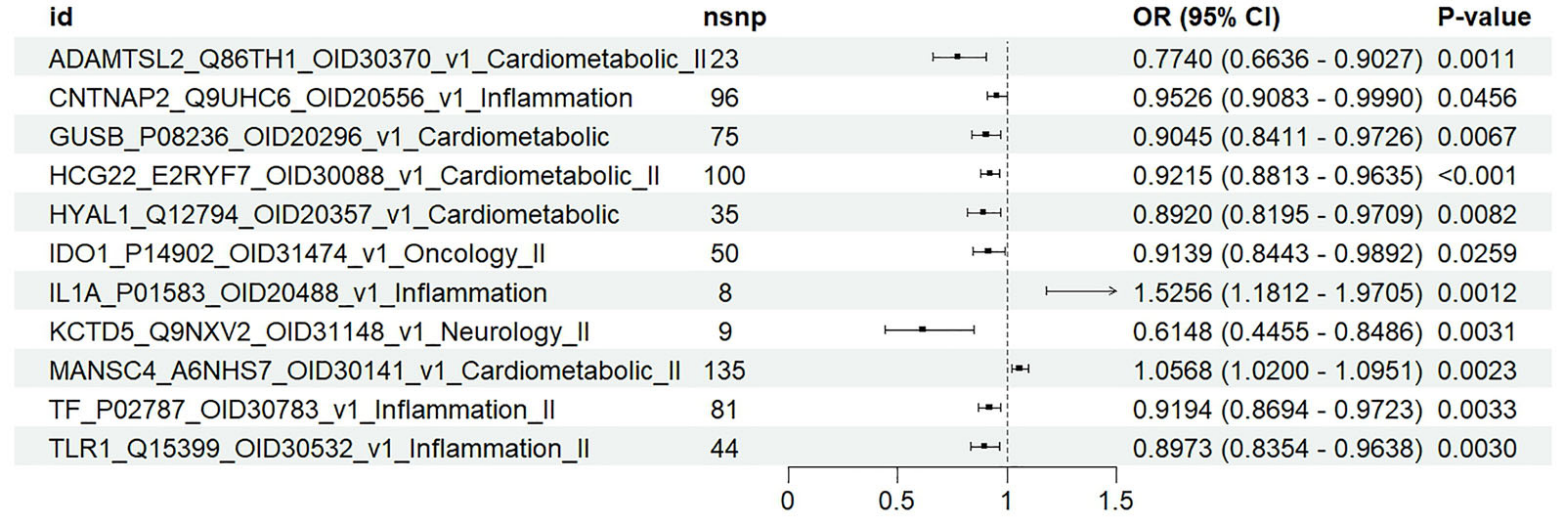
Forest plots showed the causal associations between upstream genes and RLS by the IVW method. IVW: inverse variance weighting; CI: confidence interval.

We obtained the relevant data from the UKB‐PPP database. In the first step, we assessed the effect of upstream genes on RLS expression. The results showed that IL1A, MANSC4, and RLS were positively correlated, while ADAMTSL2, CNTNAP2, GUSB, HCG22, HYAL1, IDO1, KCTD5, TF, and TLR1 were negatively correlated with RLS expression (Figure 4). MR results are presented in Table S5 and Figures S3–S13. IVW analysis showed that all 11 upstream genes significantly influenced RLS expression. All selected SNPs had F‐statistics greater than 10, indicating they are appropriate IVs for MR analysis (Table S7). We also assessed the effect of upstream genes on ferroptosis gene expression. MR results are presented in Table S8 and Figure S14. All selected SNPs had F‐statistics greater than 10, indicating they are appropriate IVs for MR analysis (Table S10). In the second step, we used upstream genes to assess the causal effect of ferroptosis genes as mediators of RLS risk. Combining the results from both steps, we calculated the total effect (Beta all), mediating effect, and direct effect. The proportion of the mediating effect is shown in Table S11. TLR1 indirectly reduces the occurrence of RLS by regulating FURIN gene expression, with FURIN's mediating role accounting for 17.9% of the total effect.

Sensitivity analysis showed no evidence of heterogeneity or level multi‐effectivity in Cochran's Q test and MR–Egger regression analysis (Tables S6 and S9). The MR–PRESSO test did not detect any outliers, and the results of the leave‐one‐out analysis were robust, further supporting the reliability of our MR analysis results (Tables S6 and S9 and Figures S3–S14).

## 4 Discussion

We examined the causal relationship between ferroptosis genes and RLS using two‐sample MR and mediation analysis, identifying a significant association between FURIN expression and RLS risk, which is regulated by TLR1. It is clear from these findings that ferroptosis plays an important role in neuromuscular disorders and in the pathogenesis of RLS. RLS may be treated with FURIN as an early diagnostic and therapeutic target.

Proteolysis is an important post‐translational modification involving the specific hydrolysis of peptide bonds in proteins by proteases (He et al. 2022). Furin is a type I transmembrane protein that belongs to the subtilisin‐like proprotein convertase family (Ivachtchenko et al. 2024; Thomas 2002). It is a calcium‐dependent serine protease (Seidah and Prat 2012). Cysteine is a key component in glutathione (GSH) synthesis and a co‐substrate for glutathione peroxidase 4 (GPX4). According to some studies (Tan et al. 2024), FURIN overexpression has been shown to reduce ferroptosis by increasing GPX4 expression. Through activation of the Nrf2‐Gpx4 signaling pathway, furin could protect epithelial cells against ferroptosis‐like cellular damage induced by Dextran sulfate sodium (DSS) and relieve experimental colitis (Dong et al. 2021). Schaale et al. (2024) found that heme induces platelet cell death, characterized by increased ROS levels, phosphatidyl serine exposure, and loss of mitochondrial membrane potential, which is specifically regulated by FURIN. Increasing evidence suggests that furin plays a crucial role in the pathophysiological conditions of neurodegenerative and neuropsychiatric diseases (Zhang et al. 2022).

However, the exact role of FURIN in the pathogenesis of RLS remains unclear. The pathogenesis of RLS is complex. The primary theoretical framework underlying the pathophysiology of RLS is abnormal iron metabolism and dopaminergic dysfunction. In the brain, FURIN cleaves proprotein substrates, which include growth factor precursors, α‐ and β‐secretases, multiple matrix metalloproteinases (MMPs), and other enzymes and receptors (Zhang et al. 2022). MMP‐3 is upregulated in many pathological conditions, inducing neuroinflammation and cell apoptosis. In PD patients and animal models, elevated levels of MMP‐3 have been detected in dopaminergic (DA) neurons in the substantia nigra (Lorenzl et al. 2002). G protein‐coupled receptor 37 (GPR37) also participates in the dopaminergic signaling pathway by interacting with dopamine transporter in the presynaptic membrane of the striatum in mice, thereby regulating dopamine uptake (Marazziti et al. 2007). GPR37‐knockout mice also exhibit reduced dopamine levels in the striatum and specific motor deficits (Marazziti et al. 2004; Zhang et al. 2020). Choi DH and others suggested that treadmill exercise helps reduce symptoms of Alzheimer's disease in aged APP‐C105 mice, likely by fixing iron dyshomeostasis and enhancing furin expression, which helps process Amyloid‐β precursor protein (APP) through α‐secretase (Choi et al. 2021). These findings suggest that FURIN may influence iron metabolism and dopaminergic function by activating proprotein substrates, leading to RLS.

Previous studies on TLR1 have been limited, with earlier research only finding a significant positive correlation between TLR1 expression levels and ferroptosis genes, which matches our results (He et al. 2023). The transcriptomic analysis of RLS has confirmed that genetic susceptibility is a critical factor in the disease and provides significant biological evidence for its inflammatory nature (Mogavero et al. 2024). It highlights the significant involvement of infectious factors and the activation of various biological pathways (particularly IL‐17, TRP, NF‐κB, NLR, MAPK, p53, mitophagy, and ferroptosis) that are related to neurotransmitter mechanisms, synaptic plasticity, axon guidance, neurodegeneration, carcinogenesis, and metabolism (Mogavero et al. 2024). Human Toll‐like receptors (TLRs) are a class of transmembrane receptors that recognize patterns and are an essential component of innate immunity (Rusanen et al. 2017). The Toll‐like receptor (TLR) family plays a key role in controlling innate immune responses to pathogen recognition (Rusanen et al. 2017). TLR1 may reduce the incidence of RLS by exerting innate immune responses. It was shown by Kang et al. (2016) that TLR‐mediated innate immune responses are how glial cells react to prion virus infection in the early stages. Jia et al. (2022) found that Lactobacillus johnsonii can alleviate colitis by activating CD206(+) macrophages (IL‐10) through TLR1/2‐STAT3‐mediated pathways and also discovered that TLR1/2 is crucial for STAT3 activation and macrophage recognition of Lactobacillus johnsonii. These findings provide reliable support for our results.

To our knowledge, TLR1 has not been reported in previous RLS studies. In our research, the expression and function of FURIN may be closely correlated with TLR1. Mediation analysis demonstrated that TLR1 indirectly influences the risk of RLS by regulating the expression of FURIN. TLR1 may directly influence FURIN and affect the role of ferroptosis in RLS. Potential therapeutic opportunities may be represented by this regulatory mechanism.

There are several advantages to this study. To minimize biases and reverse causality that are frequently observed in observational studies, MR was implemented for causal inference. We employed MR and mediation analysis to validate the role of FURIN in RLS and to identify upstream regulators of ferroptosis. Our findings are more reliable and comprehensive due to this approach. In addition, we have used a large, representative dataset from the FinnGen database, which consists of 4599 RLS patients and 495,749 controls, which ensures that our results are robust and generalizable. We further strengthen the validity of our results by incorporating ferroptosis gene data from multiple independent studies.

There are some limitations to our study. We may be unable to generalize our findings to populations outside of European ancestry due to the fact that GWAS data are primarily based on data from individuals of European ancestry. Although our research offers valuable insights for the development of treatments, clinical trials are required to verify the findings. In the pathogenesis of RLS, these factors are likely to play a crucial role, and future research should explore these possibilities. A larger clinical trial is still needed to validate whether FURIN serves as a biomarker for early diagnosis or as a therapeutic target.

## 5 Conclusion

In summary, our study's comprehensive analysis revealed that TLR1 may encourage RLS by regulating FURIN. The result may be a new therapeutic target for further RLS study and offers novel ideas about the relationship between RLS and ferroptosis.

## Author Contributions

MX: This author helped design the study and wrote the paper. CQS: This author helped perform statistical analysis and do the work of data collecting. All authors have read the manuscript and approved the final paper submitted.

## Conflicts of Interest

The authors declare no competing interests.

## Funding

The authors have nothing to report.

## Supporting information



Supplementary Figures: brb371038‐sup‐0001‐Figures.doc

Supplementary Tables: brb371038‐sup‐0002‐Tables.xls

## Data Availability

All data used in this study are publicly available and listed in Table S1. The GWAS statistics for RLS were obtained from the FinnGen database (https://www.finngen.fi/fi). The ferroptosis genes are derived from pQTL (pQTL, https://www.decode.com/summarydata/). The upstream genes are derived from the UK Biobank Proteomics Project (UKB‐PPP, https://www.synapse.org/Synapse:syn51364943/wiki/622119).
