# Supplementary material for: TLR1‐Regulated Ferroptosis Gene Decreases the Occurrence of Restless Legs Syndrome
Source: Brain Behav. 2025 Nov 11;15(11):e71038. doi: 10.1002/brb3.71038 (PMC12605992; doi:10.1002/brb3.71038)
Supplement: Supplementary file 1 — Supplementary Figures: brb371038‐sup‐0001‐Figures.doc [file BRB3-15-e71038-s001.doc]

**MR Analysis of the Relationship Between ferroptosis and RLS**

| **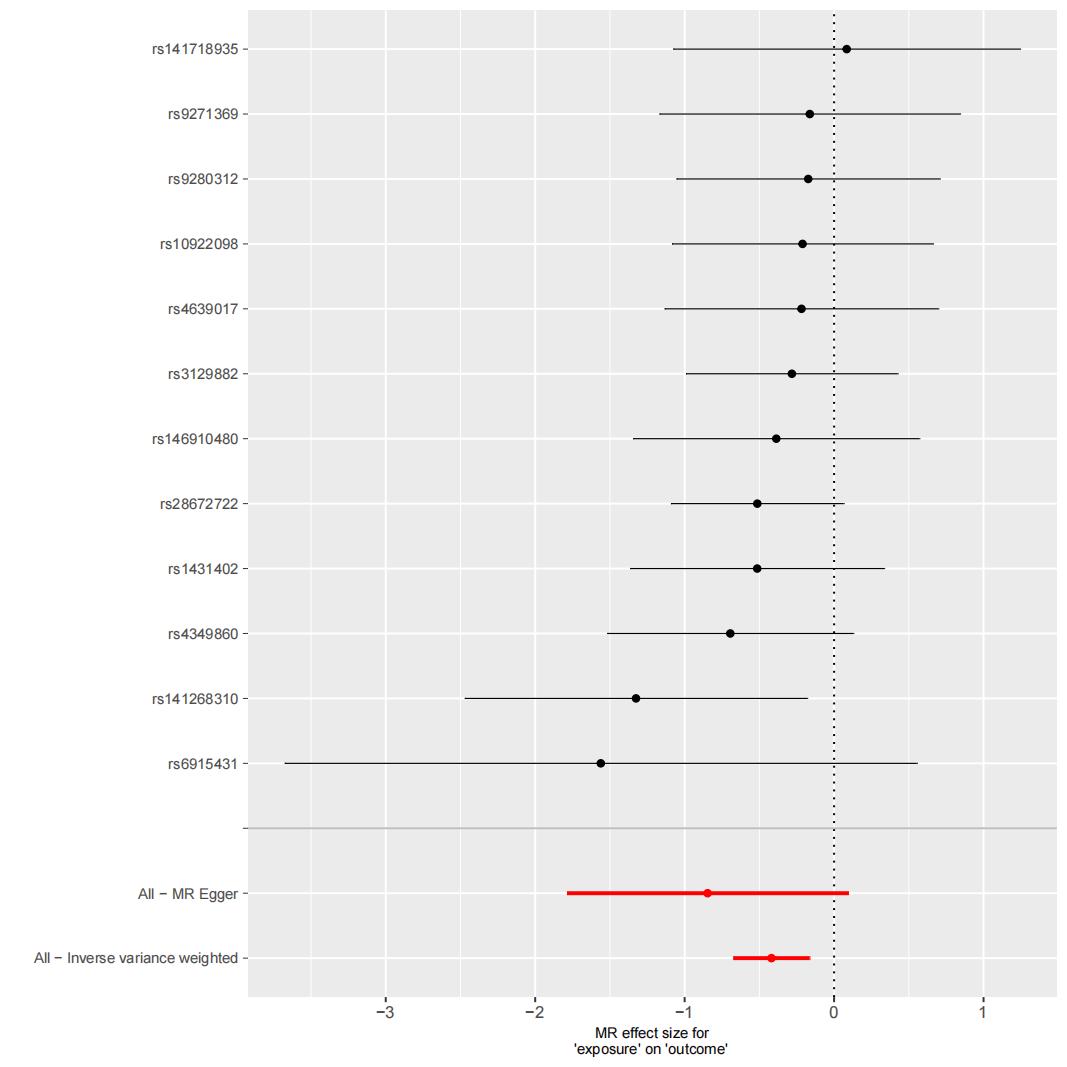**  **a** | **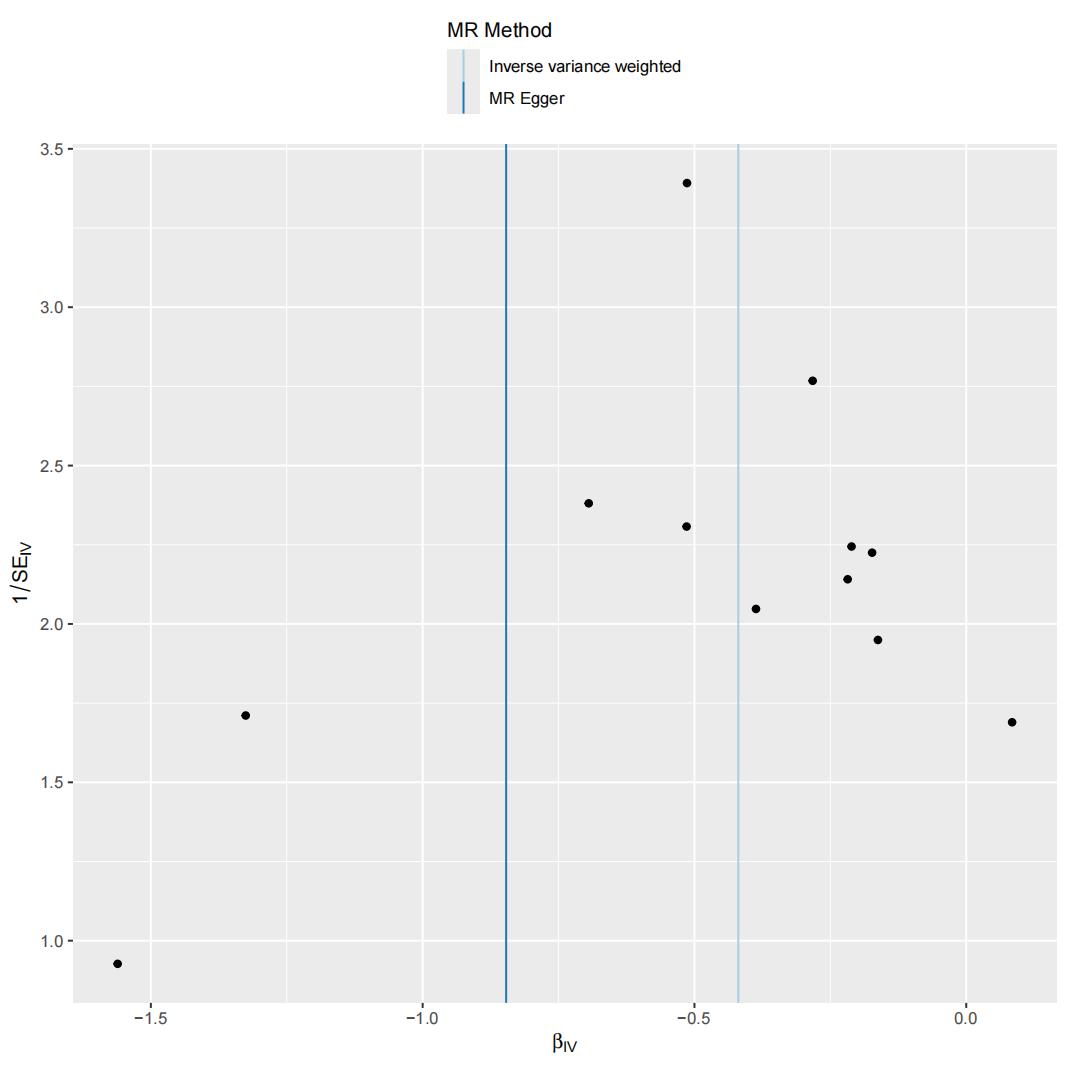**  **c** |
| --- | --- |
| **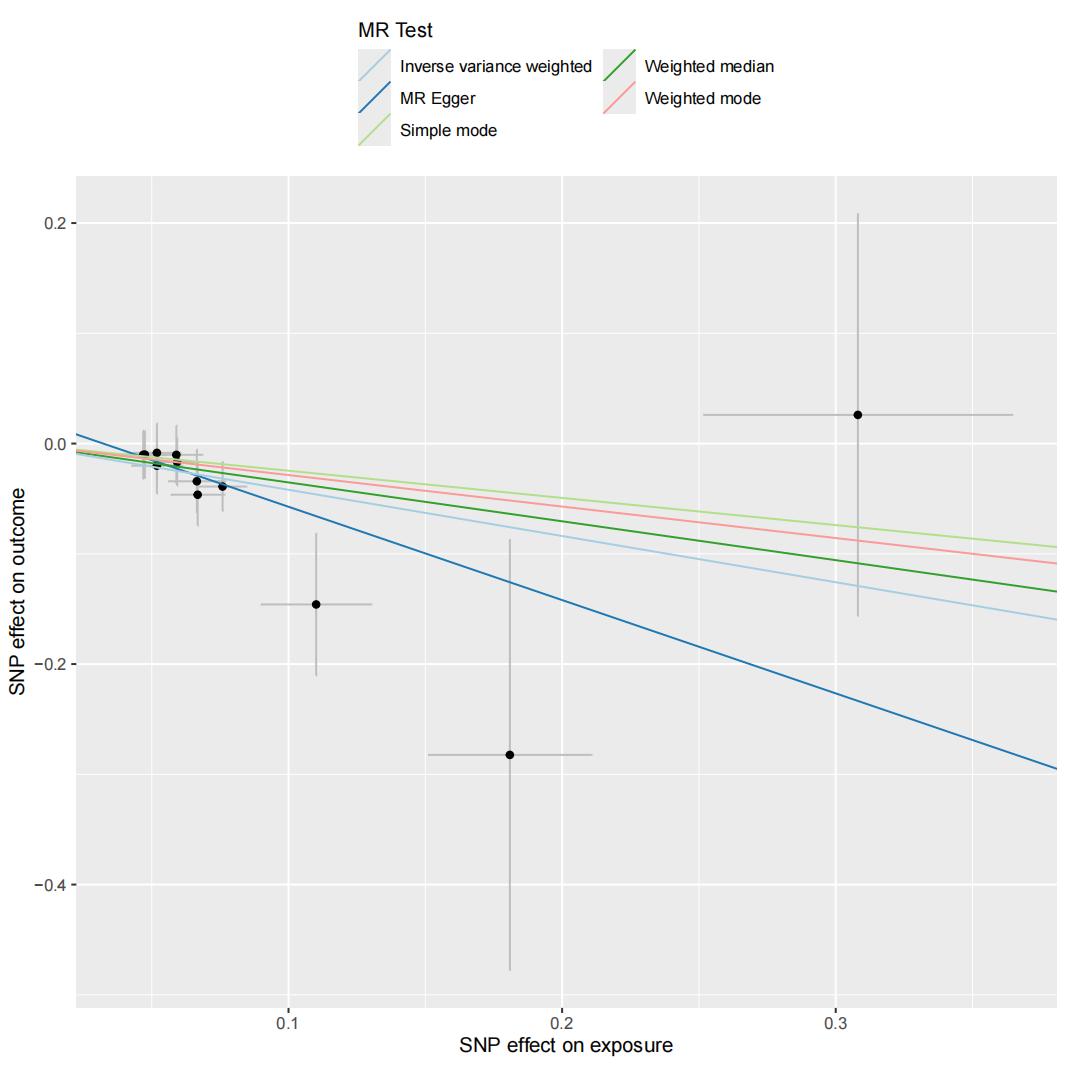**  **b** | **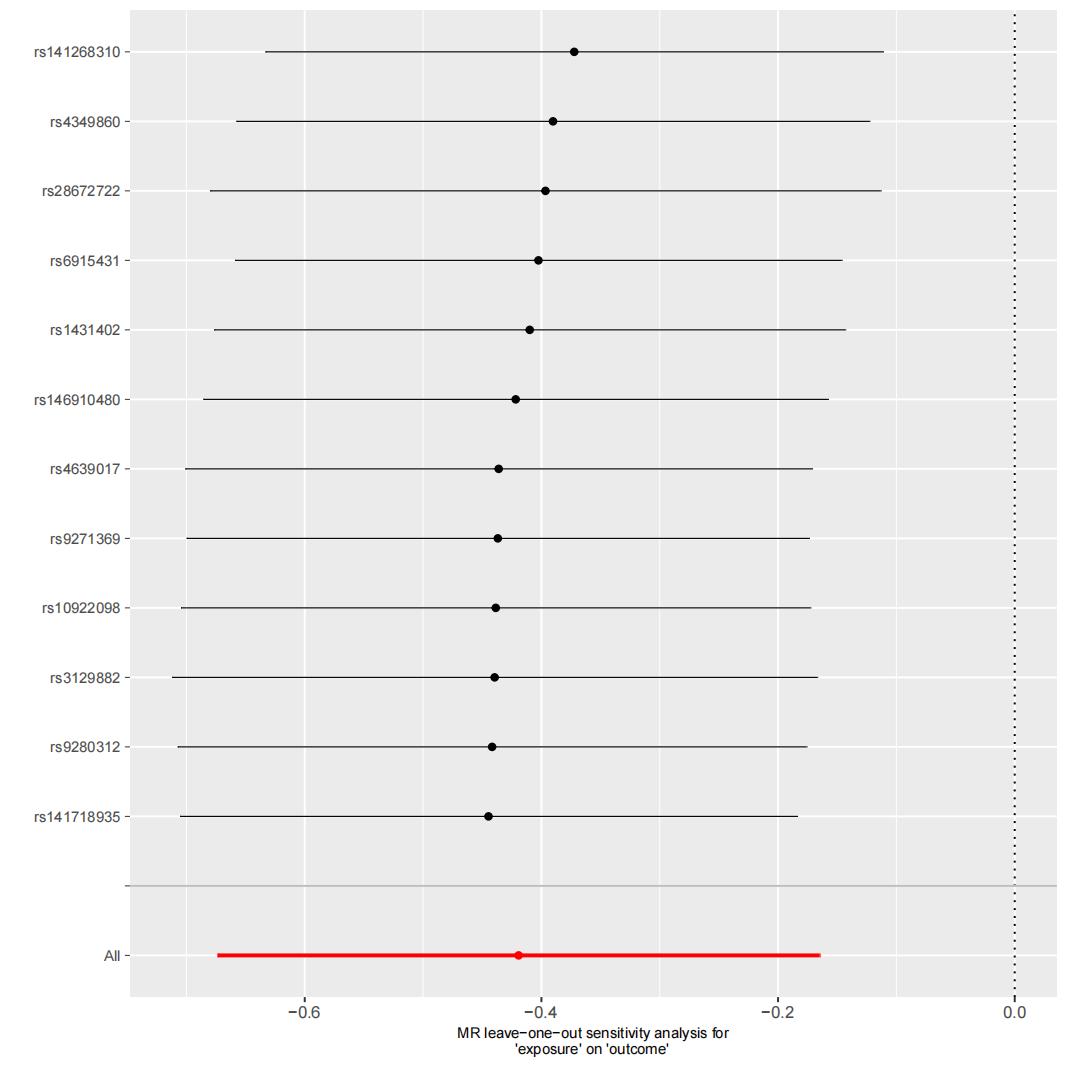**  **d** |

Supplementary Figure S1 Forest plot (a), scatter plot(b), funnel plot (c) and sensitivity analysis (d) of SNPs associated with FURIN on RLS.

| 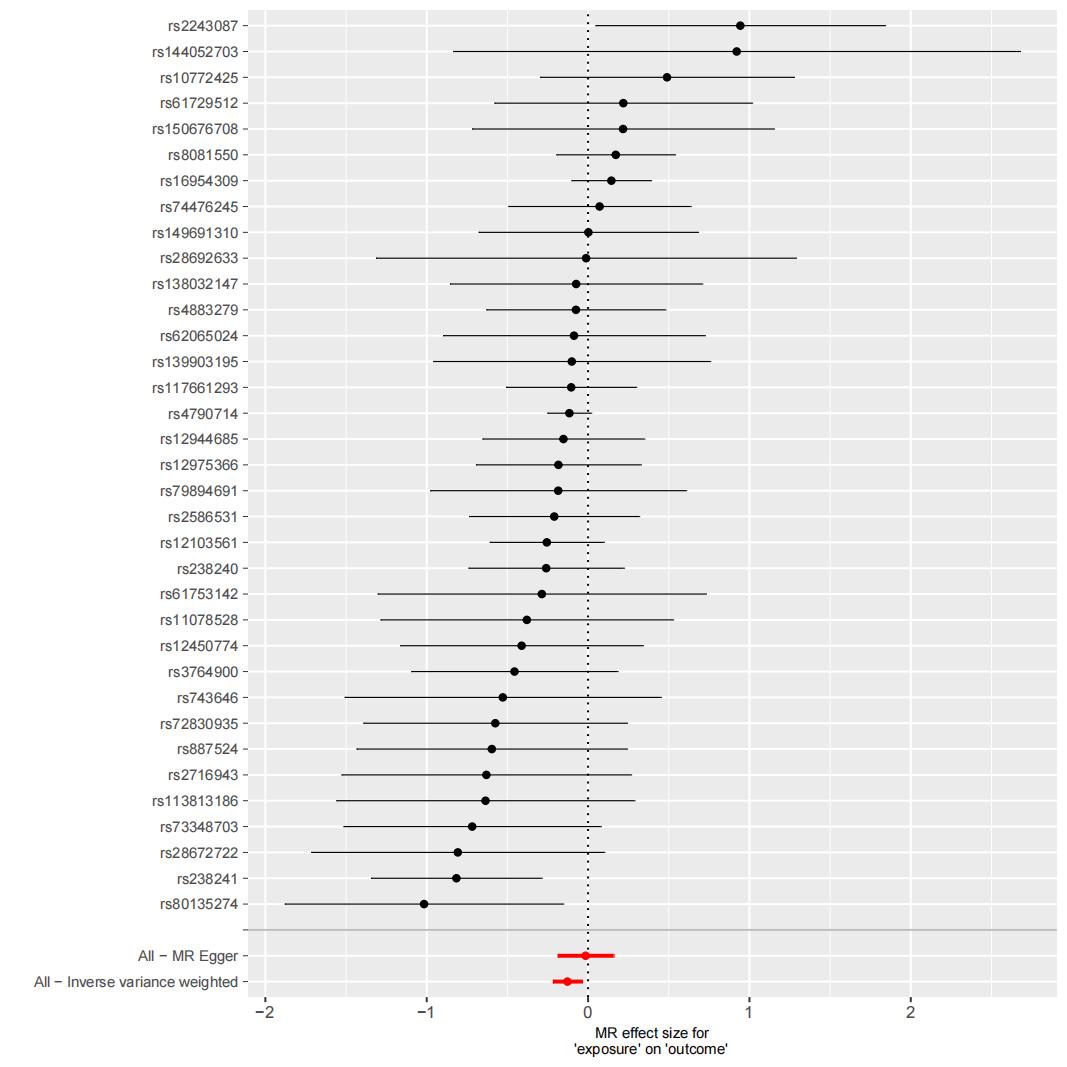  a | 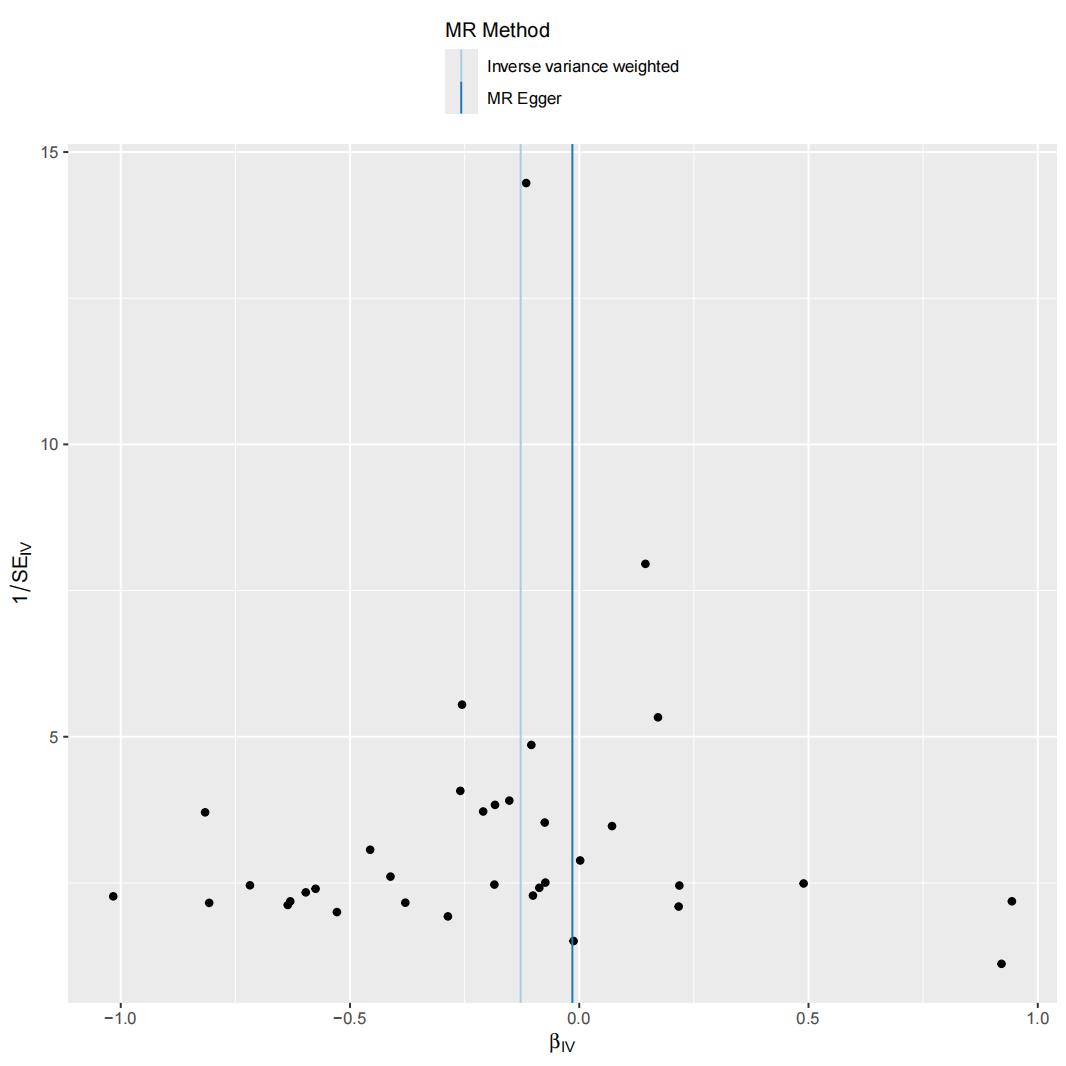  c |
| --- | --- |
| 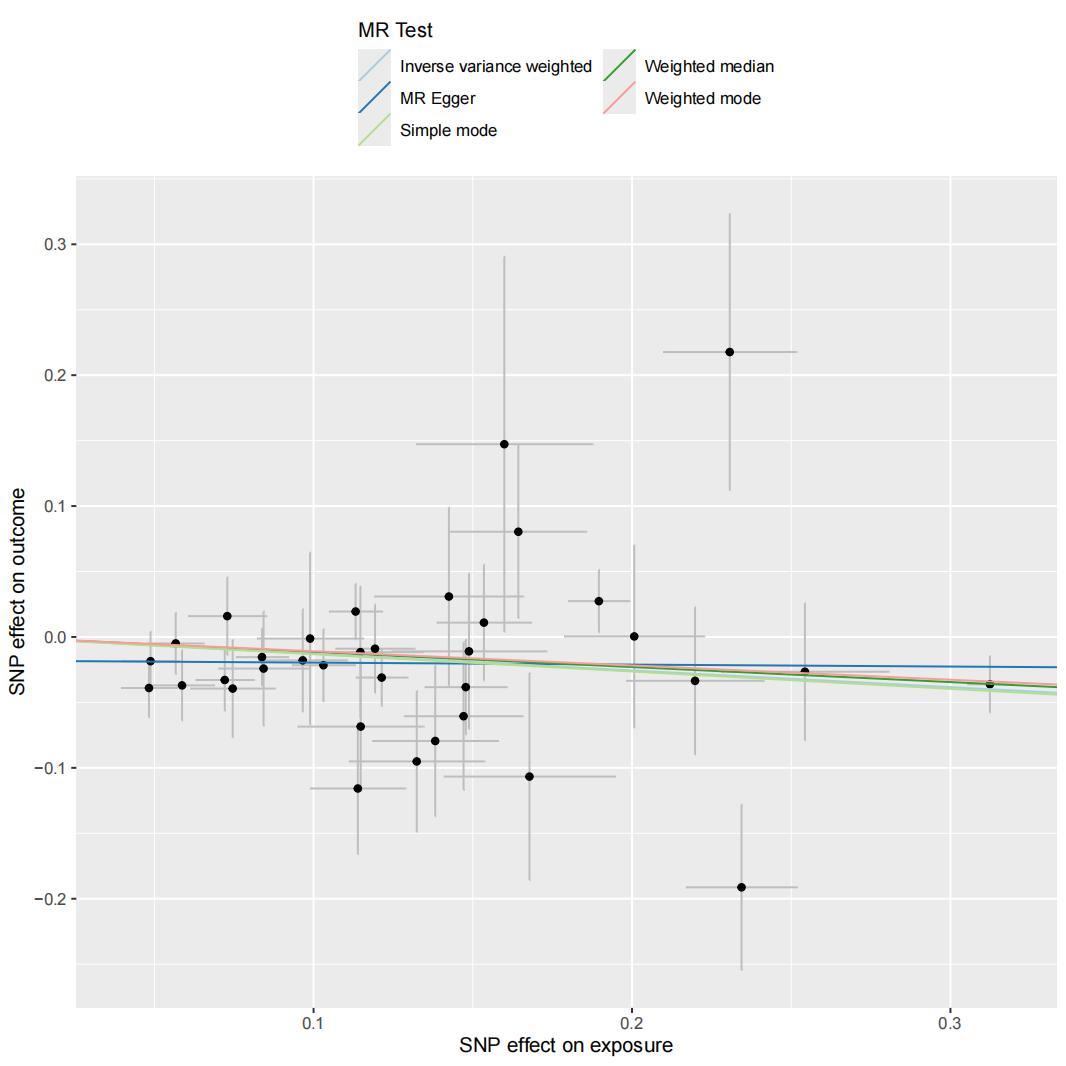  b | 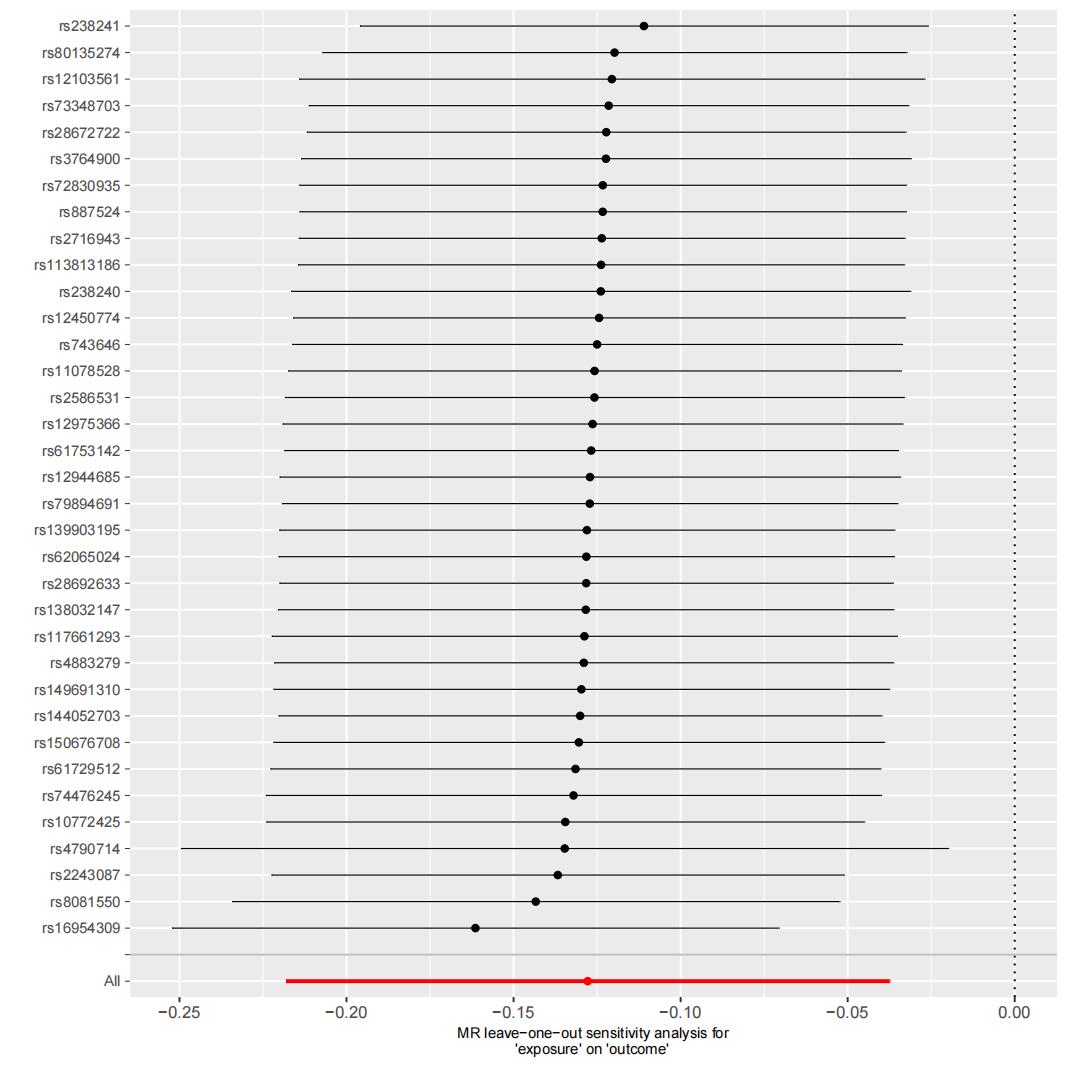  d |

Supplementary Figure S2 Forest plot (a), scatter plot(b), funnel plot (c) and sensitivity analysis (d) of SNPs associated with ENO3 on RLS.

**MR Analysis of the Relationship Between upstream genes and RLS**

| 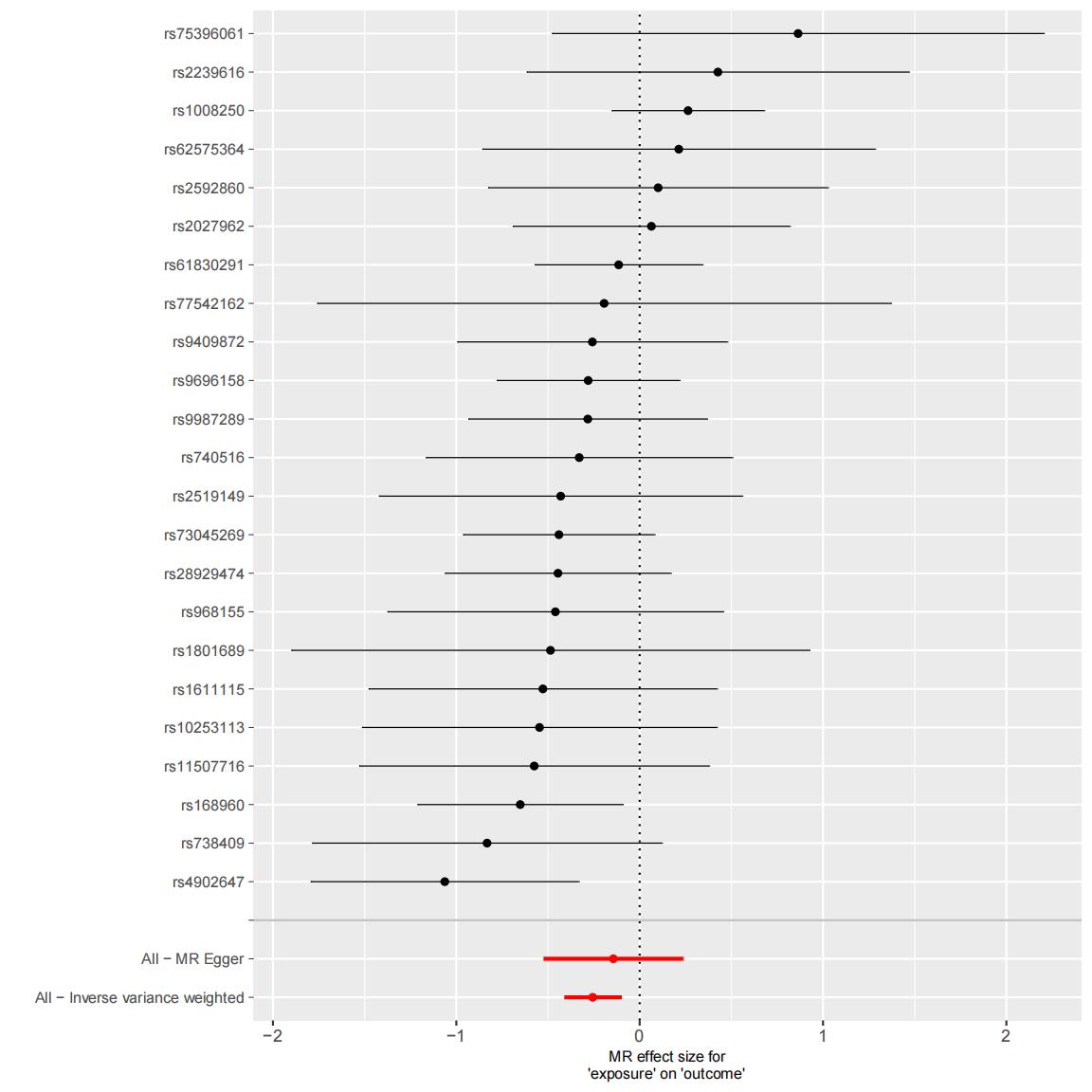  a | 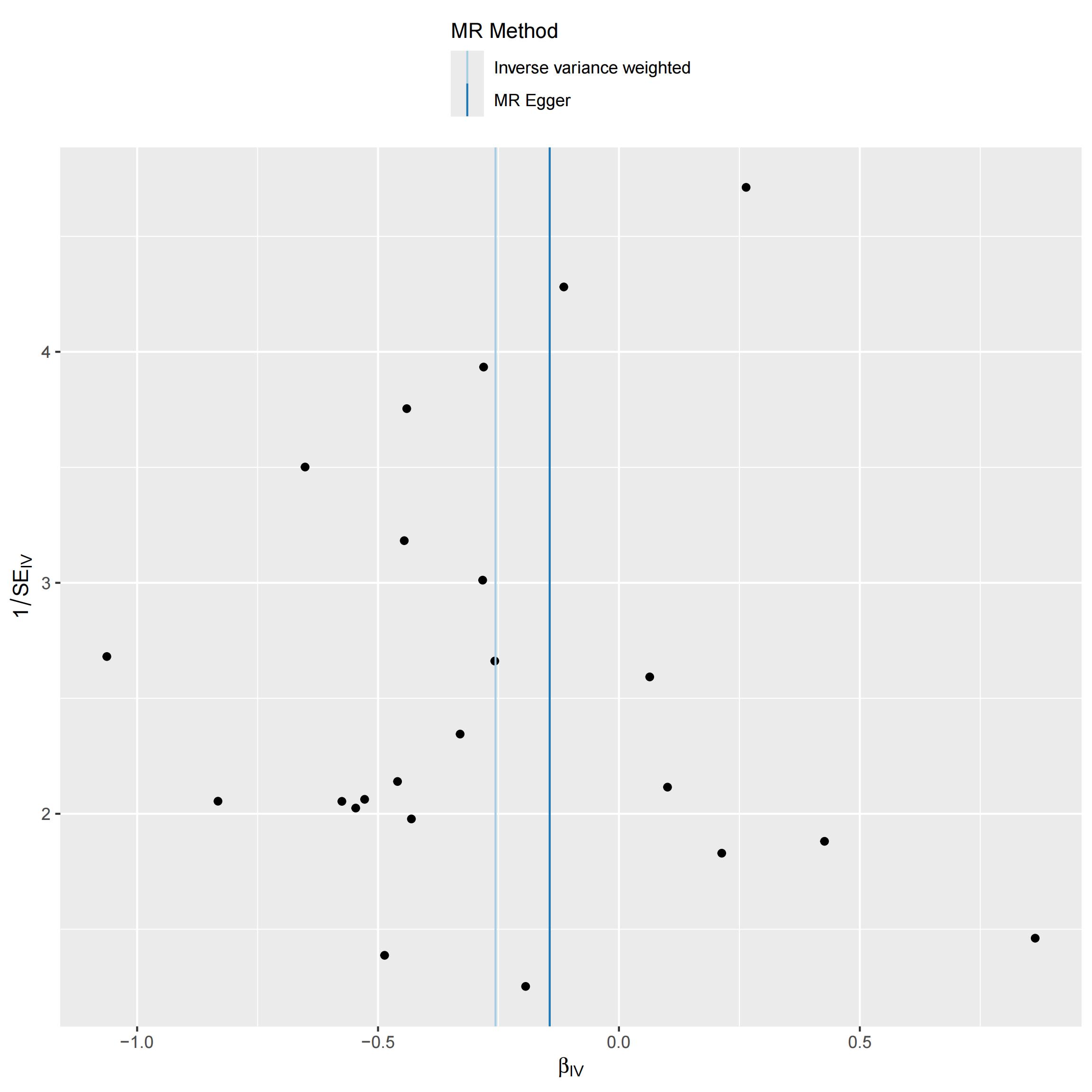  c |
| --- | --- |
| 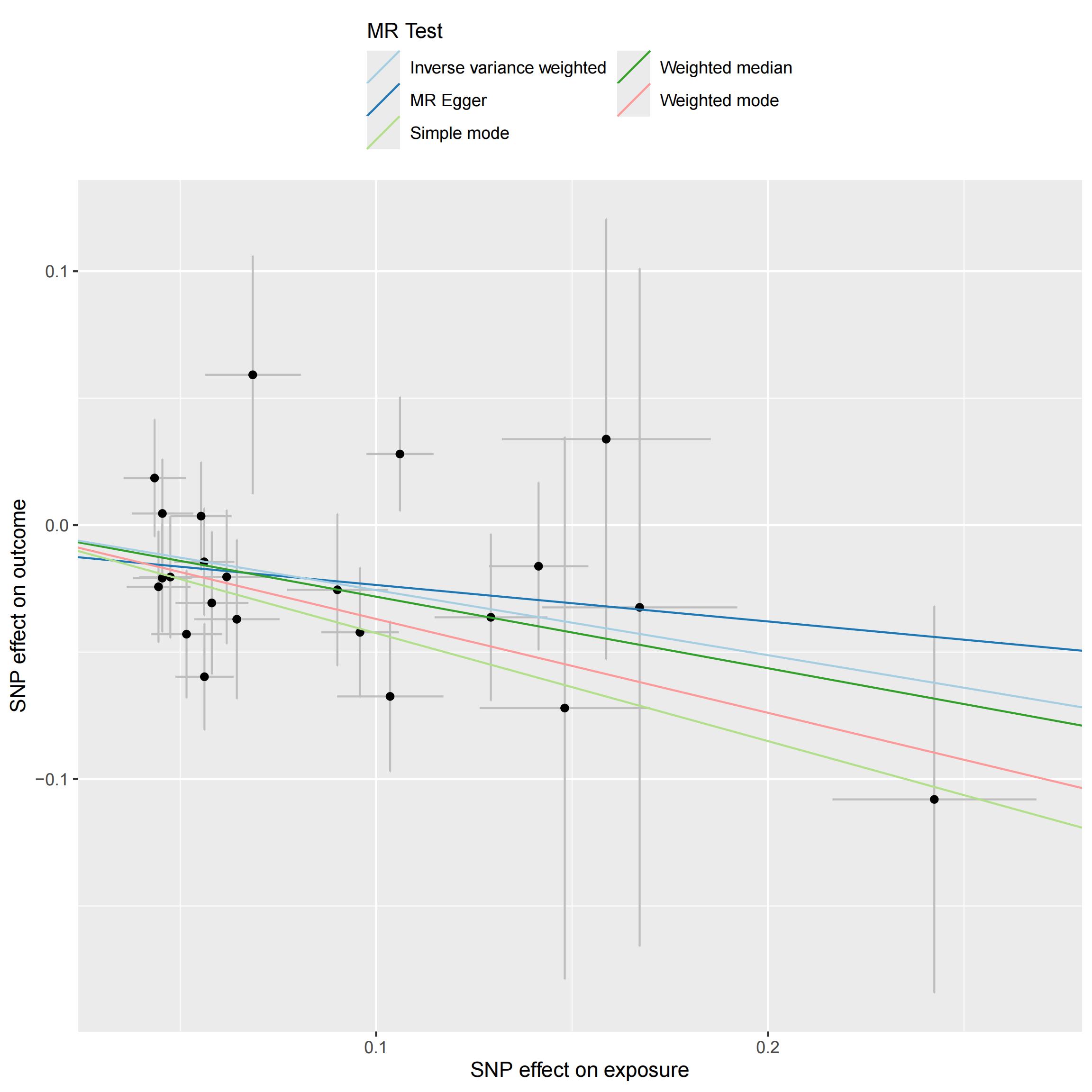  b | 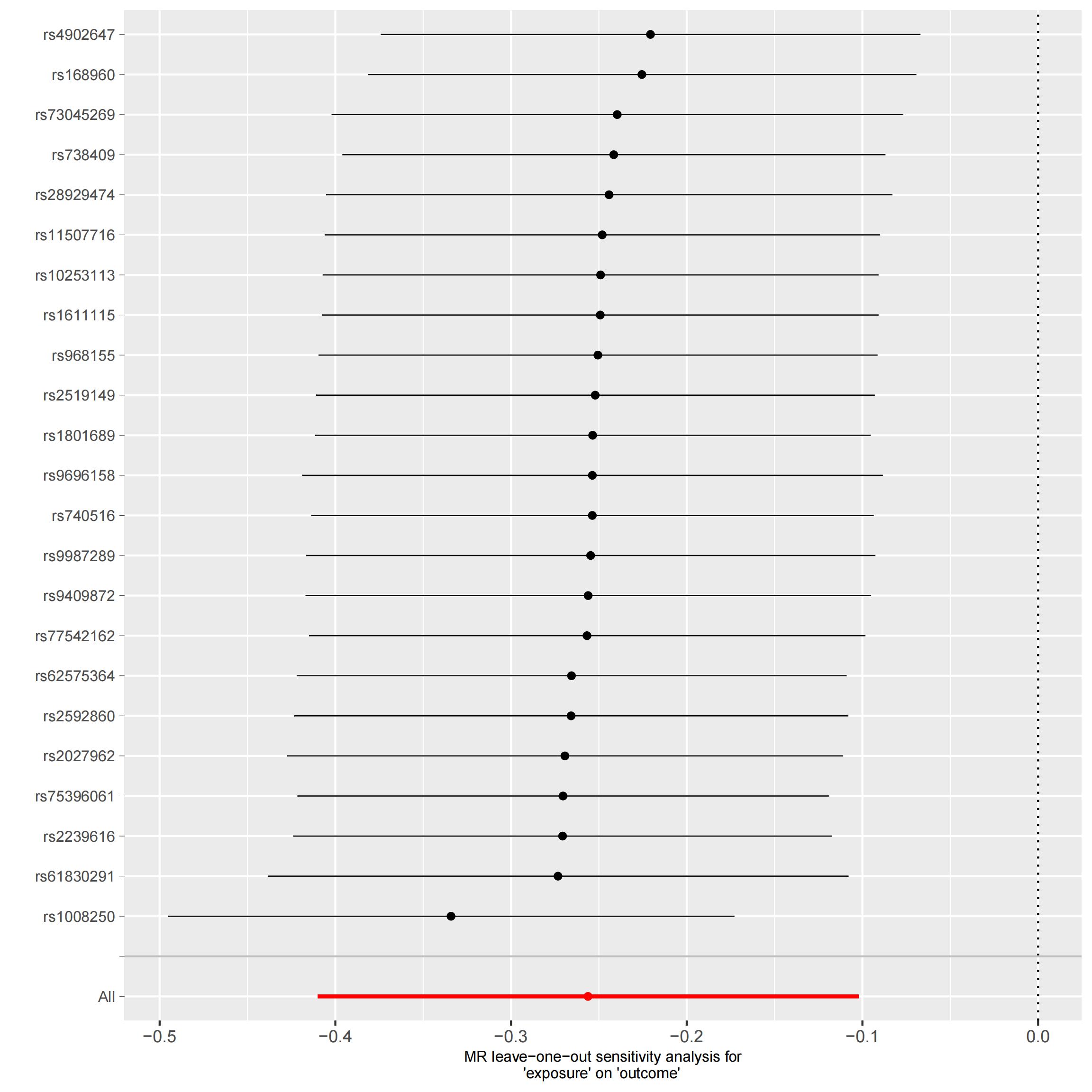  d |

Supplementary Figure S3 Forest plot (a), scatter plot(b), funnel plot (c) and sensitivity analysis (d) of SNPs associated with ADAMTSL2 on RLS.

| 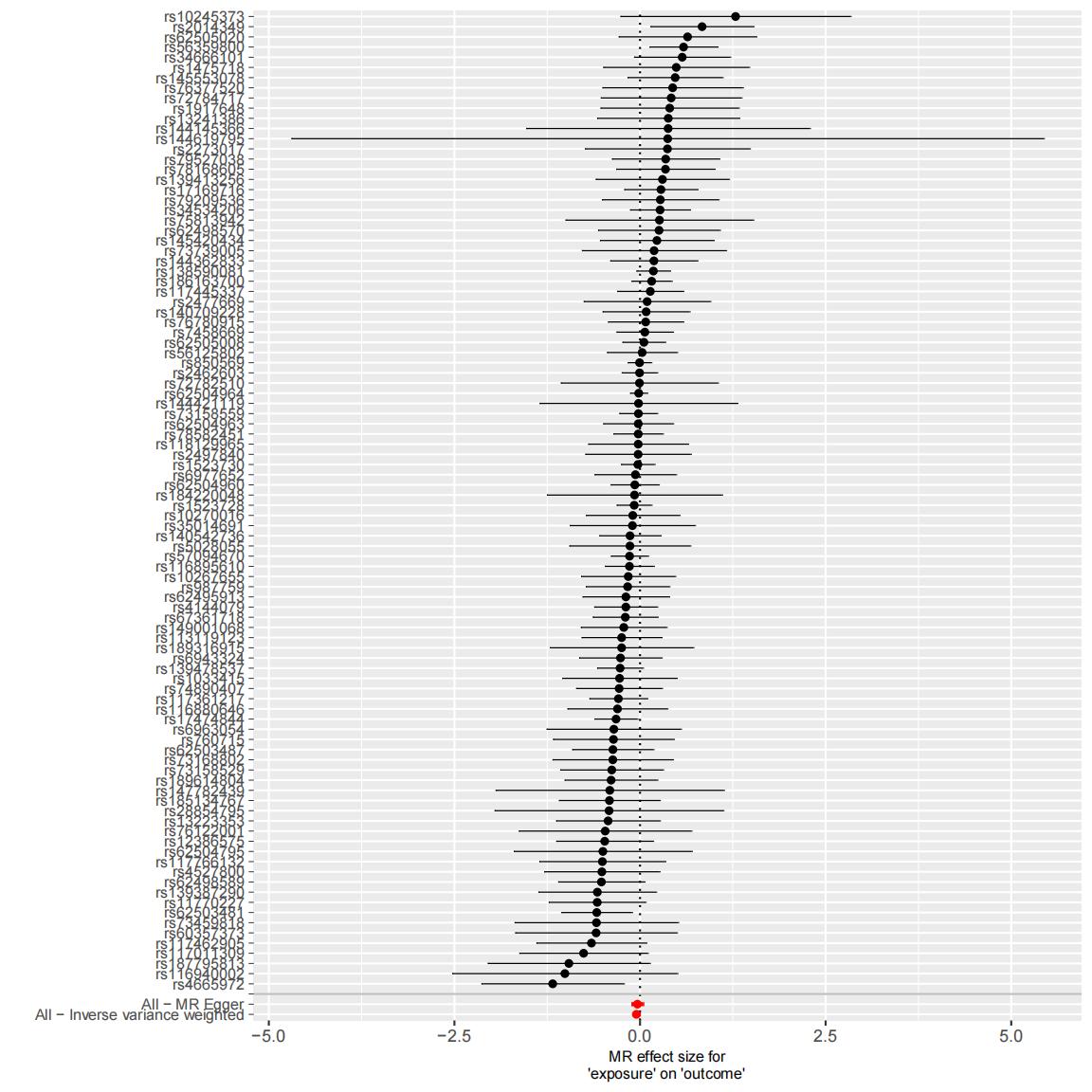  a | 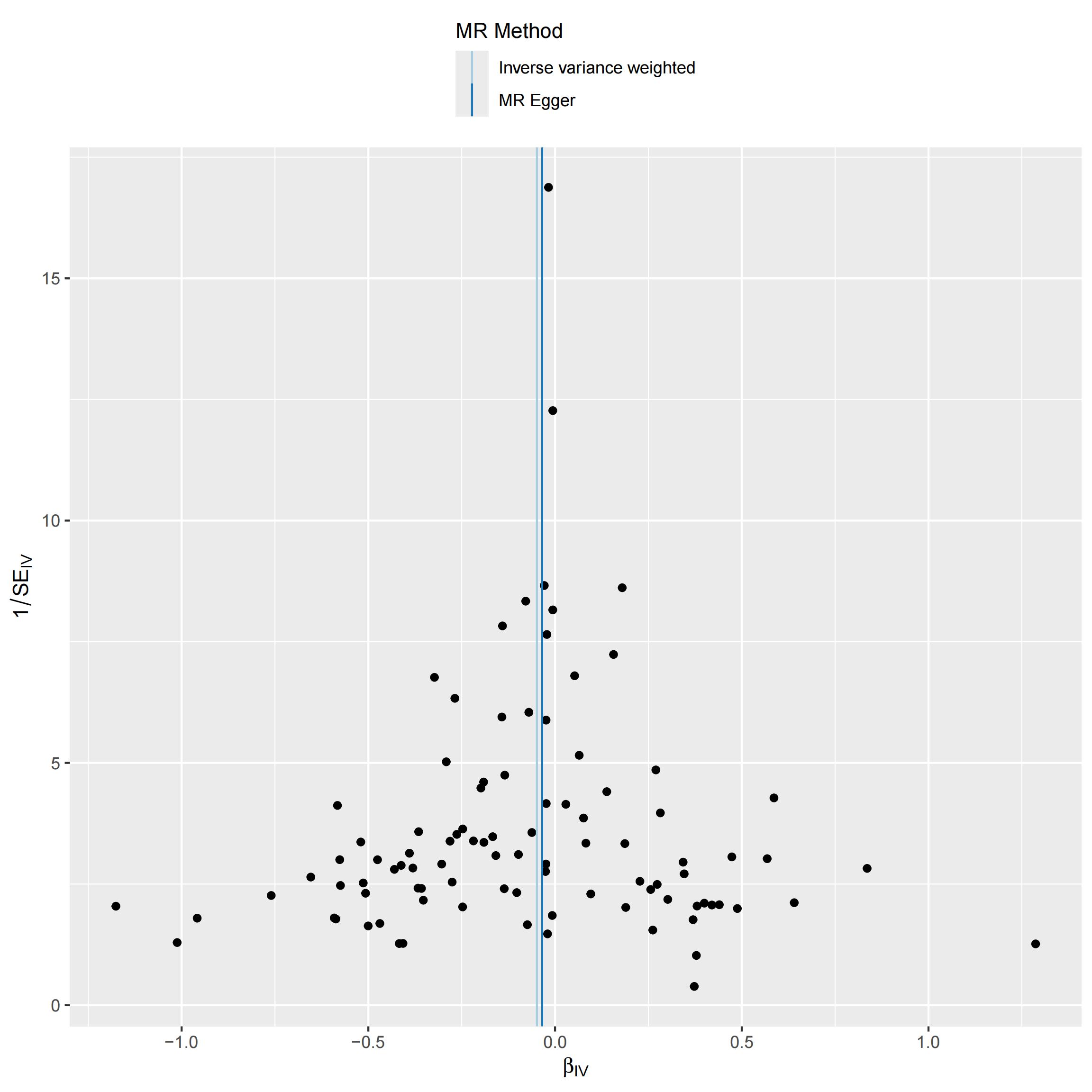  c |
| --- | --- |
| 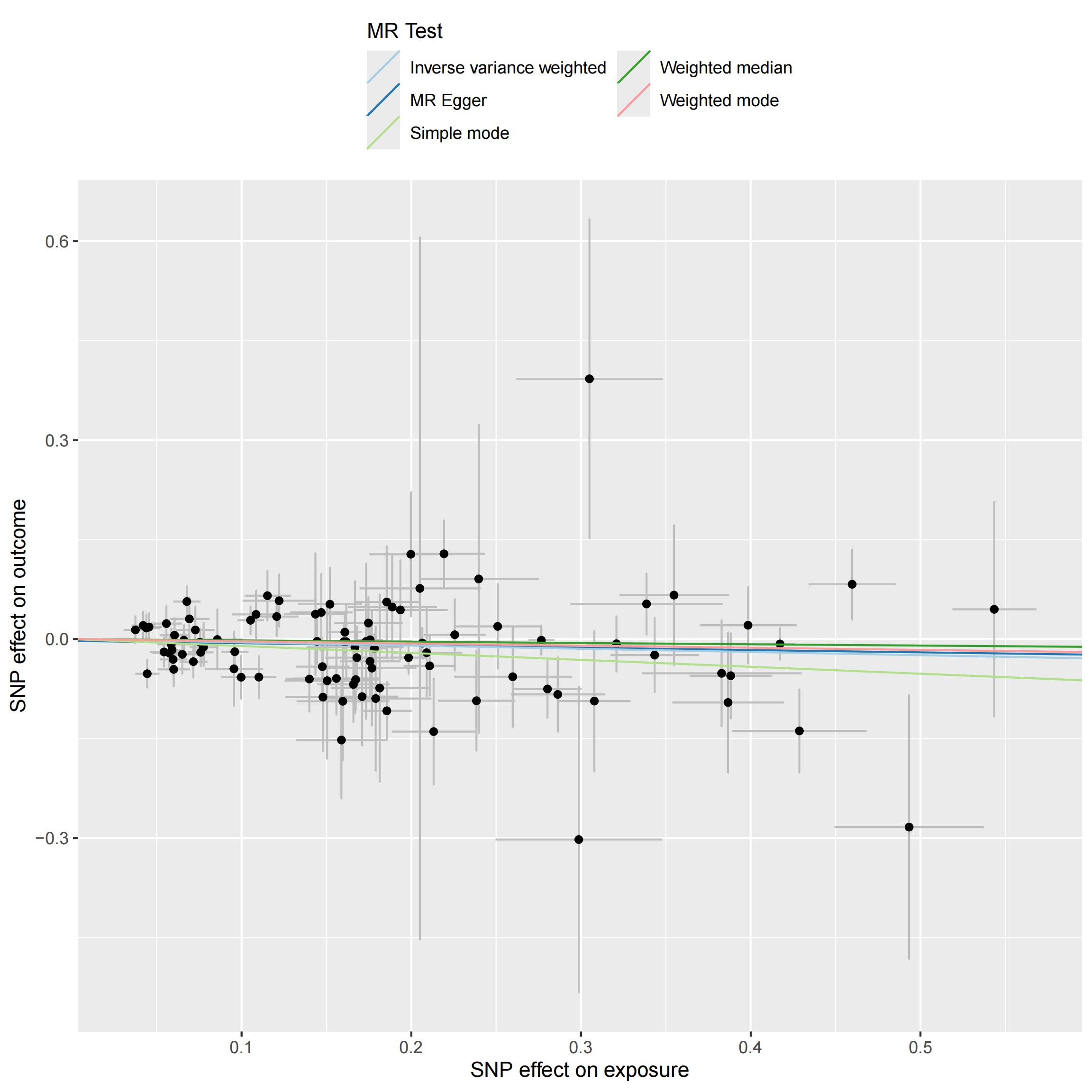  b | 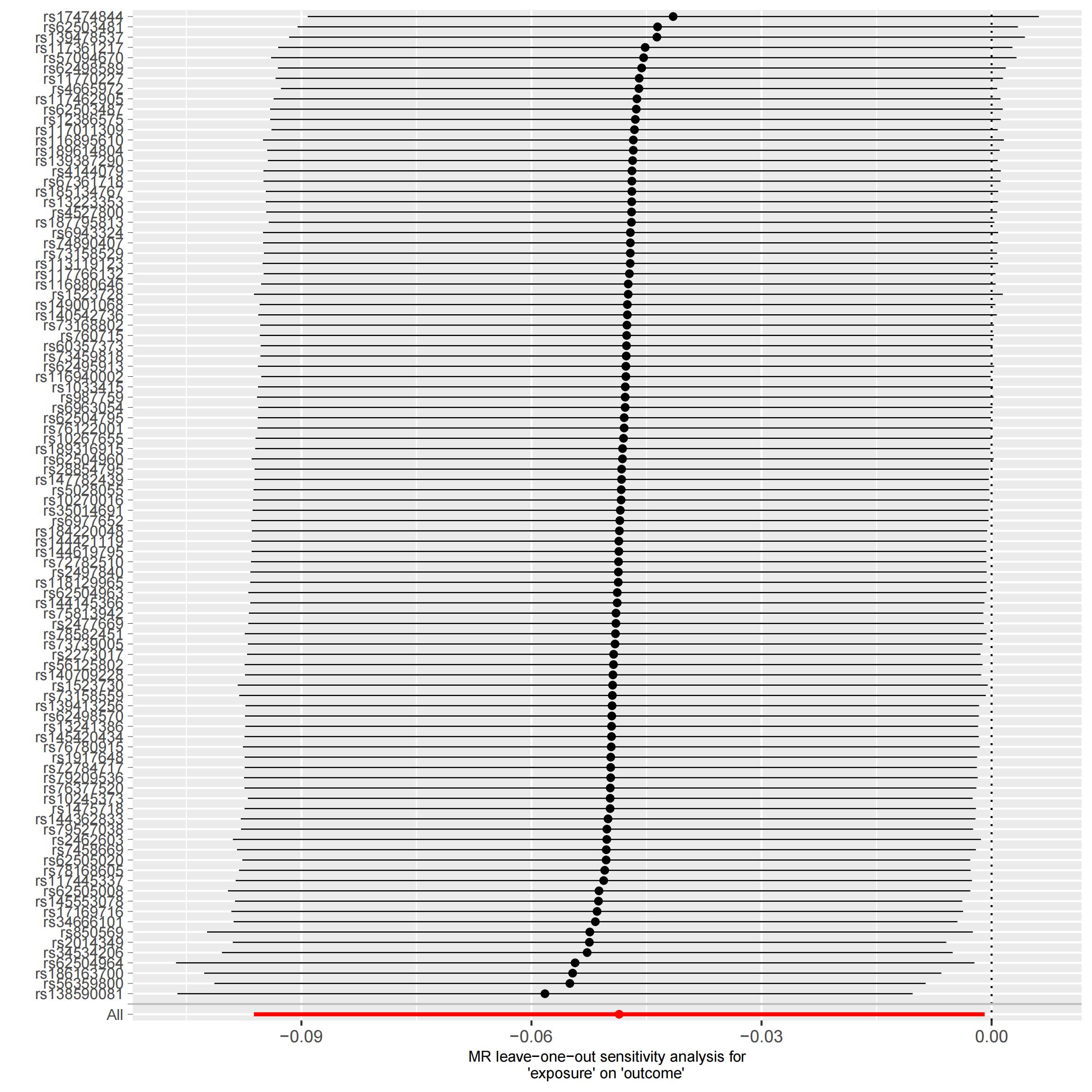  d |

Supplementary Figure S4 Forest plot (a), scatter plot(b), funnel plot (c) and sensitivity analysis (d) of SNPs associated with CNTNAP2 on RLS.

| 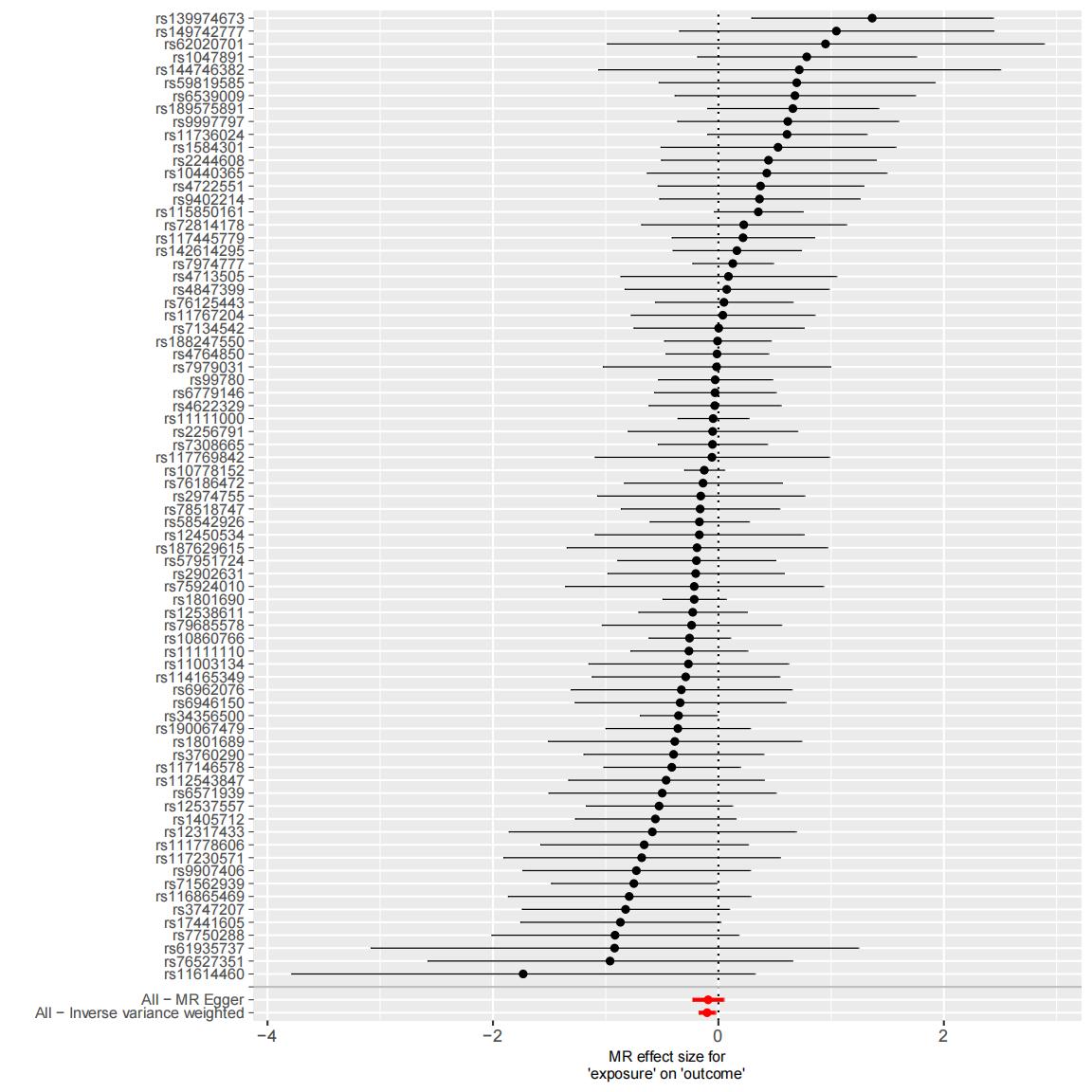  a | 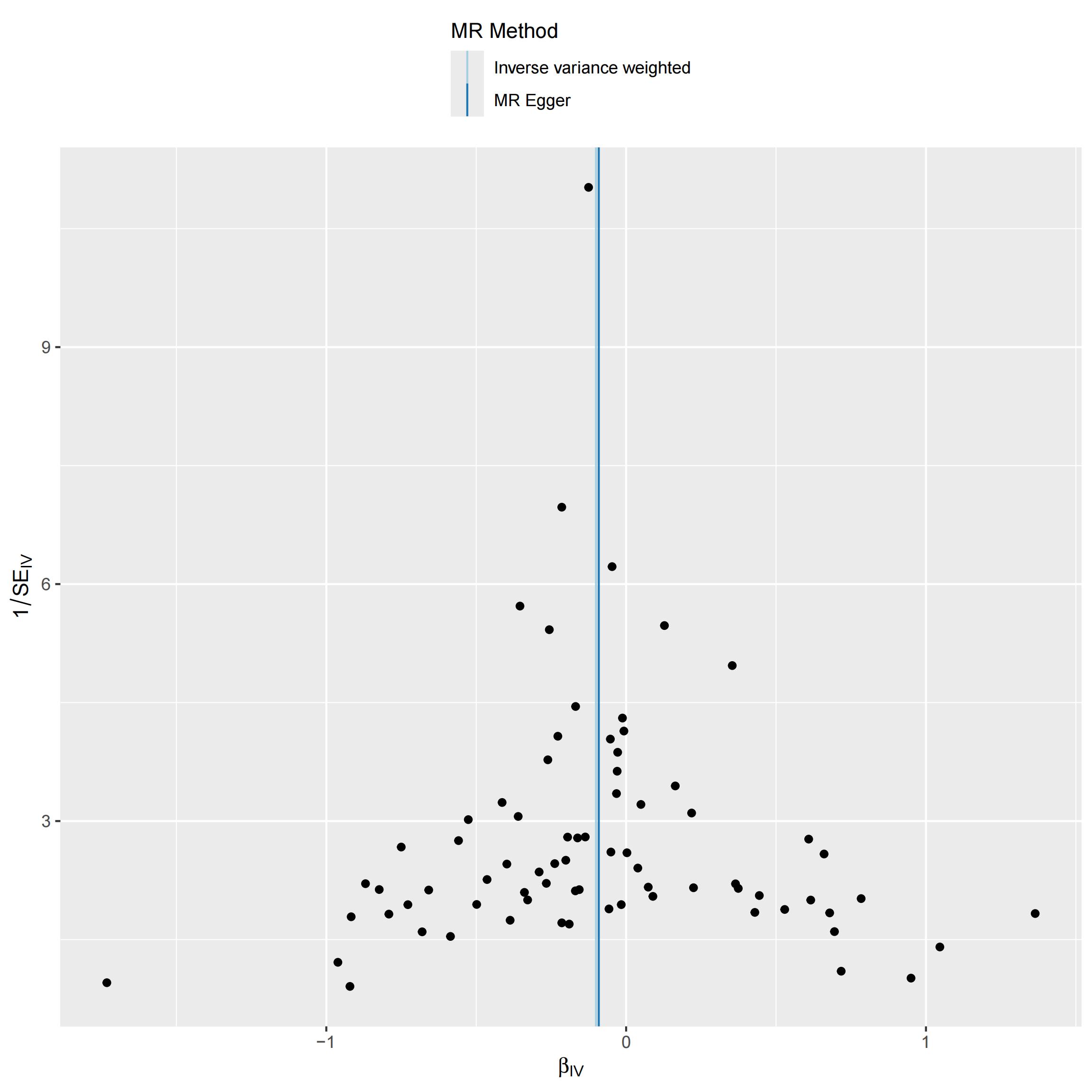  c |
| --- | --- |
| 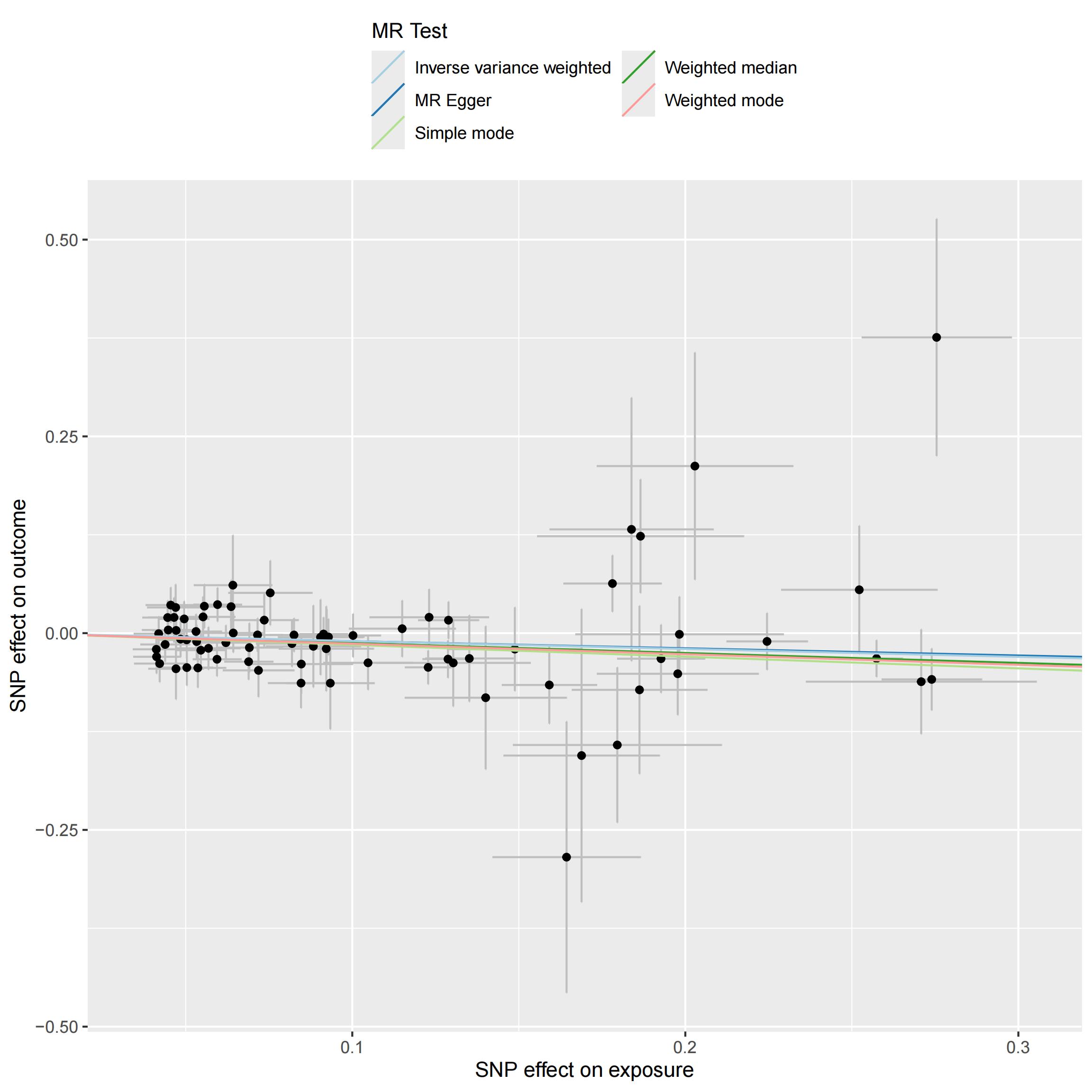  b | 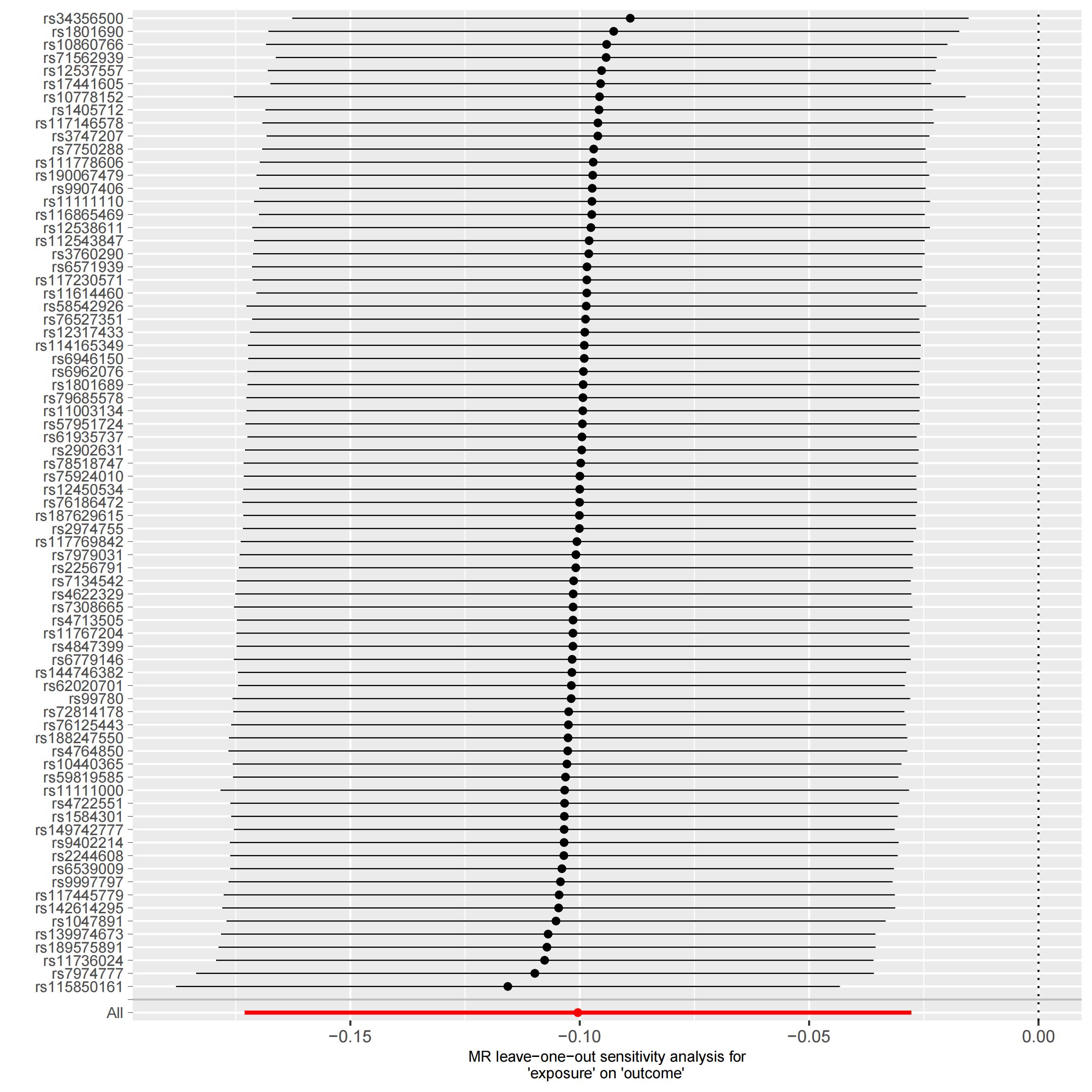  d |

Supplementary Figure S5 Forest plot (a), scatter plot(b), funnel plot (c) and sensitivity analysis (d) of SNPs associated with GUSB on RLS.

| 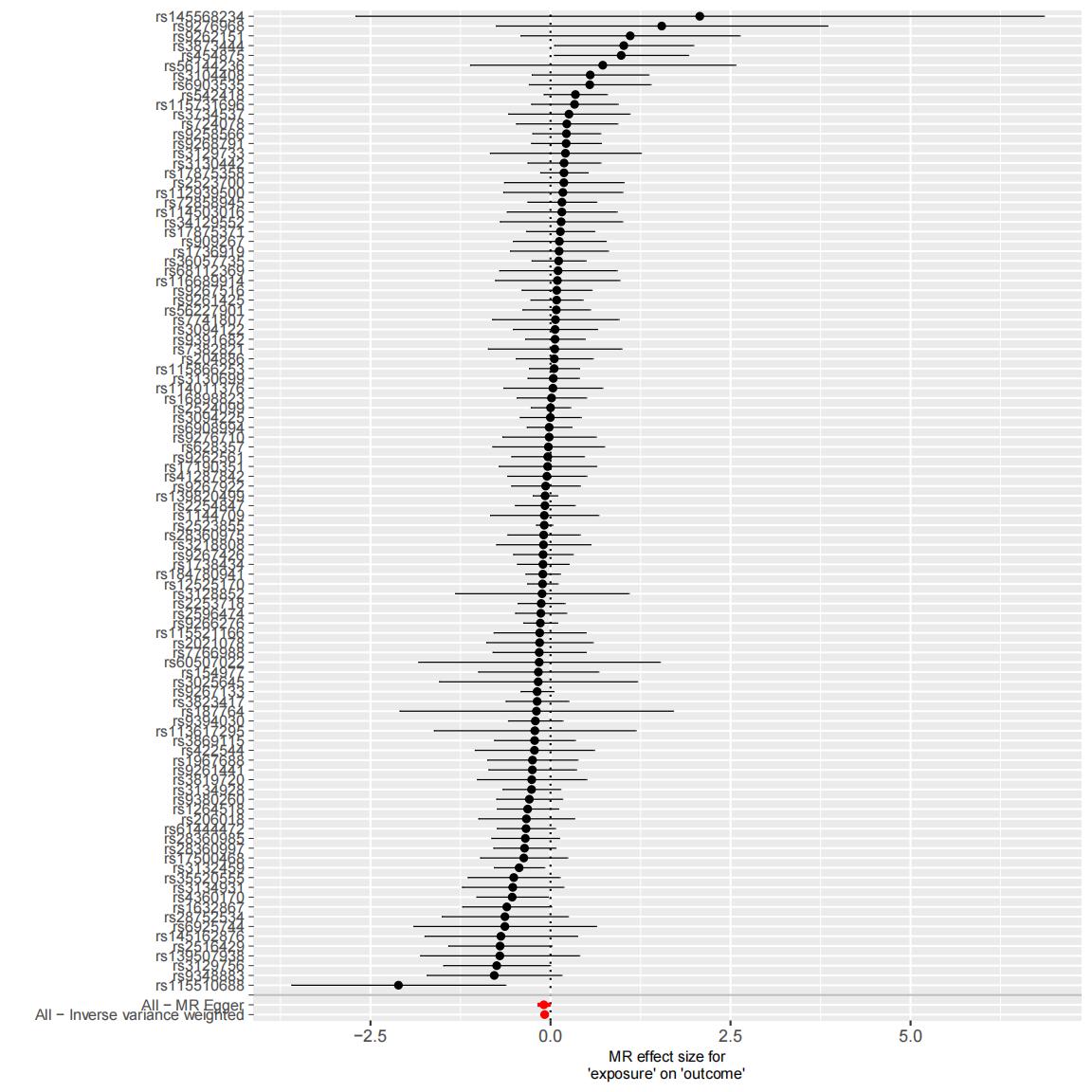  a | 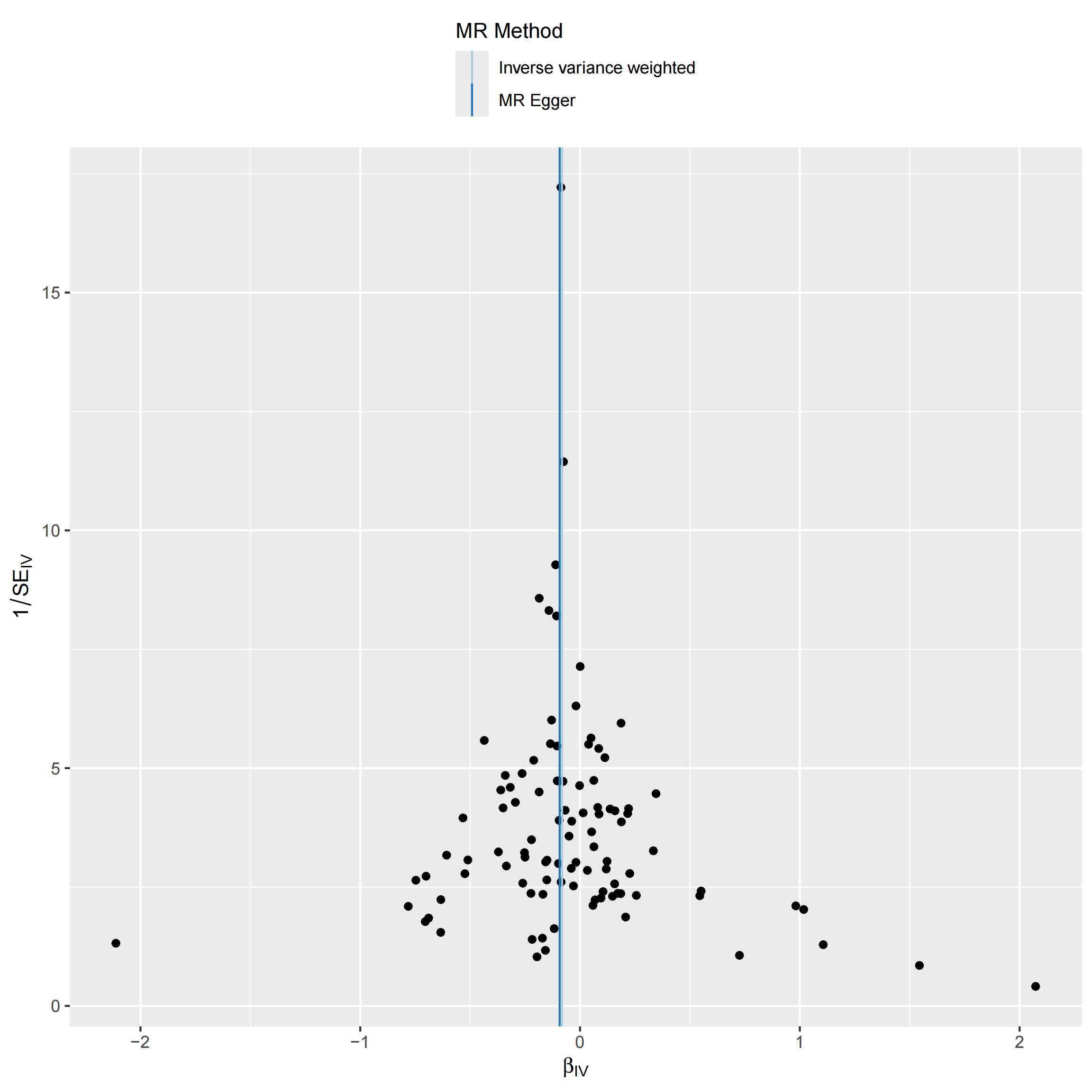  c |
| --- | --- |
| 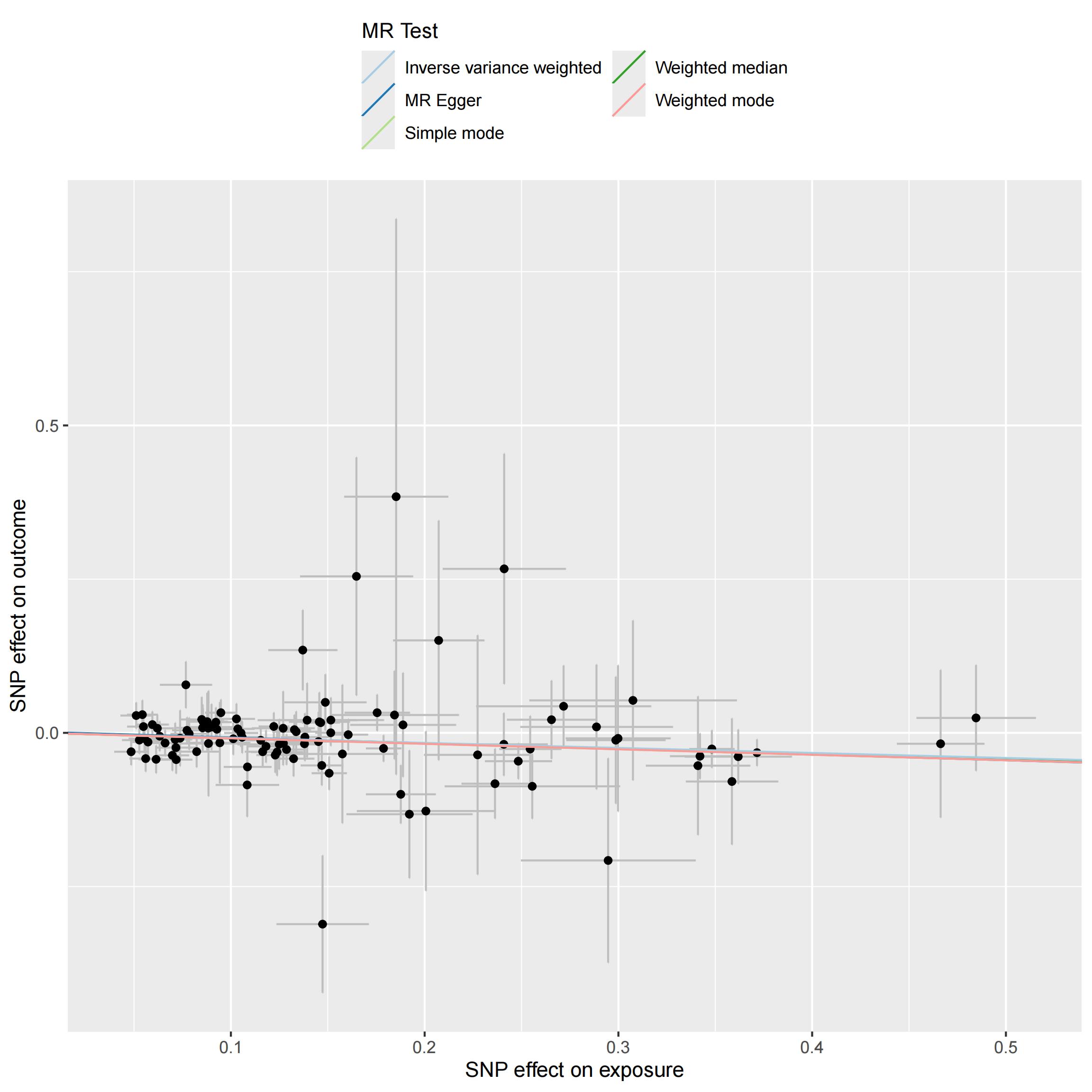  b | 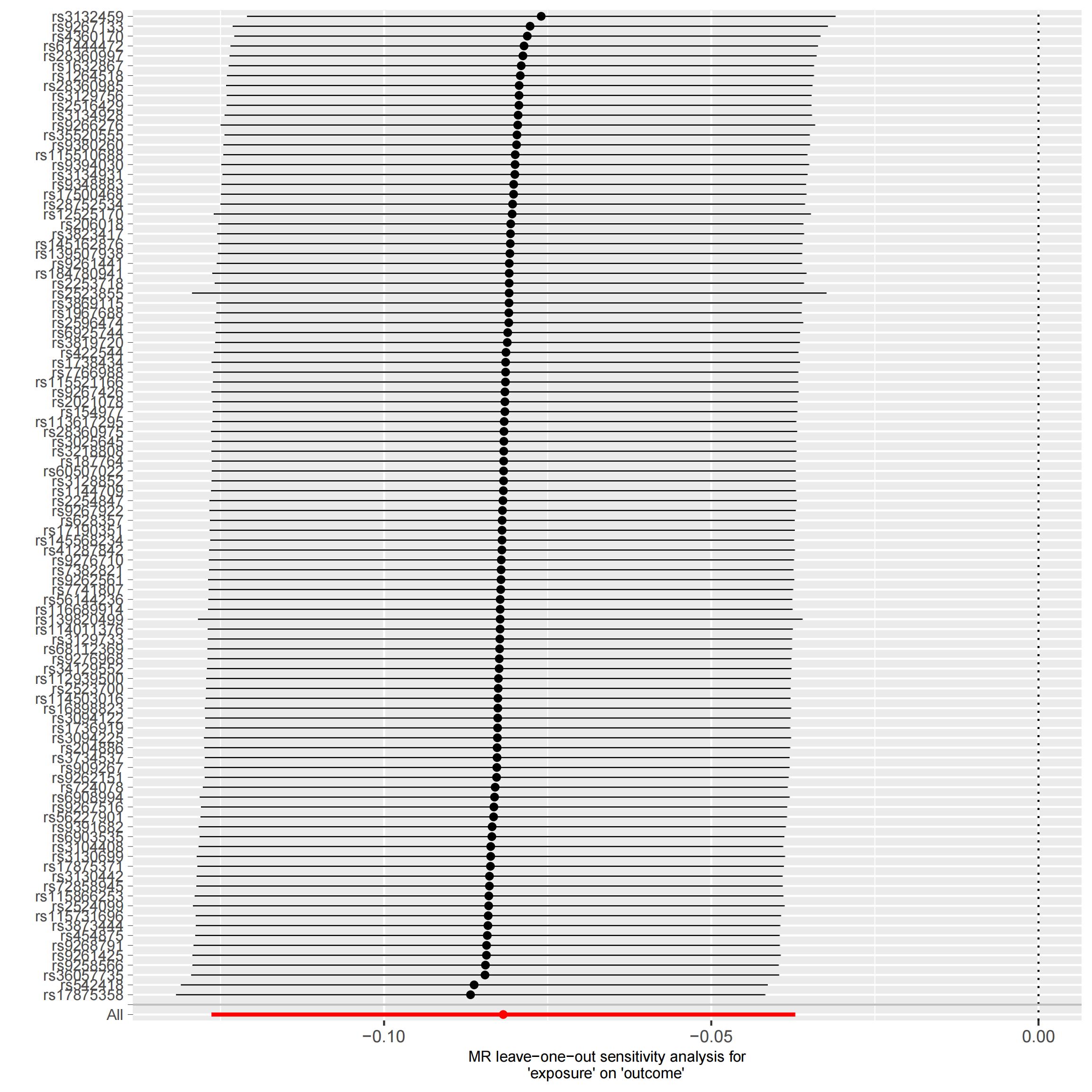  d |

Supplementary Figure S6 Forest plot (a), scatter plot(b), funnel plot (c) and sensitivity analysis (d) of SNPs associated with HCG22 on RLS.

| 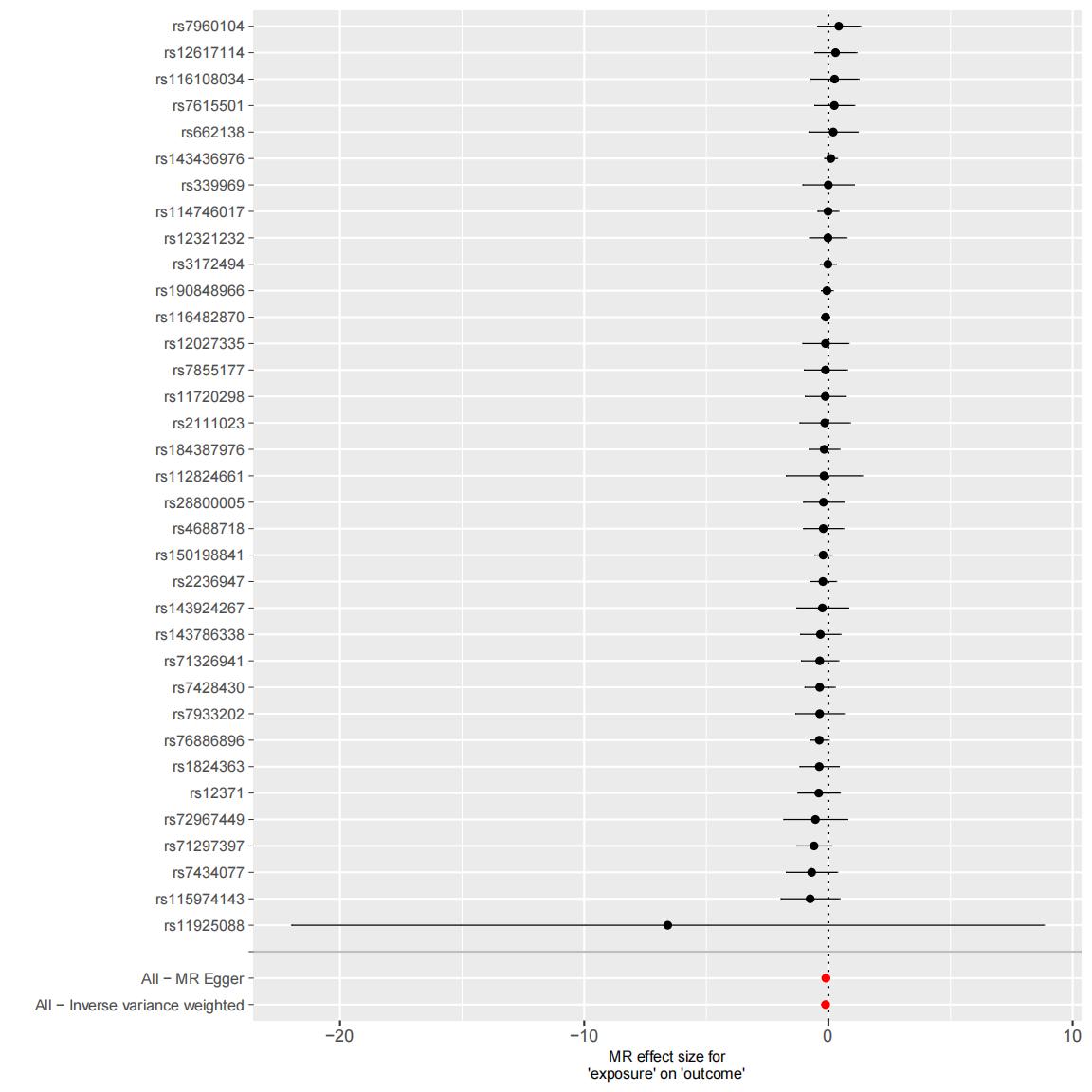  a | 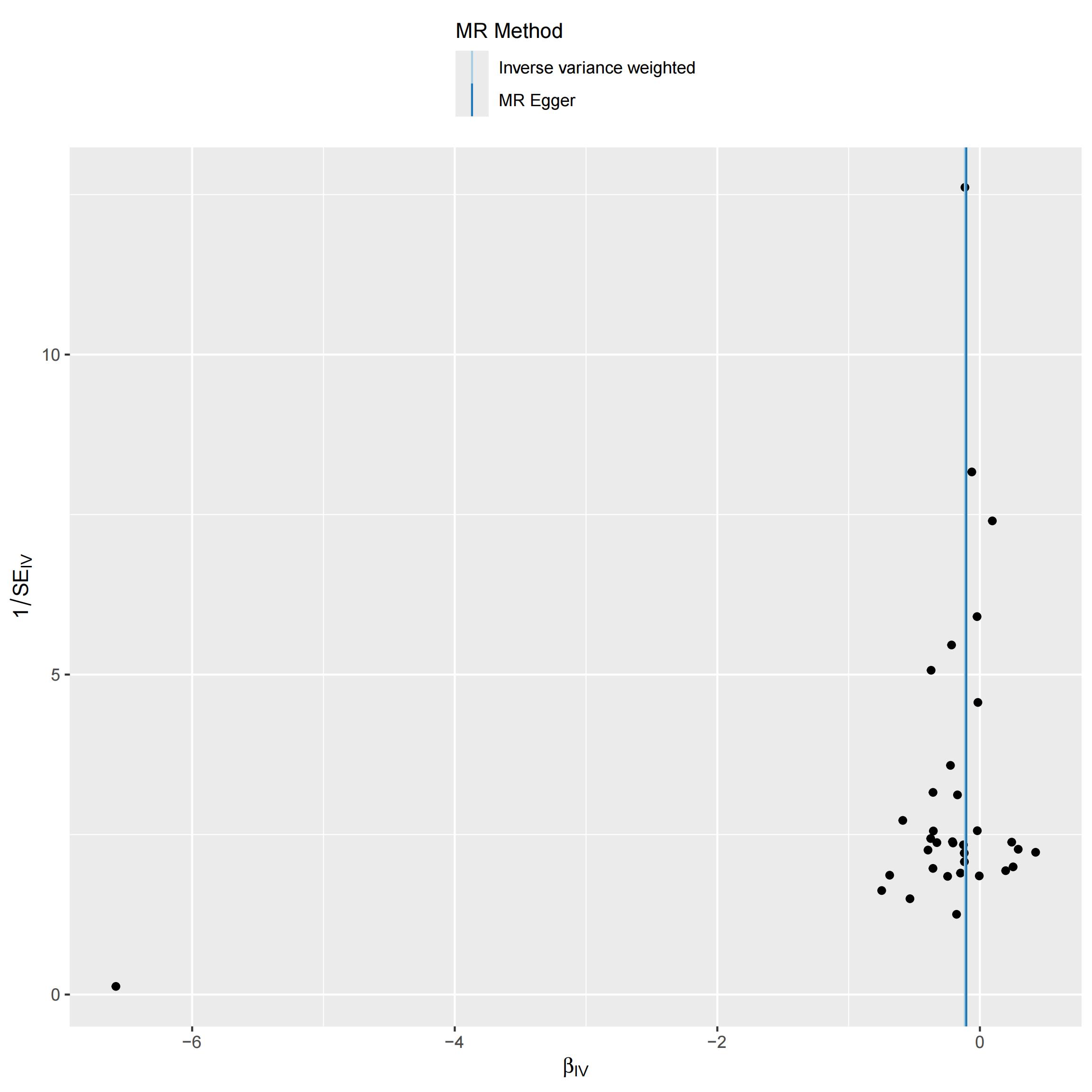  c |
| --- | --- |
| 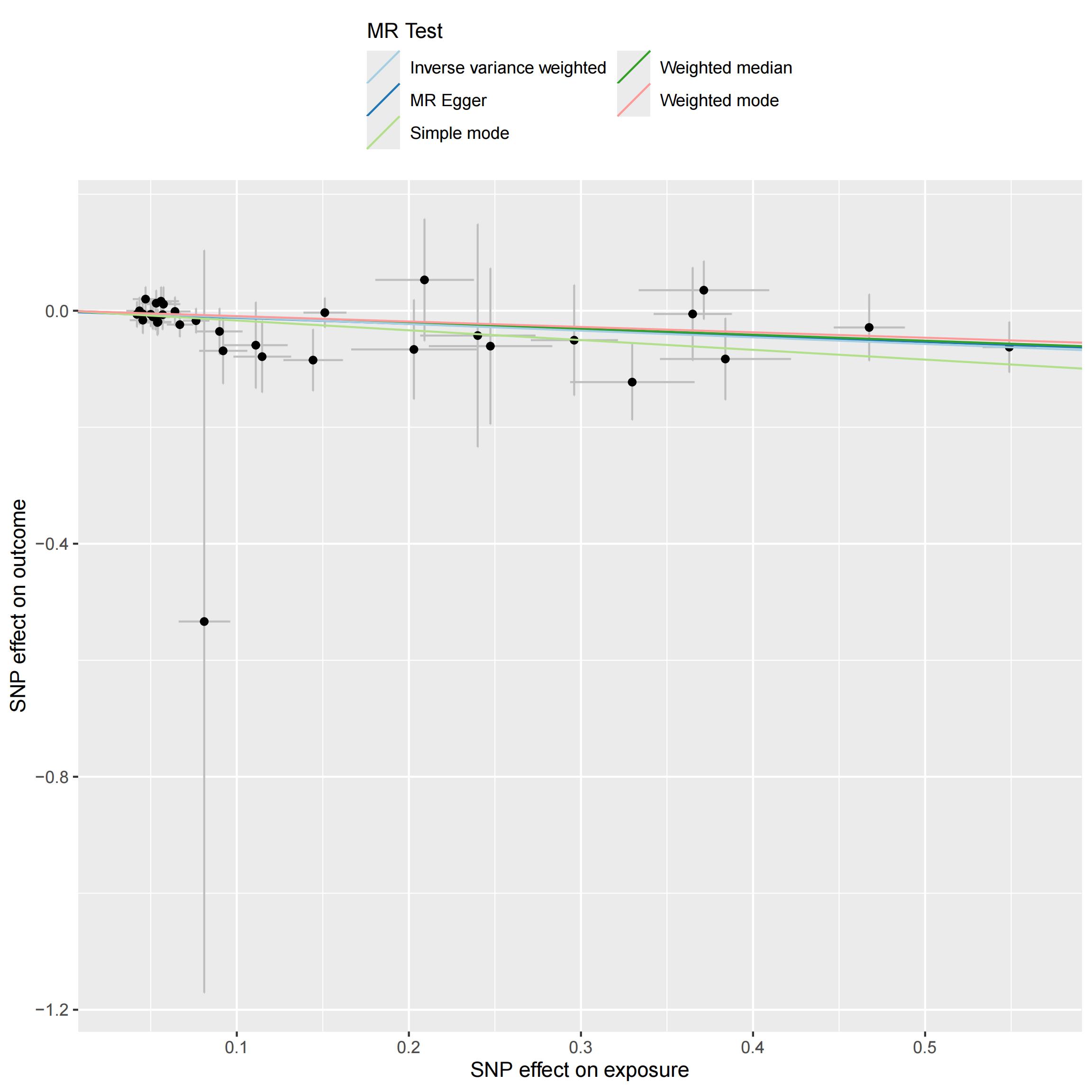  b | 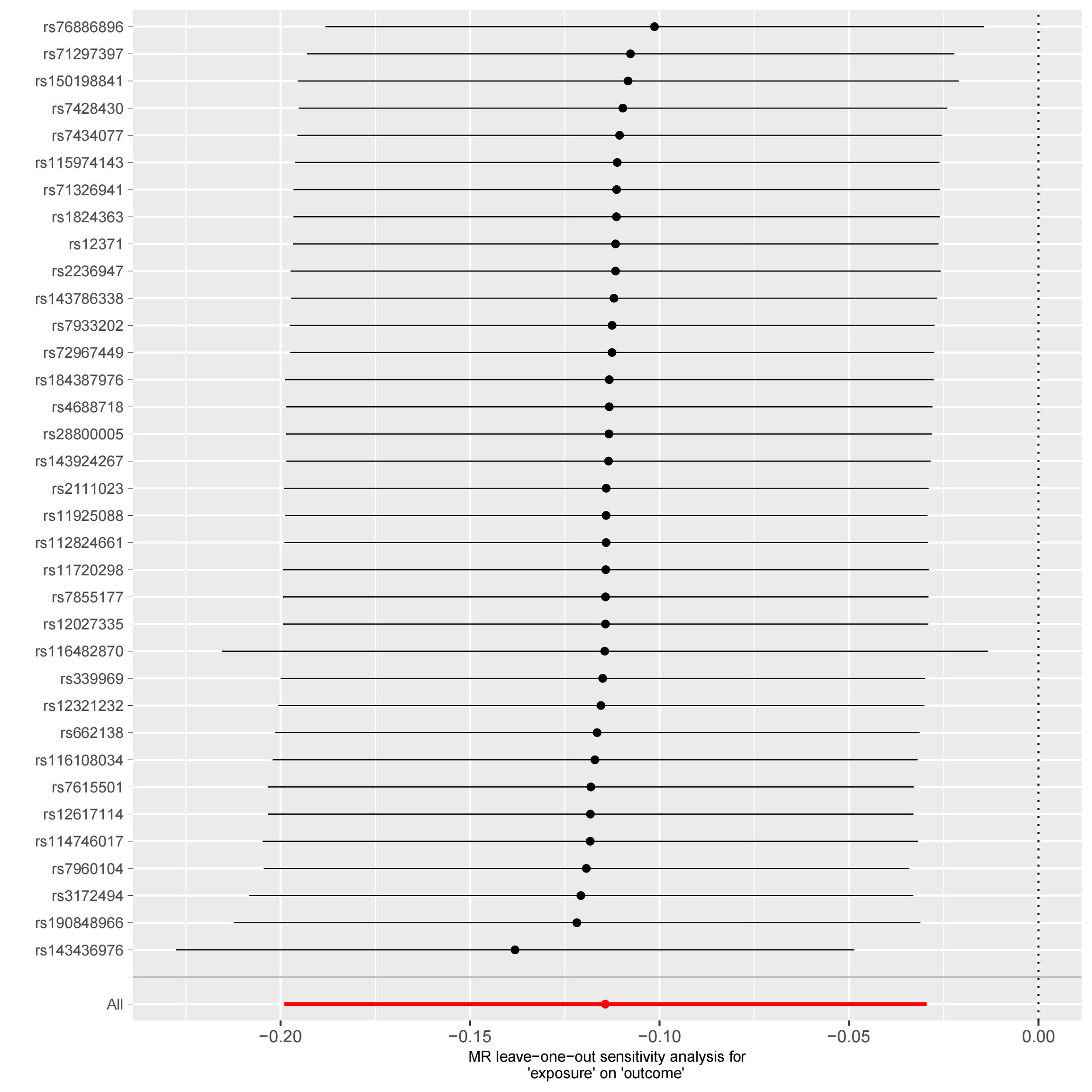  d |

Supplementary Figure S7 Forest plot (a), scatter plot(b), funnel plot (c) and sensitivity analysis (d) of SNPs associated with HYAL1 on RLS.

| 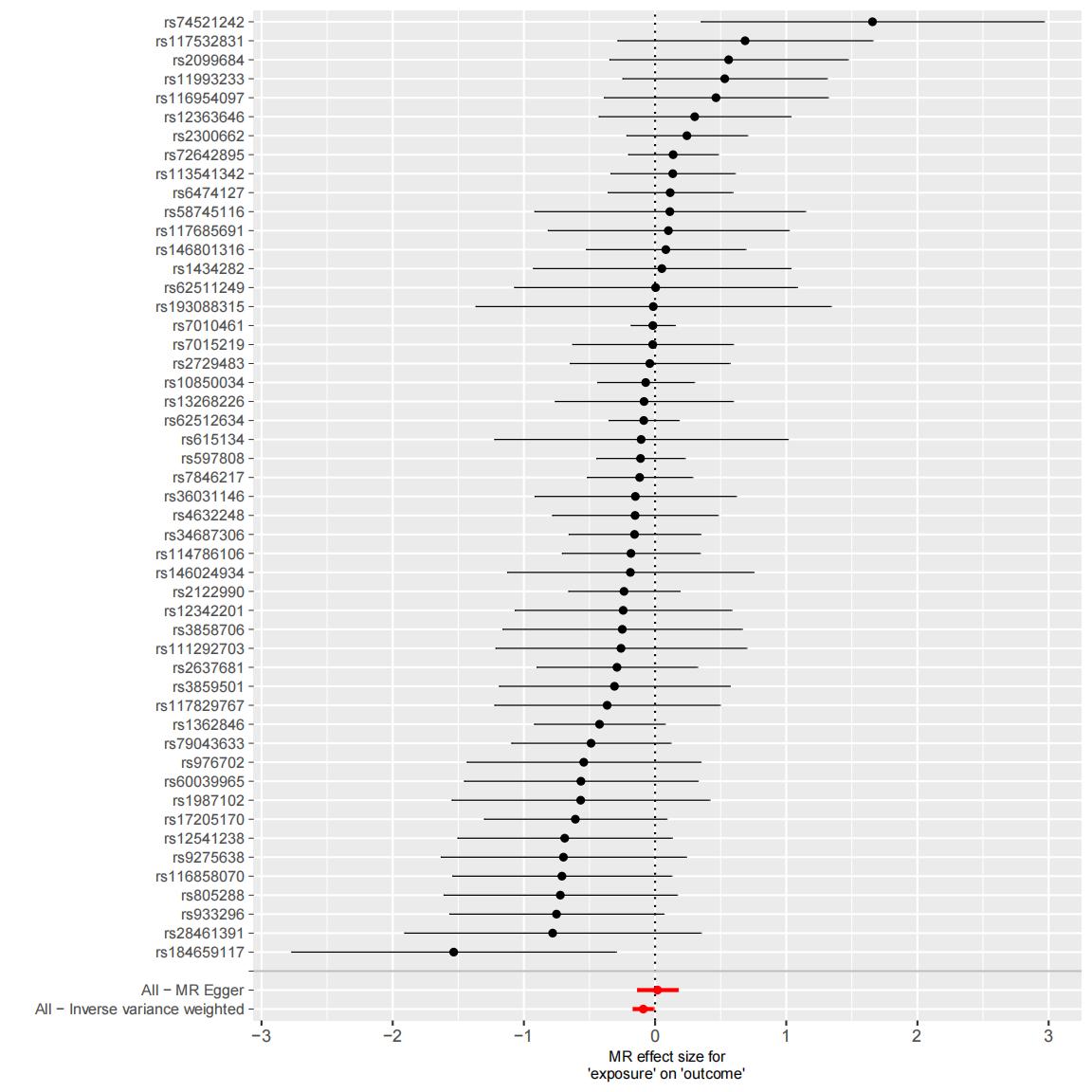  a | 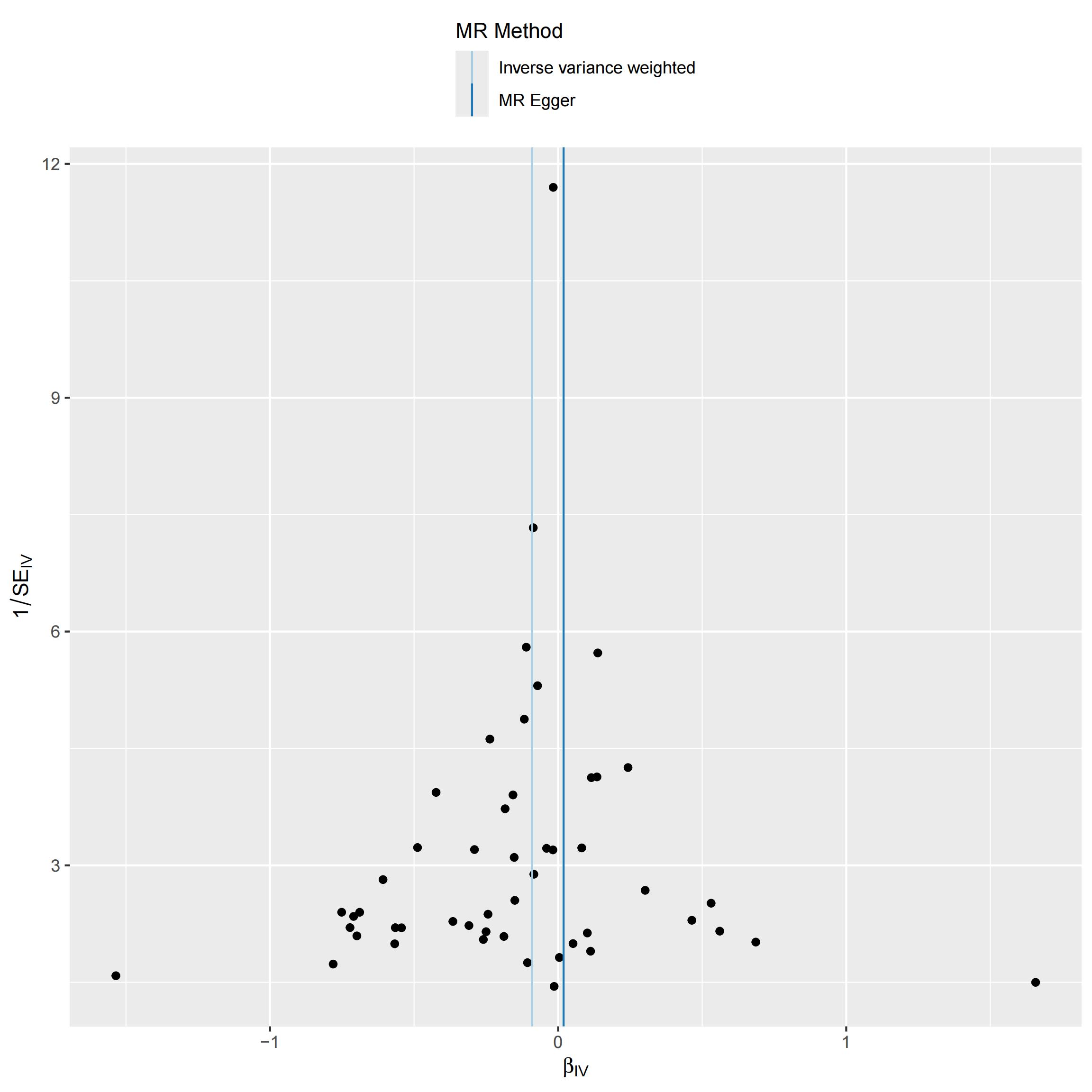  c |
| --- | --- |
| 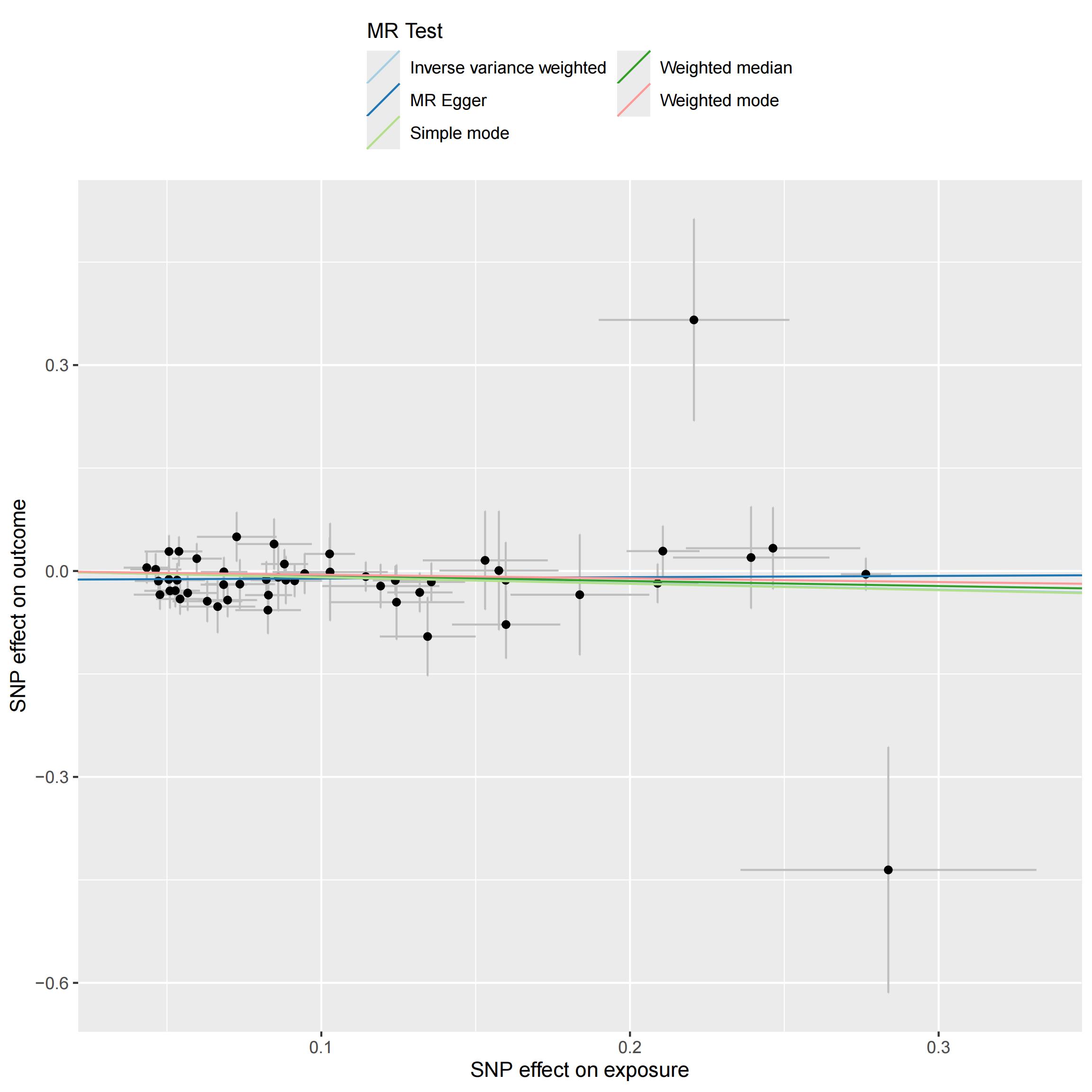  b | 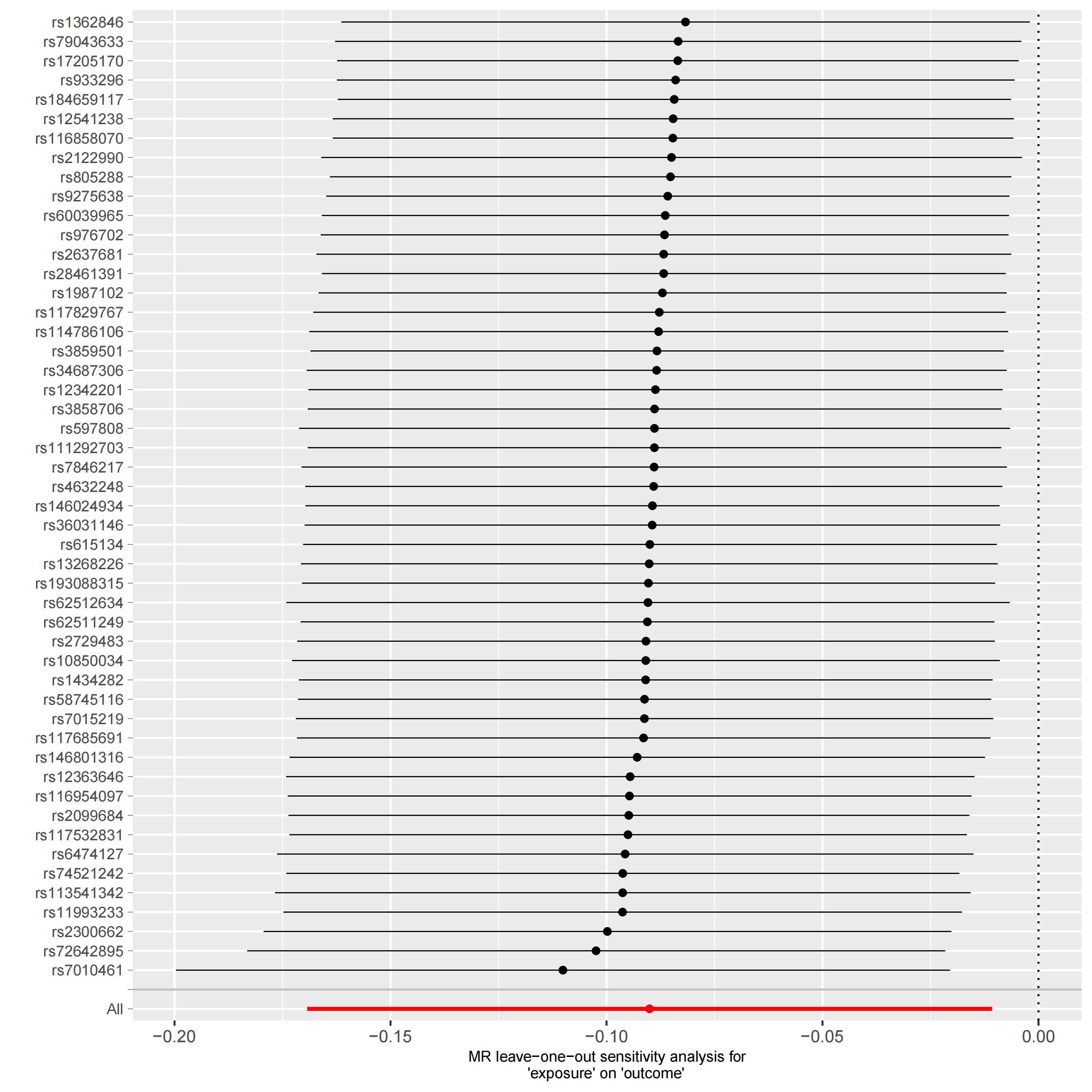  d |

Supplementary Figure S8 Forest plot (a), scatter plot(b), funnel plot (c) and sensitivity analysis (d) of SNPs associated with IDO1 on RLS.

| 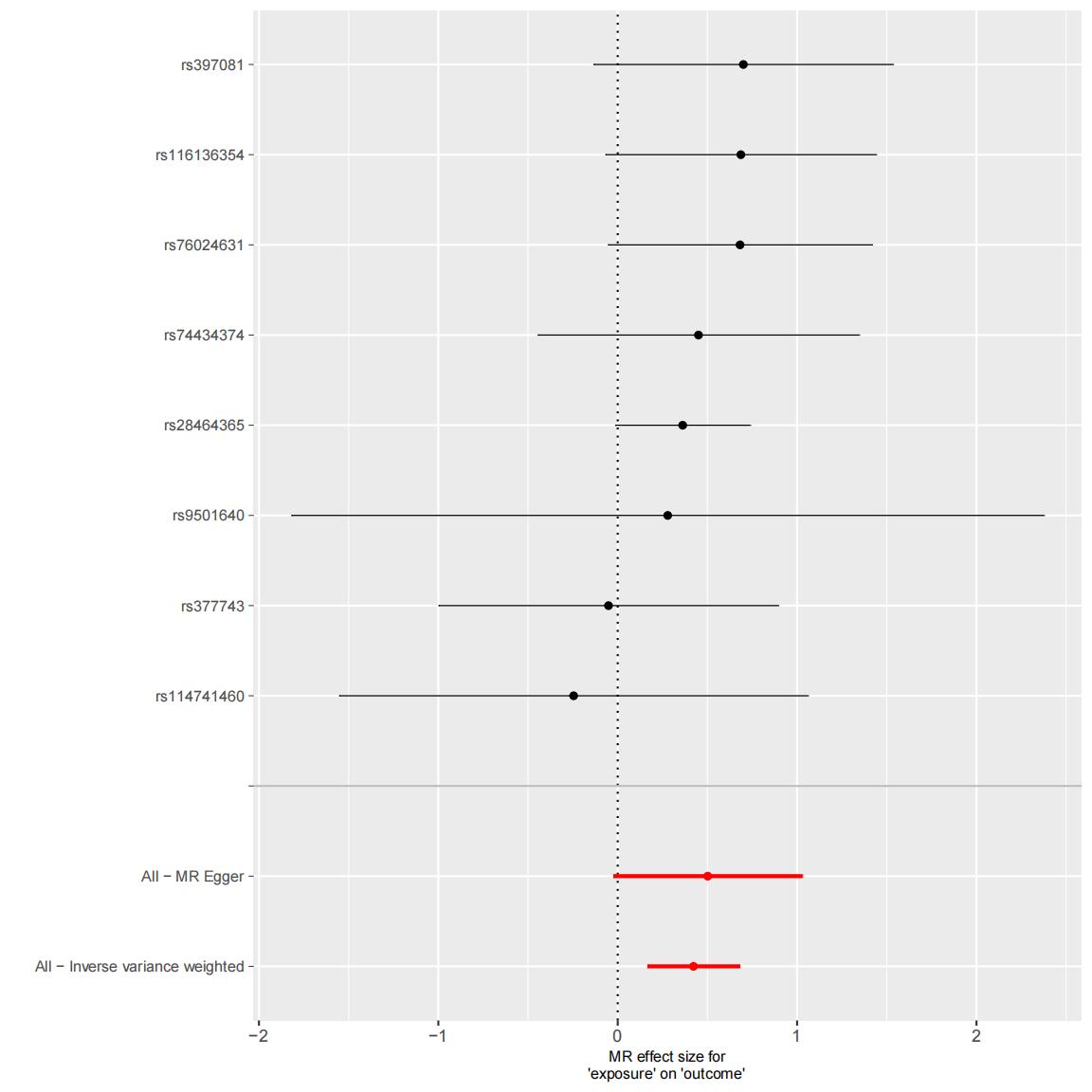  a | 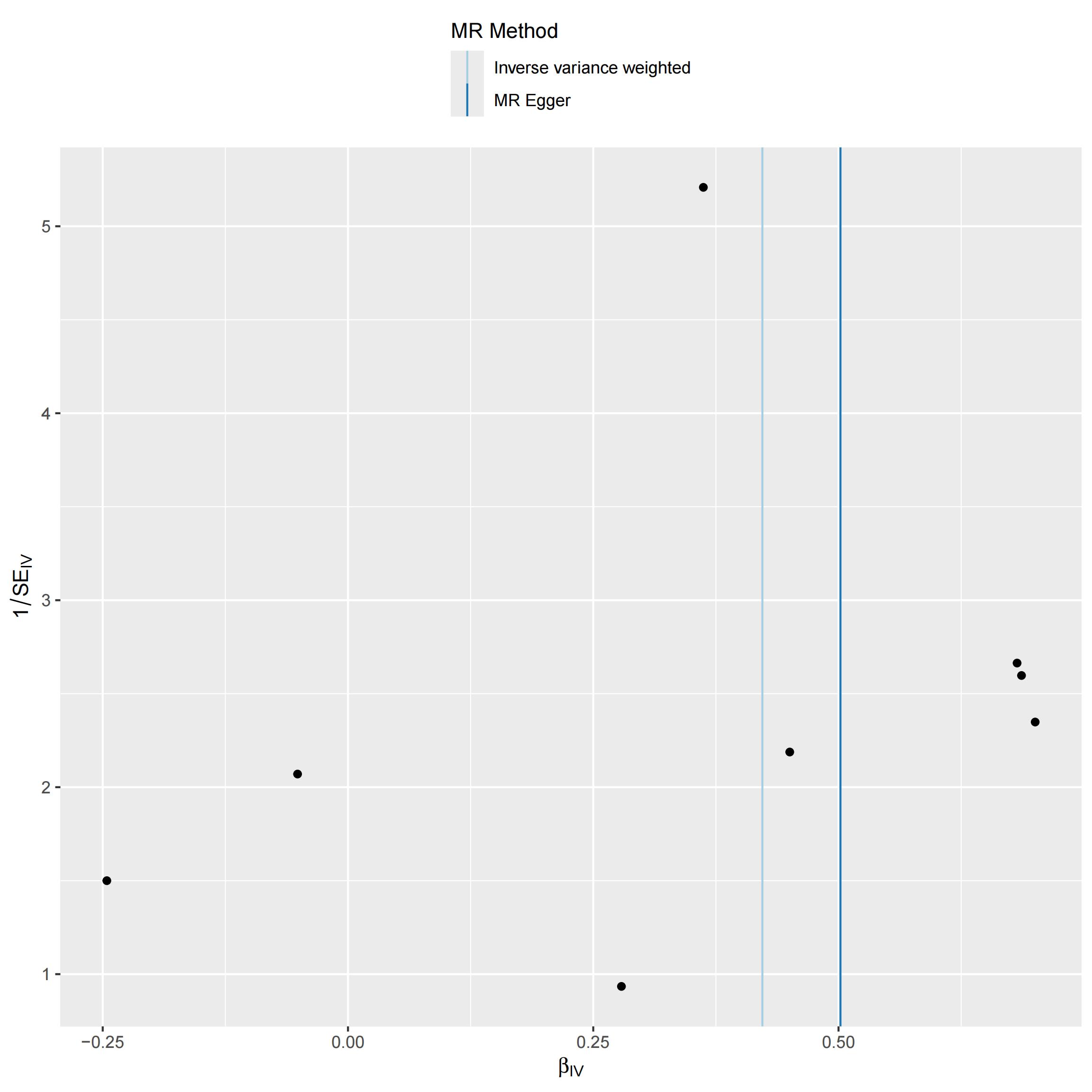  c |
| --- | --- |
| 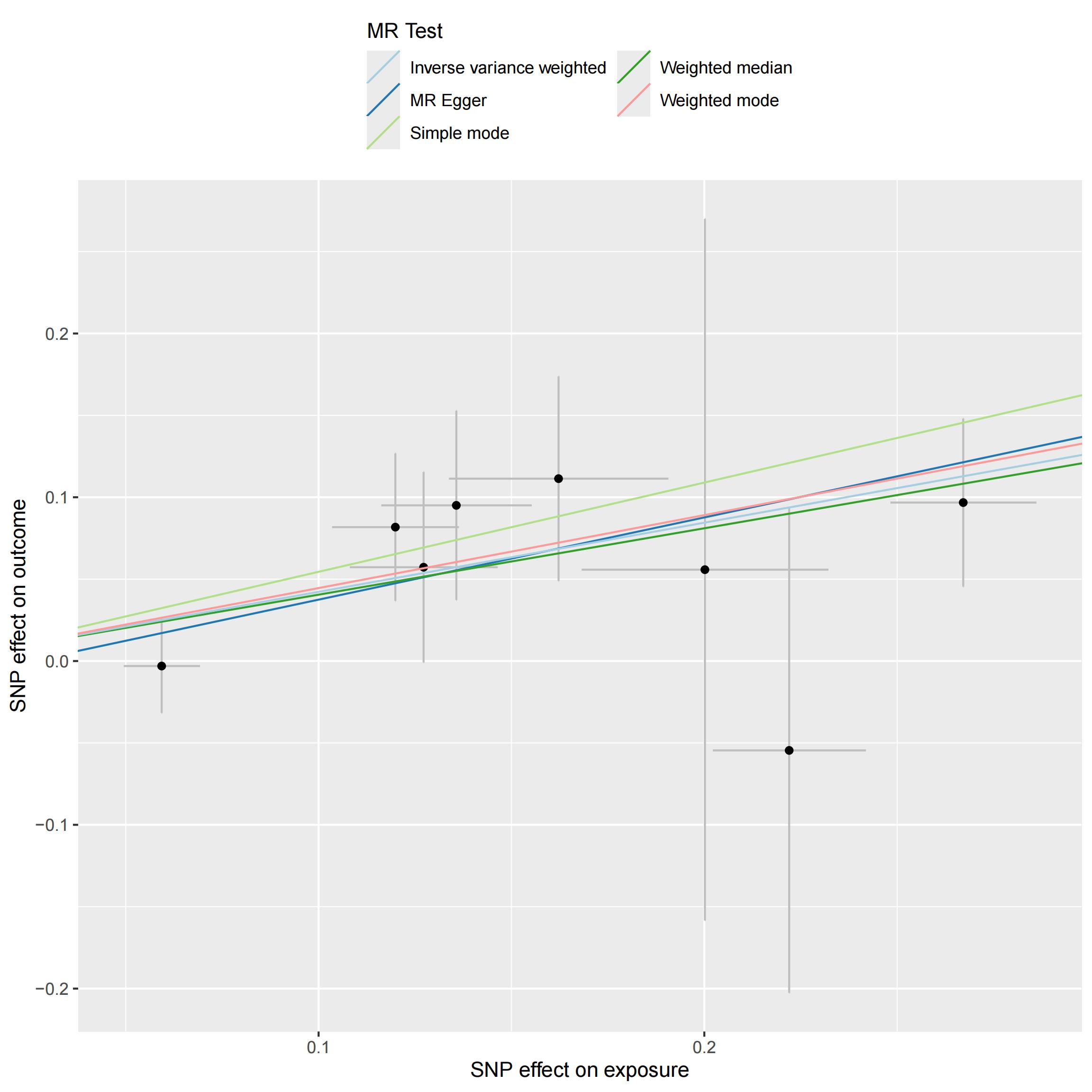  b | 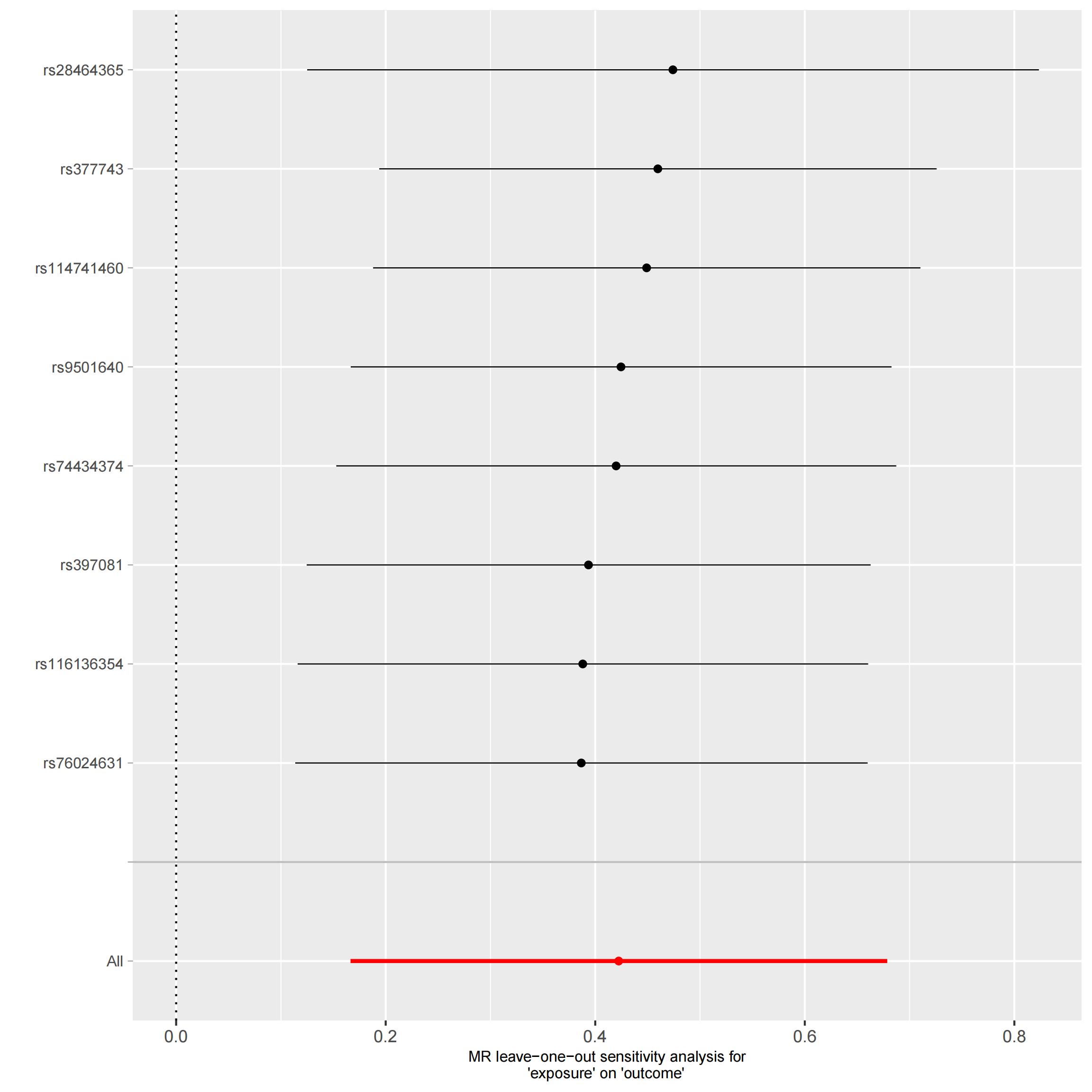  d |

Supplementary Figure S9 Forest plot (a), scatter plot(b), funnel plot (c) and sensitivity analysis (d) of SNPs associated with IL1A on RLS.

| 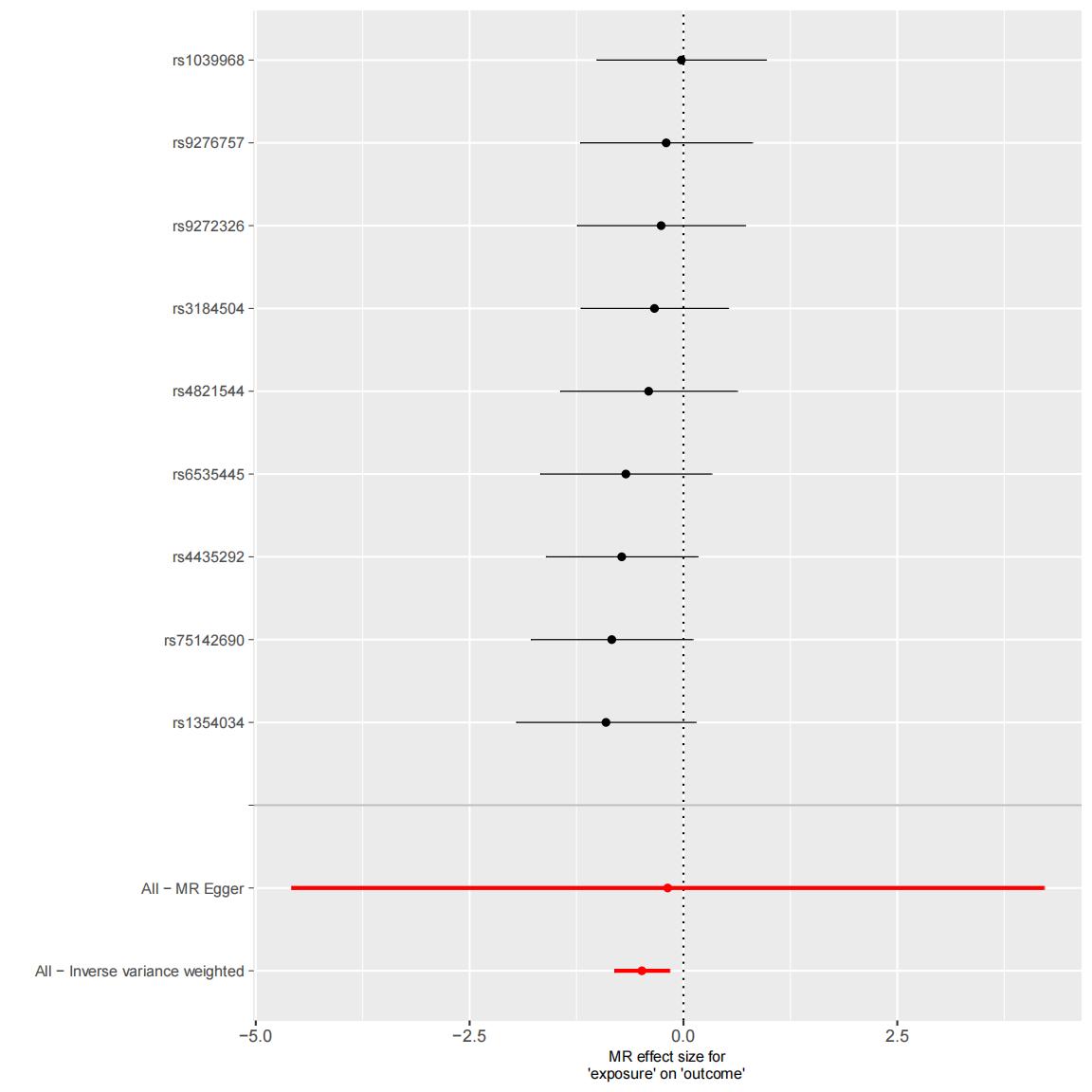  a | 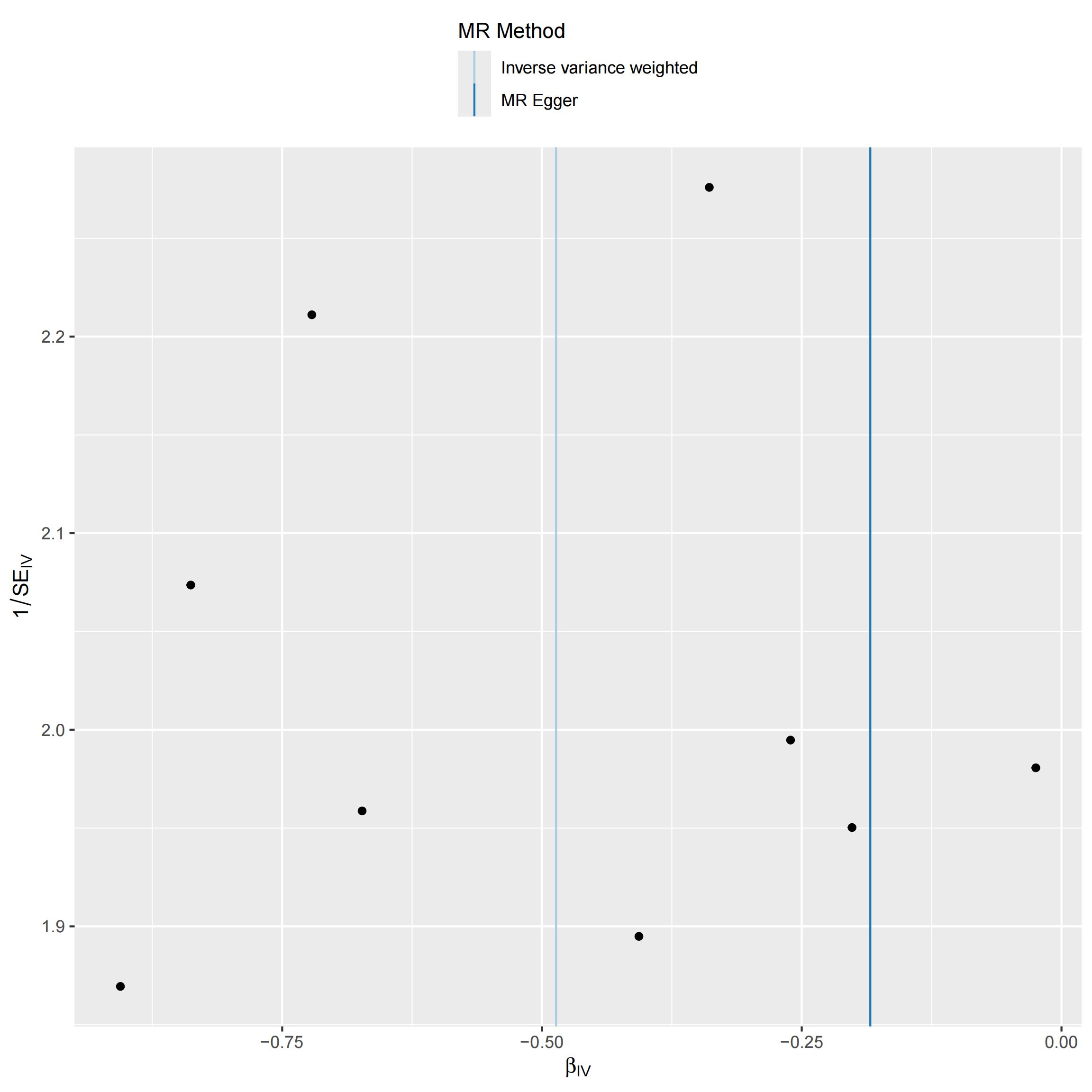  c |
| --- | --- |
| 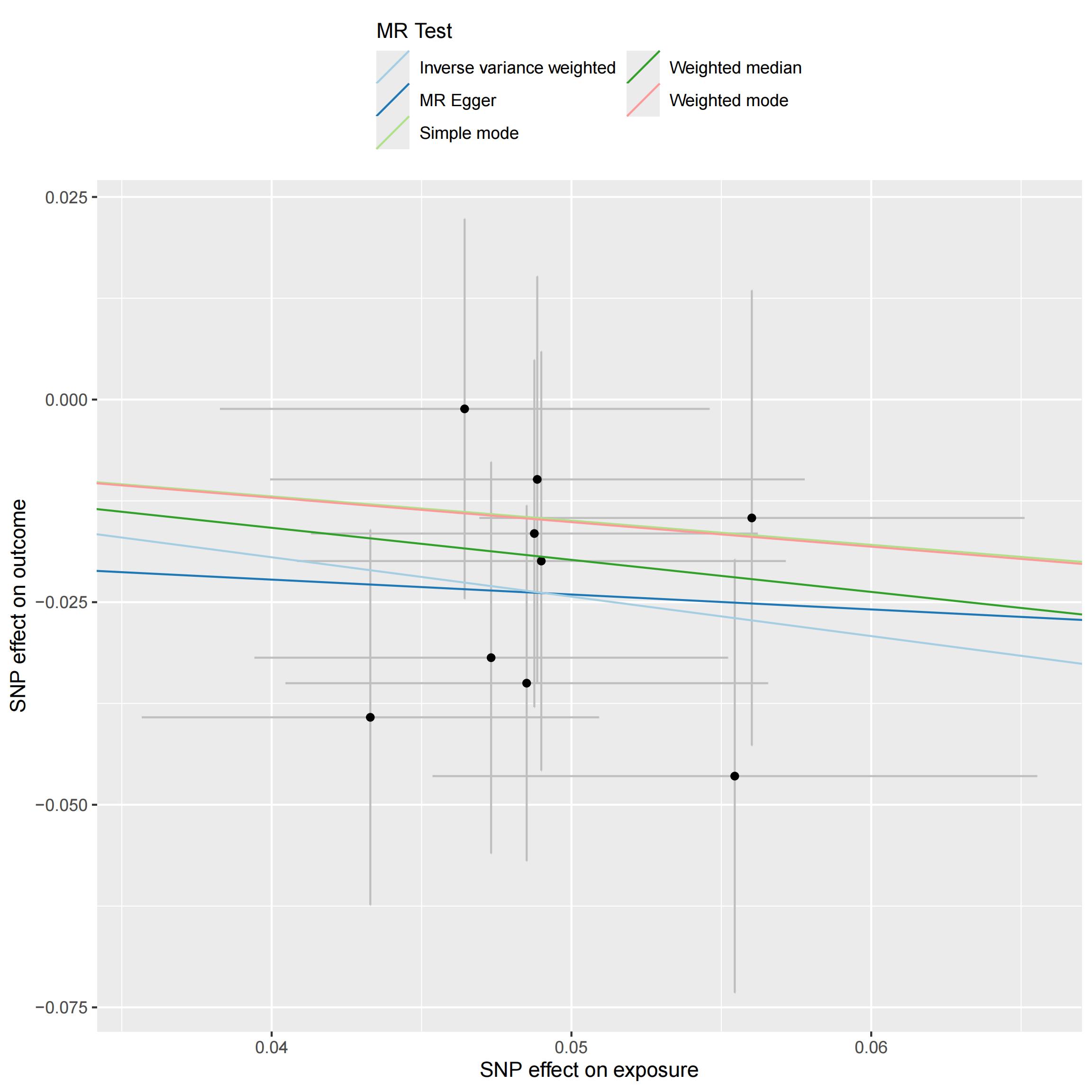  b | 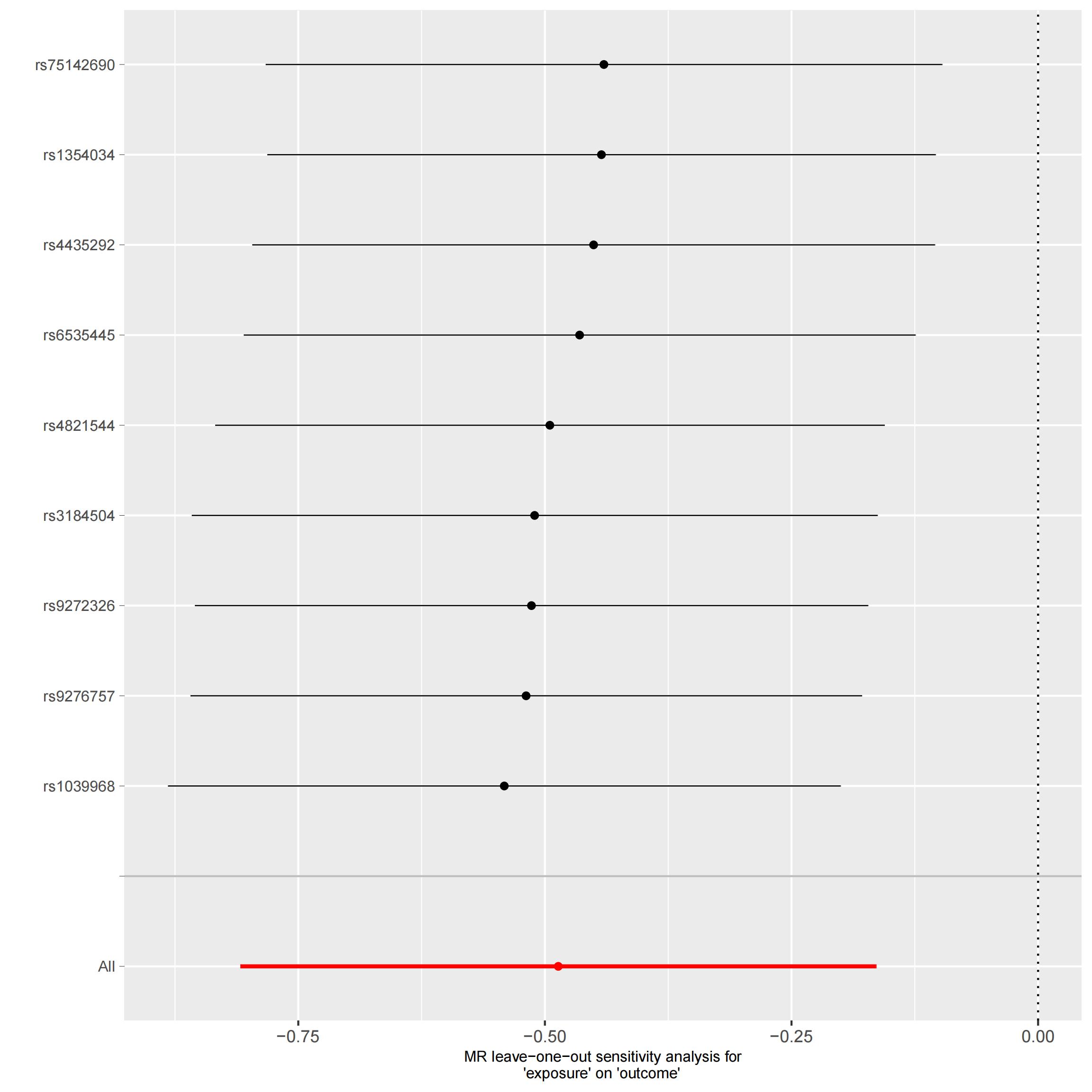  d |

Supplementary Figure S10 Forest plot (a), scatter plot(b), funnel plot (c) and sensitivity analysis (d) of SNPs associated with KCTD5 on RLS.

| 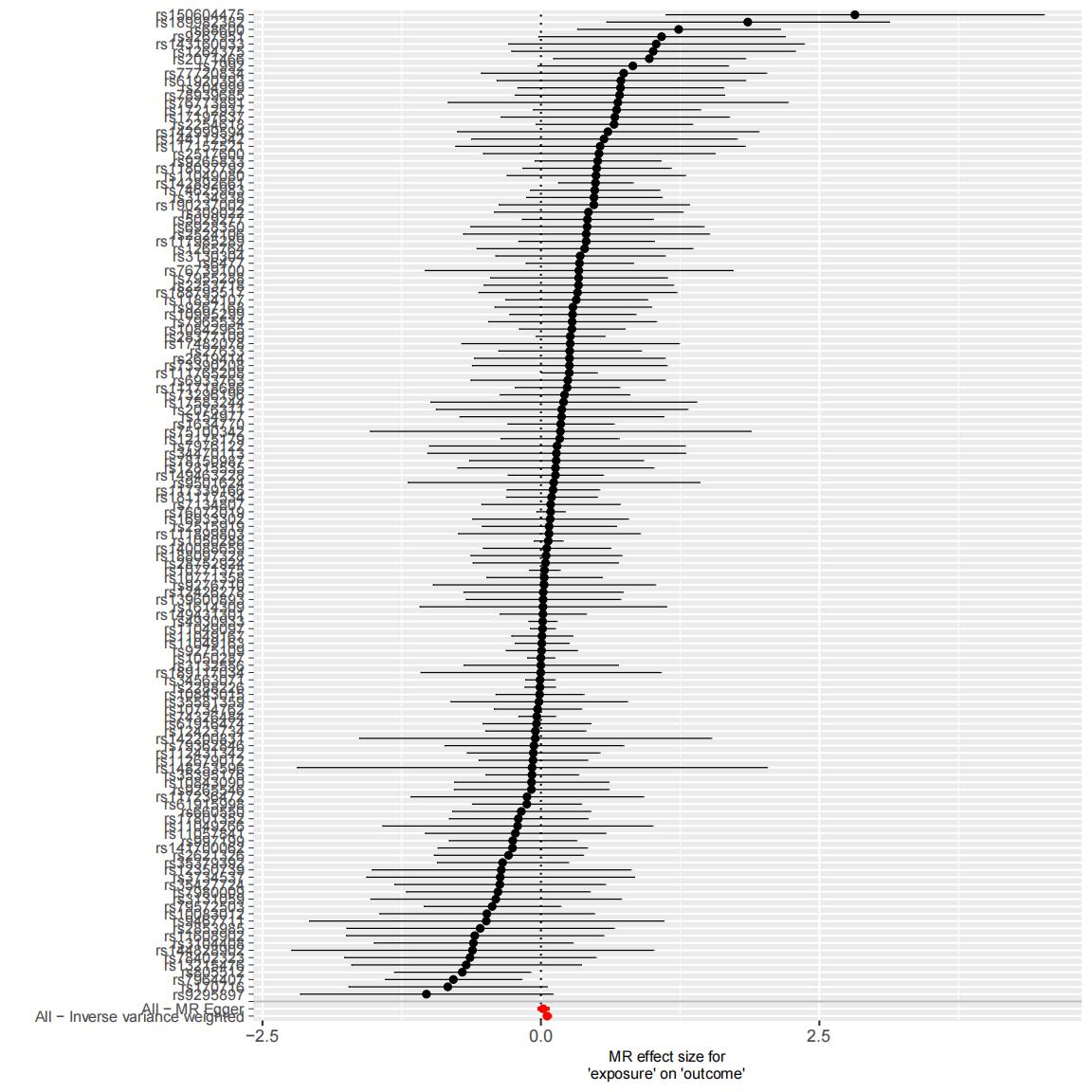  a | 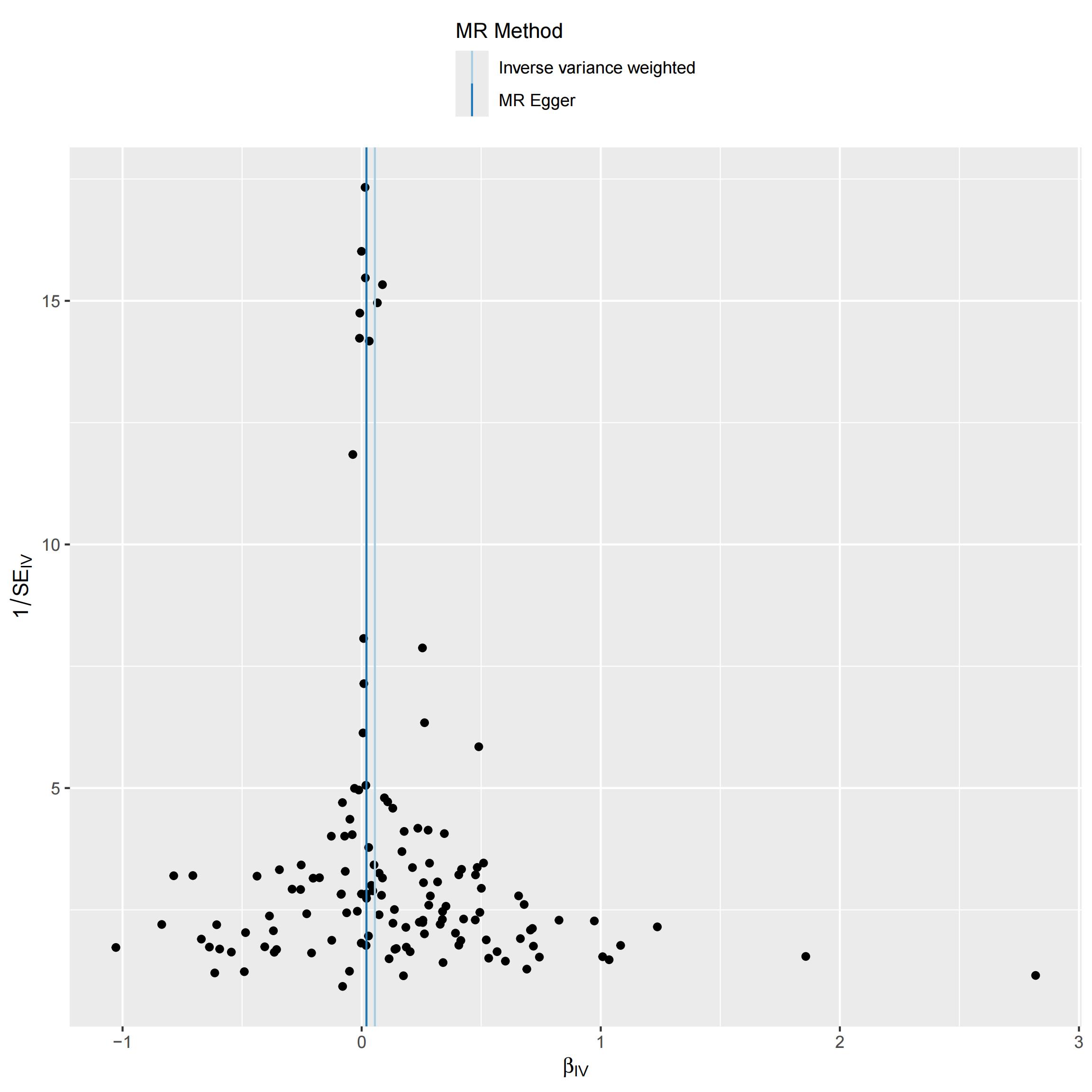  c |
| --- | --- |
| 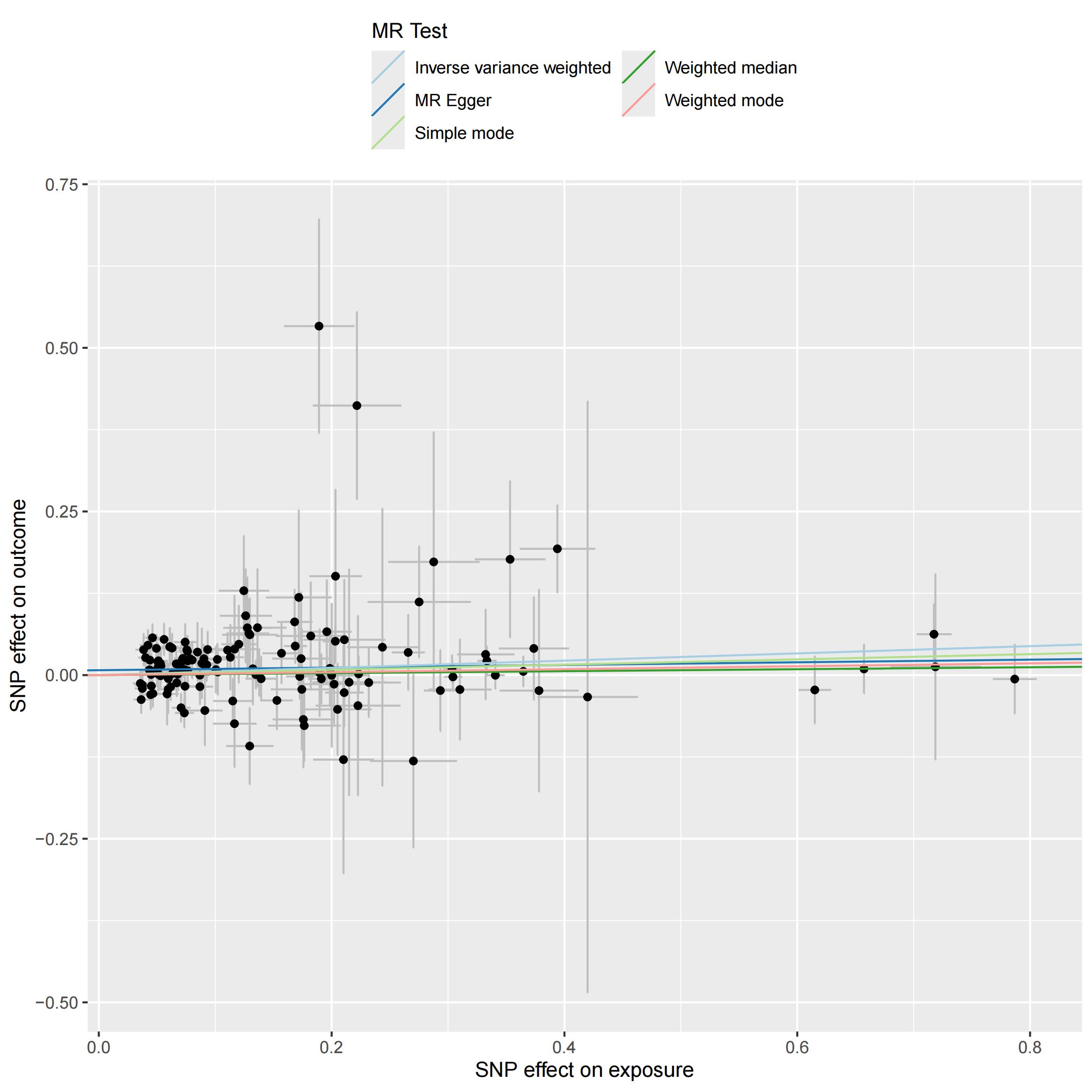  b | 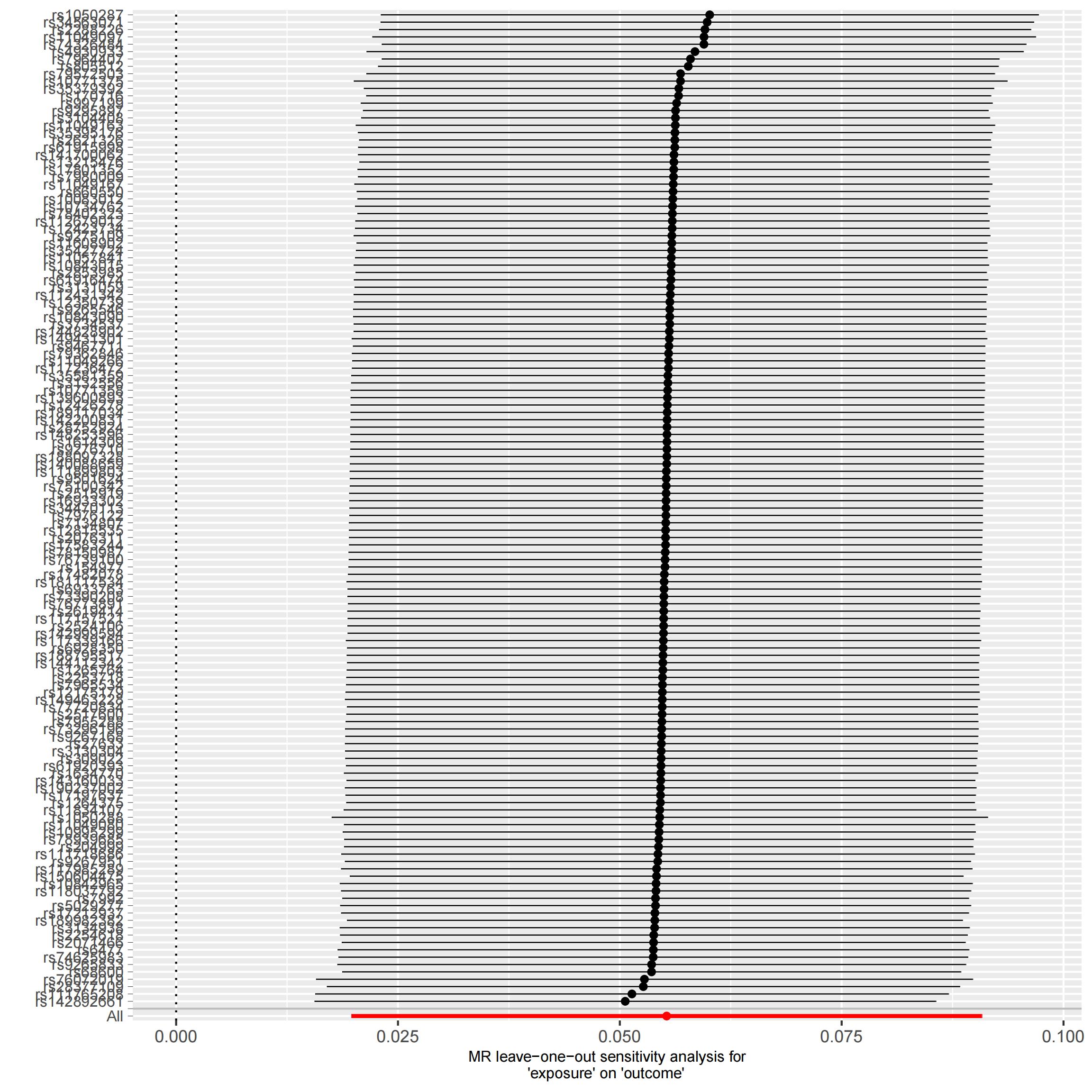  d |

Supplementary Figure S11 Forest plot (a), scatter plot(b), funnel plot (c) and sensitivity analysis (d) of SNPs associated with MANSC4 on RLS.

| 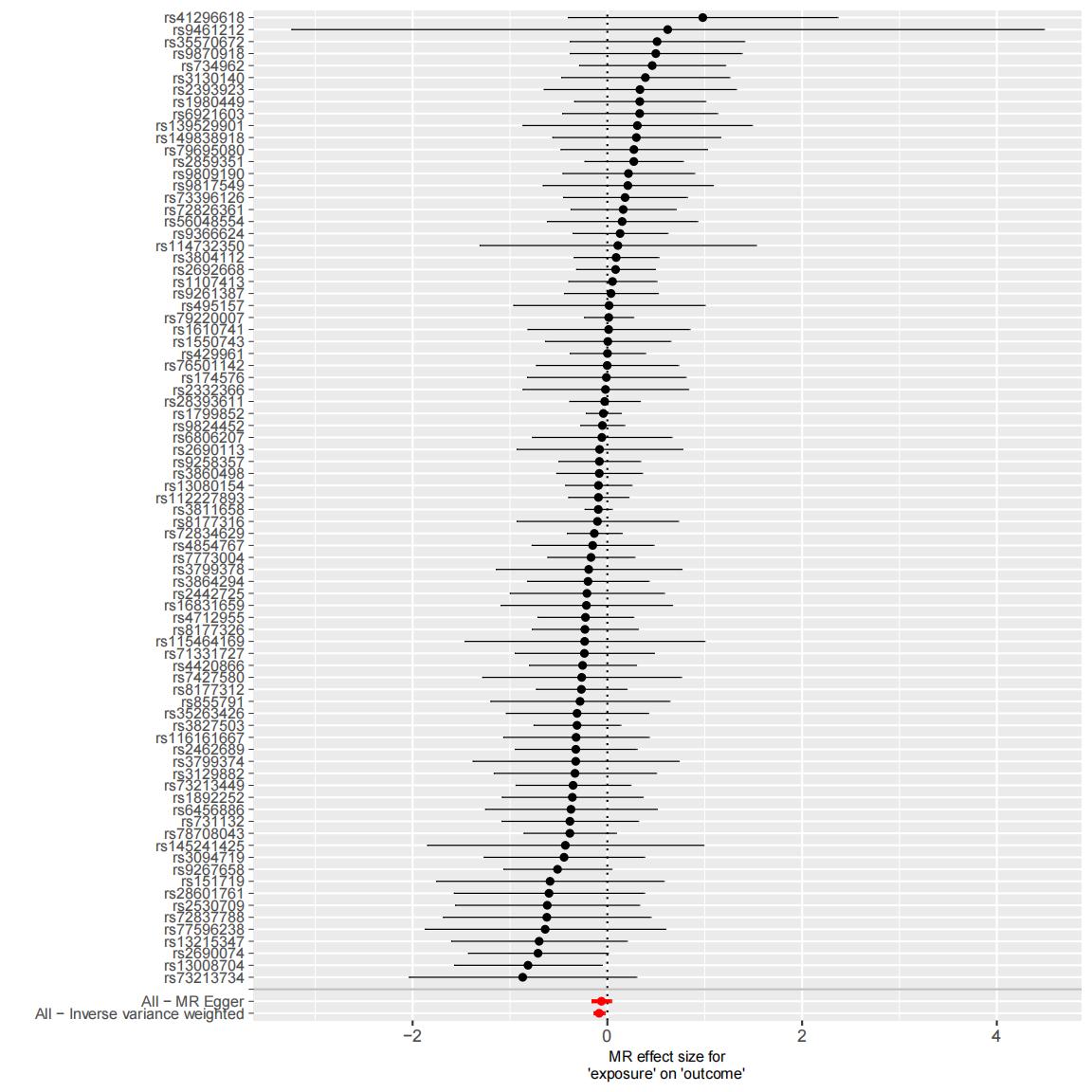  a | 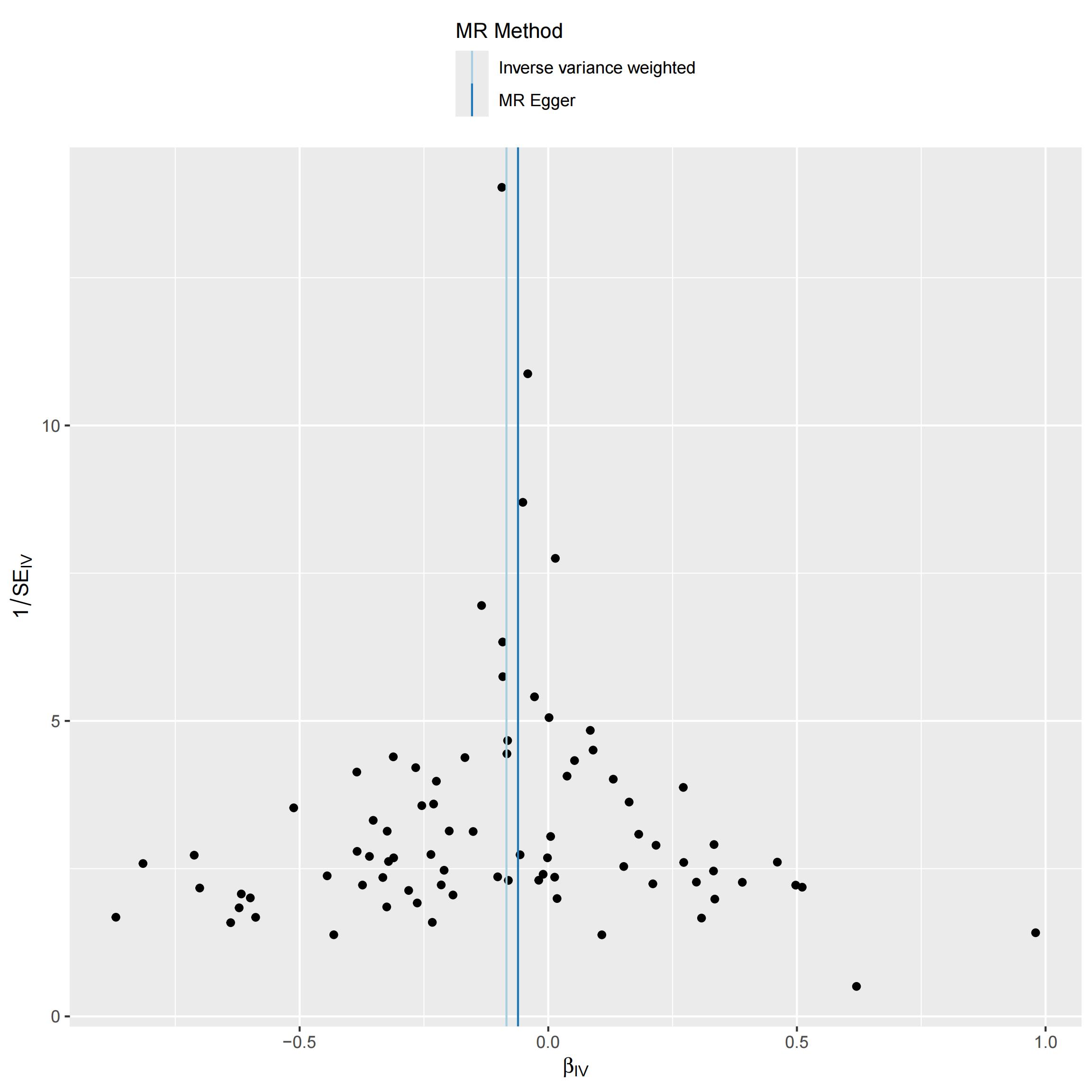  c |
| --- | --- |
| 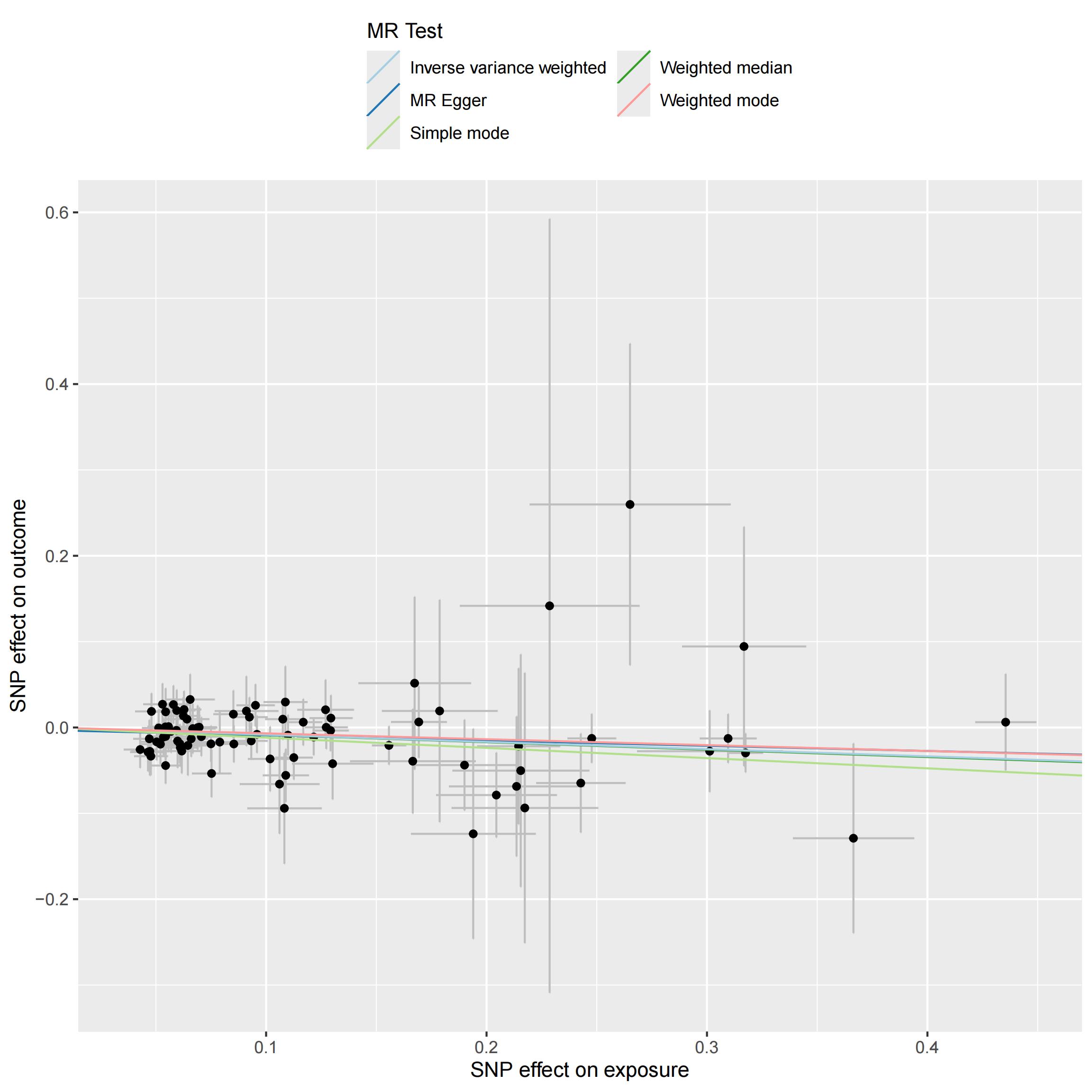  b | 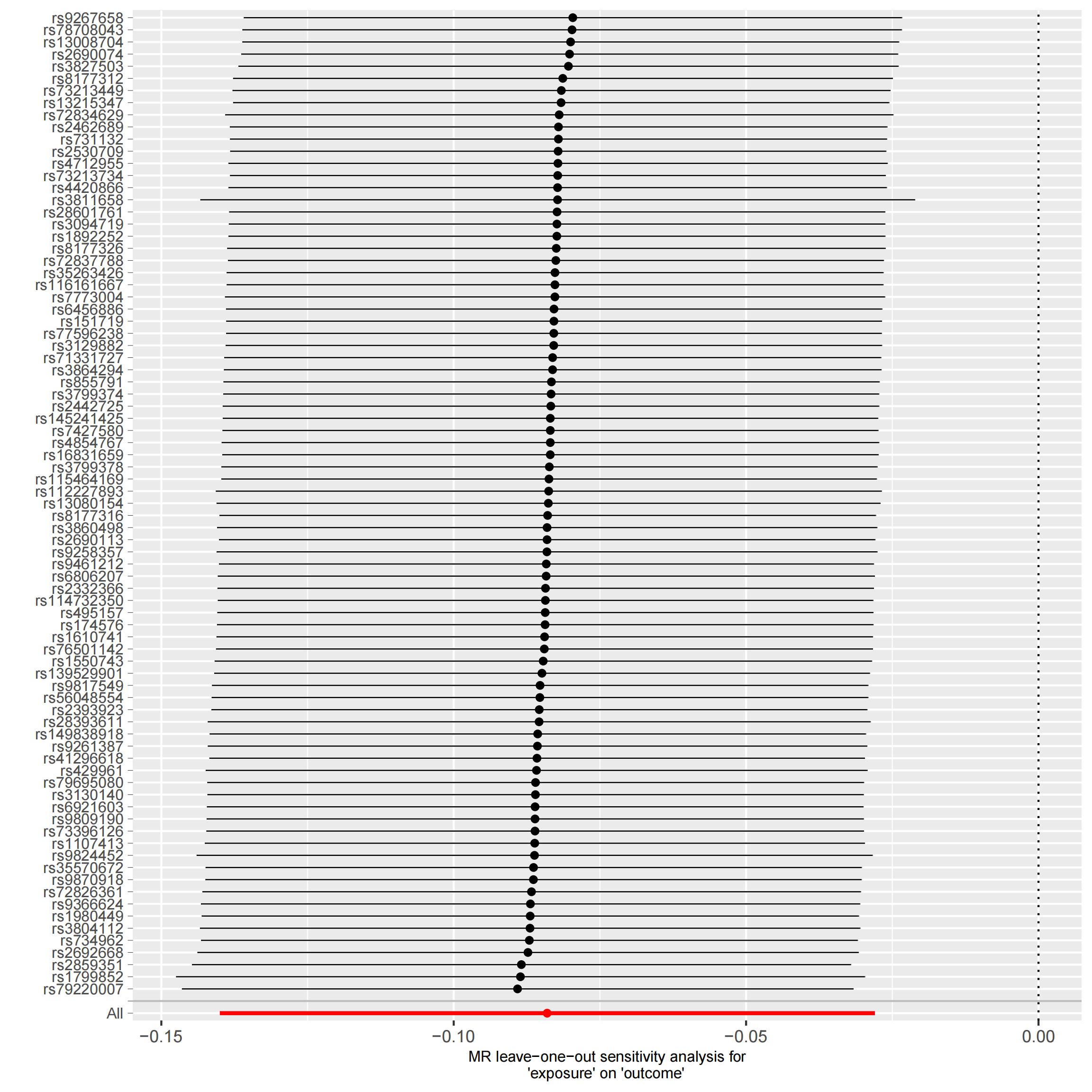  d |

Supplementary Figure S12 Forest plot (a), scatter plot(b), funnel plot (c) and sensitivity analysis (d) of SNPs associated with TF on RLS.

| 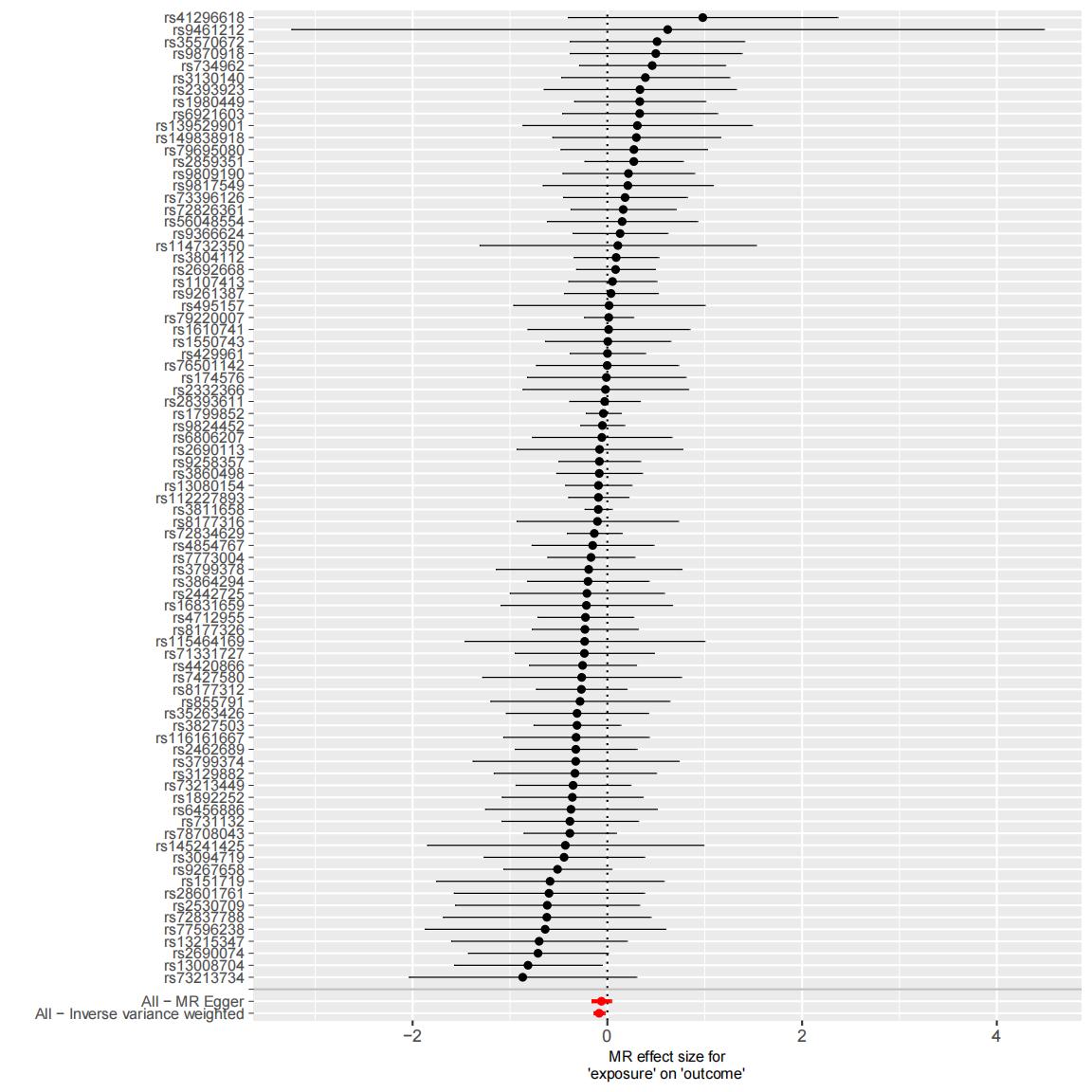  a | 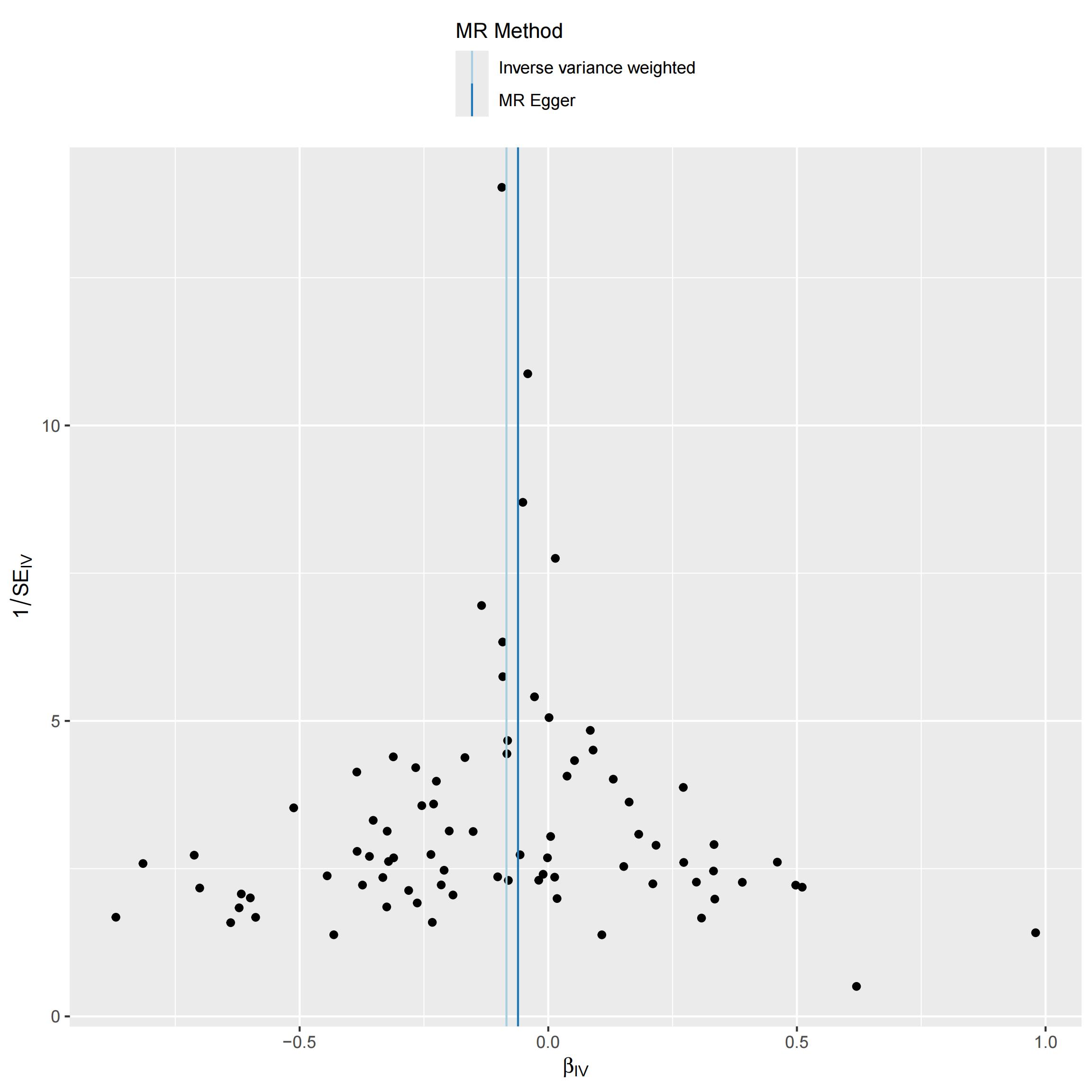  c |
| --- | --- |
| 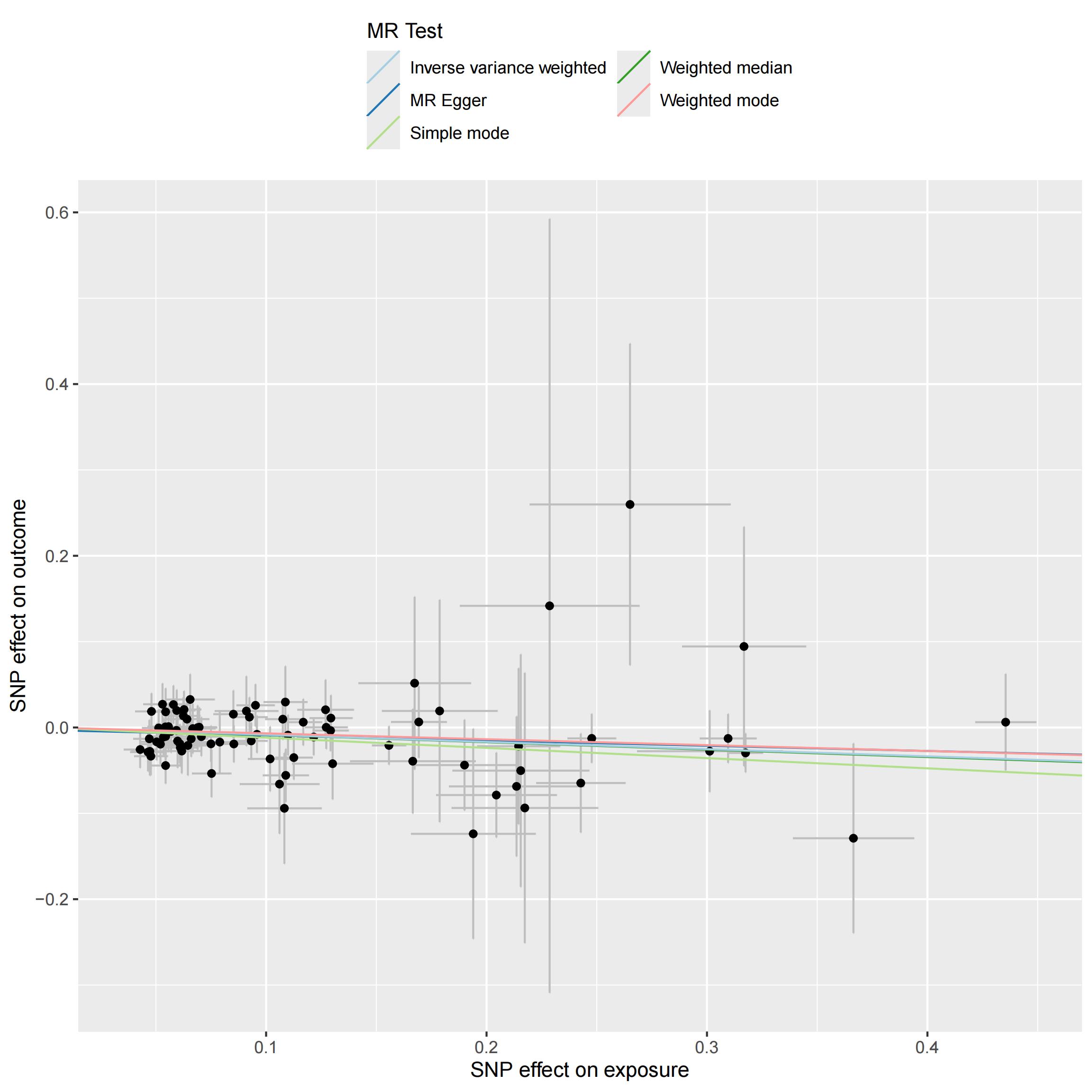  b | 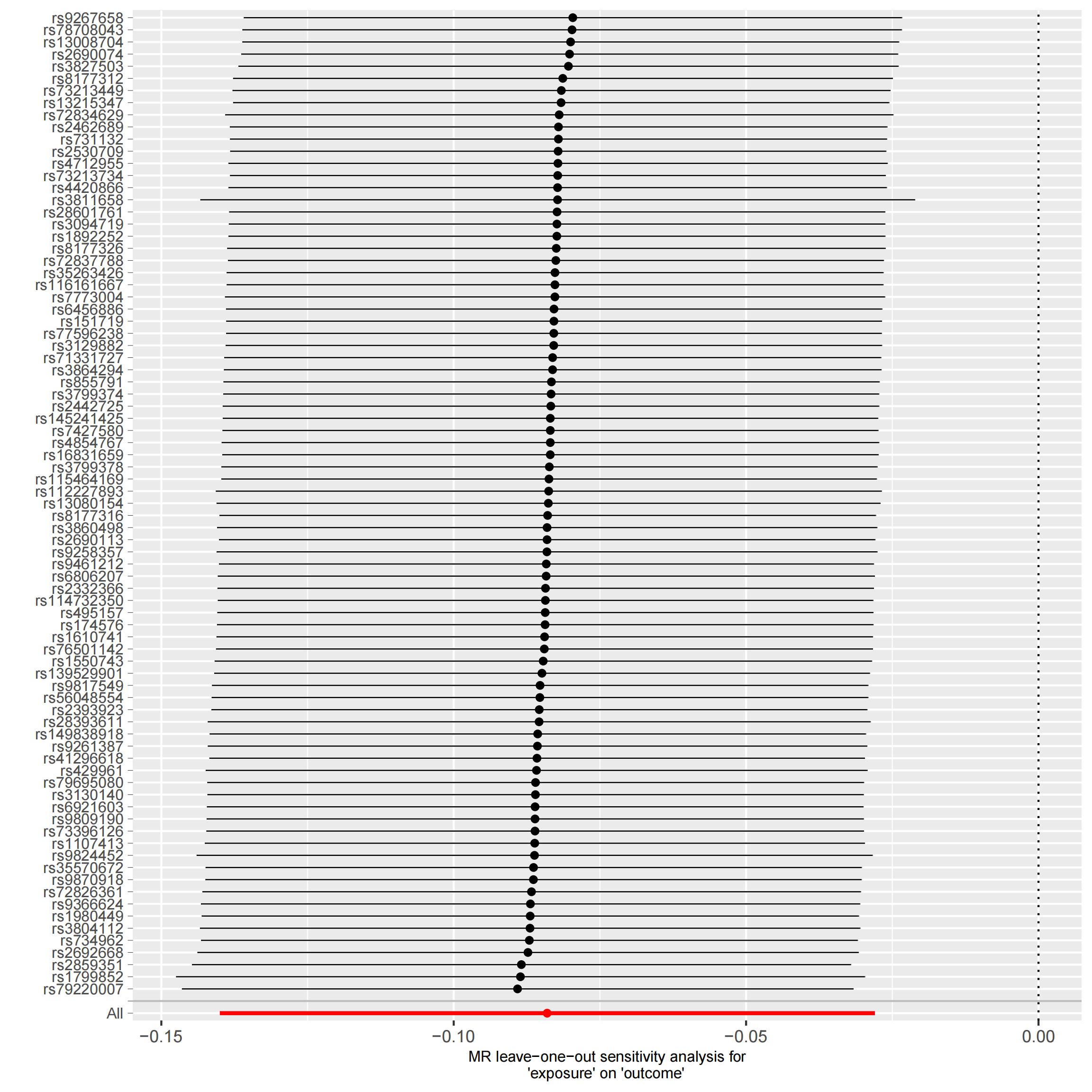  d |

Supplementary Figure S13 Forest plot (a), scatter plot(b), funnel plot (c) and sensitivity analysis (d) of SNPs associated with TLR1 on RLS.

**MR Analysis of the Relationship Between upstream genes and ferroptosis**

| 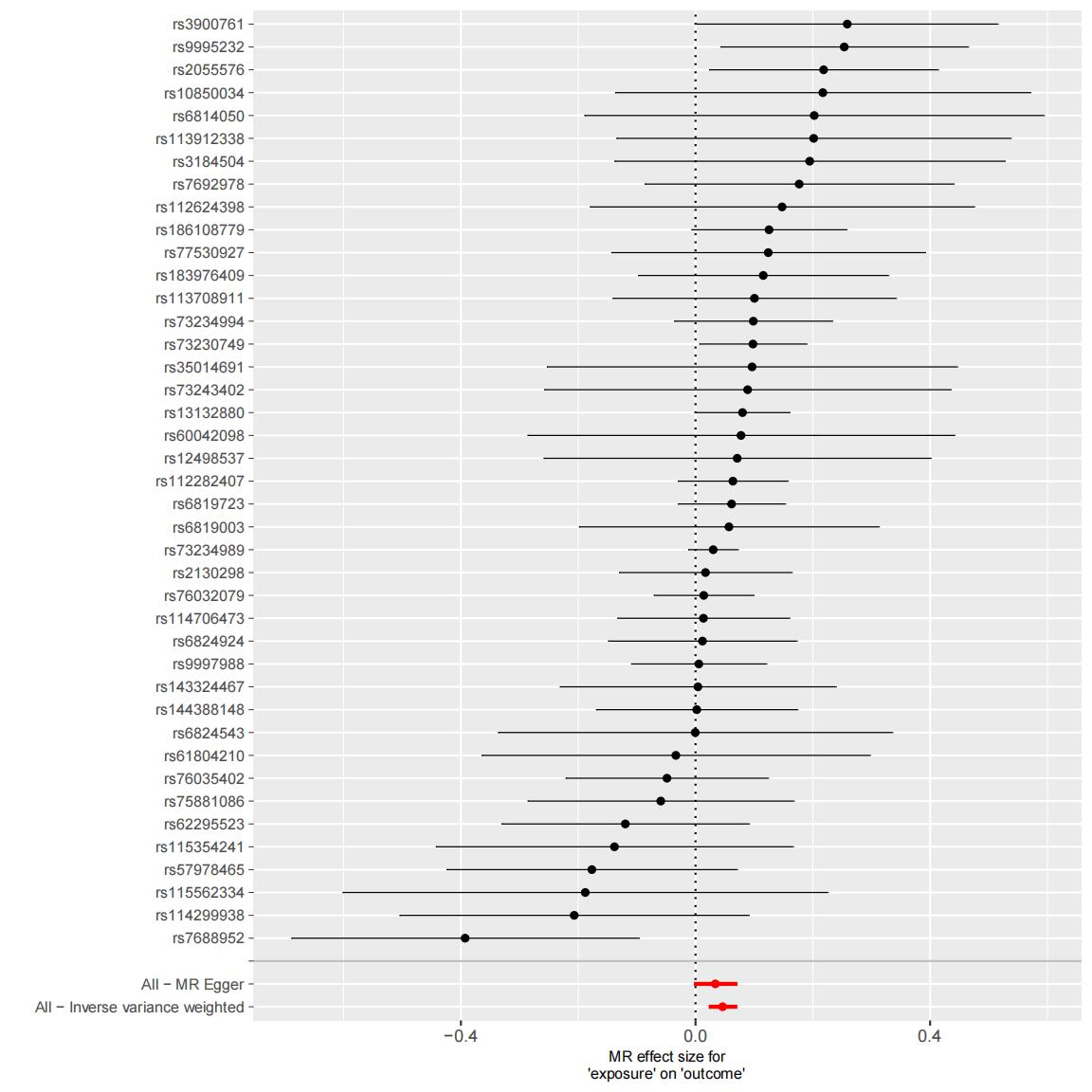  a | 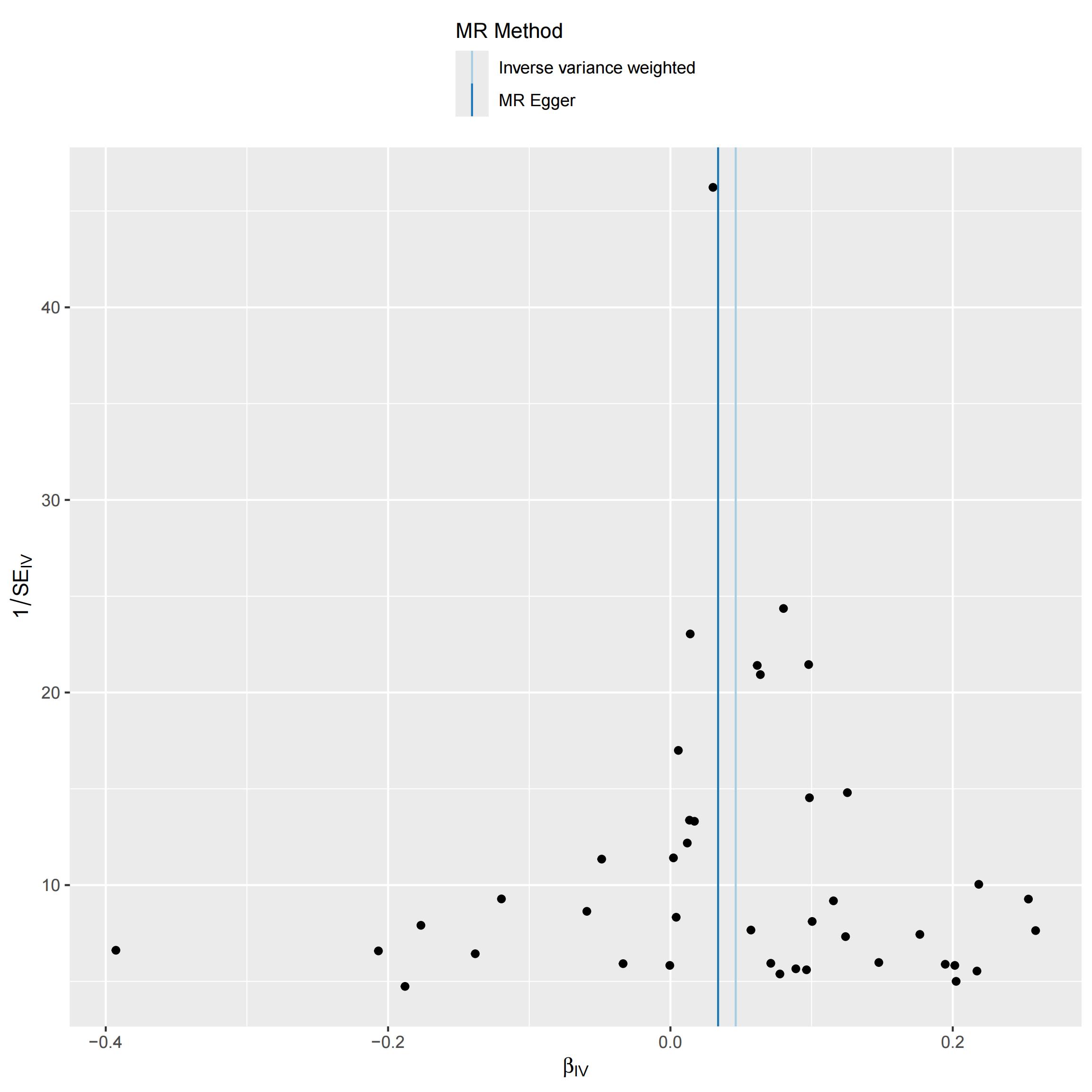  c |
| --- | --- |
| 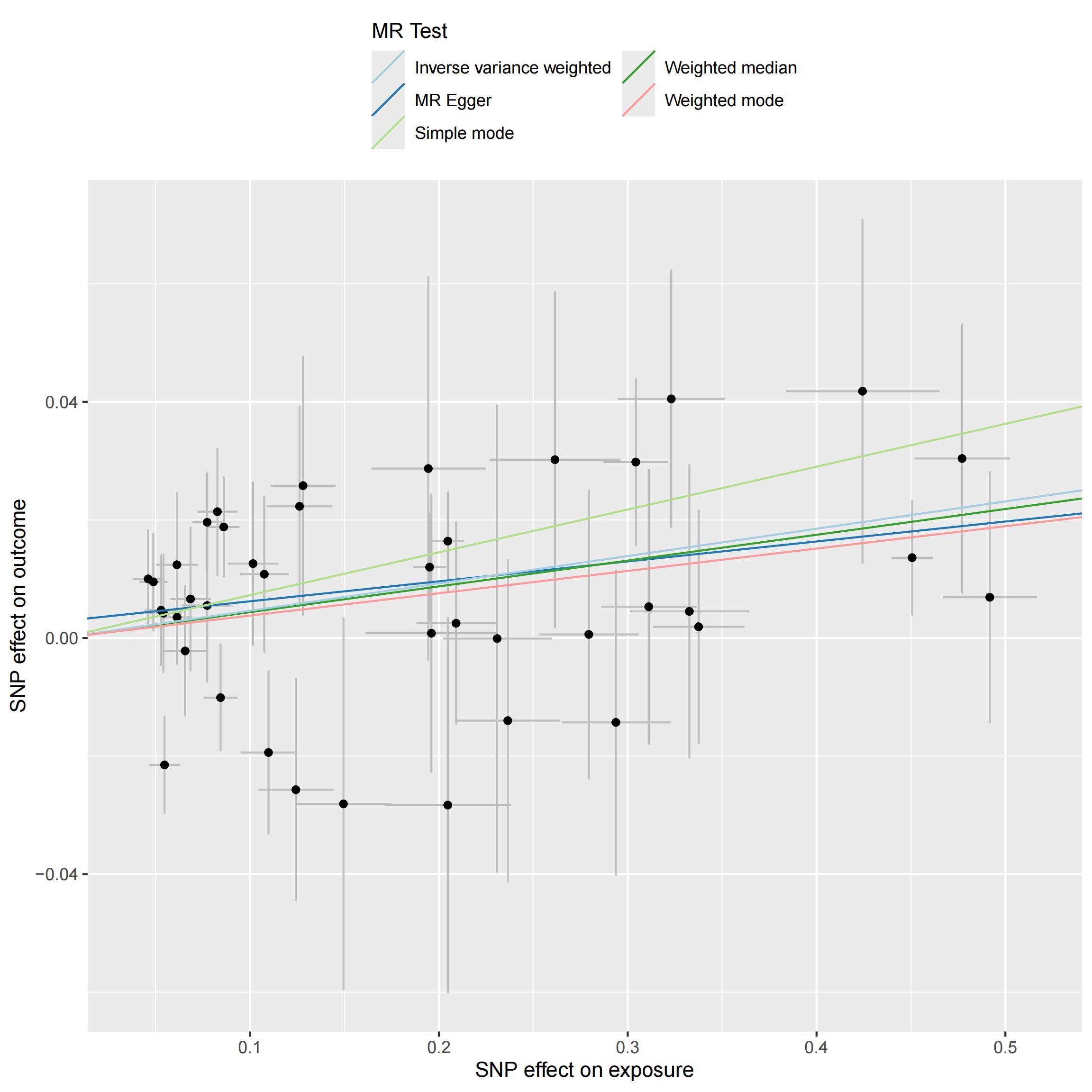  b | 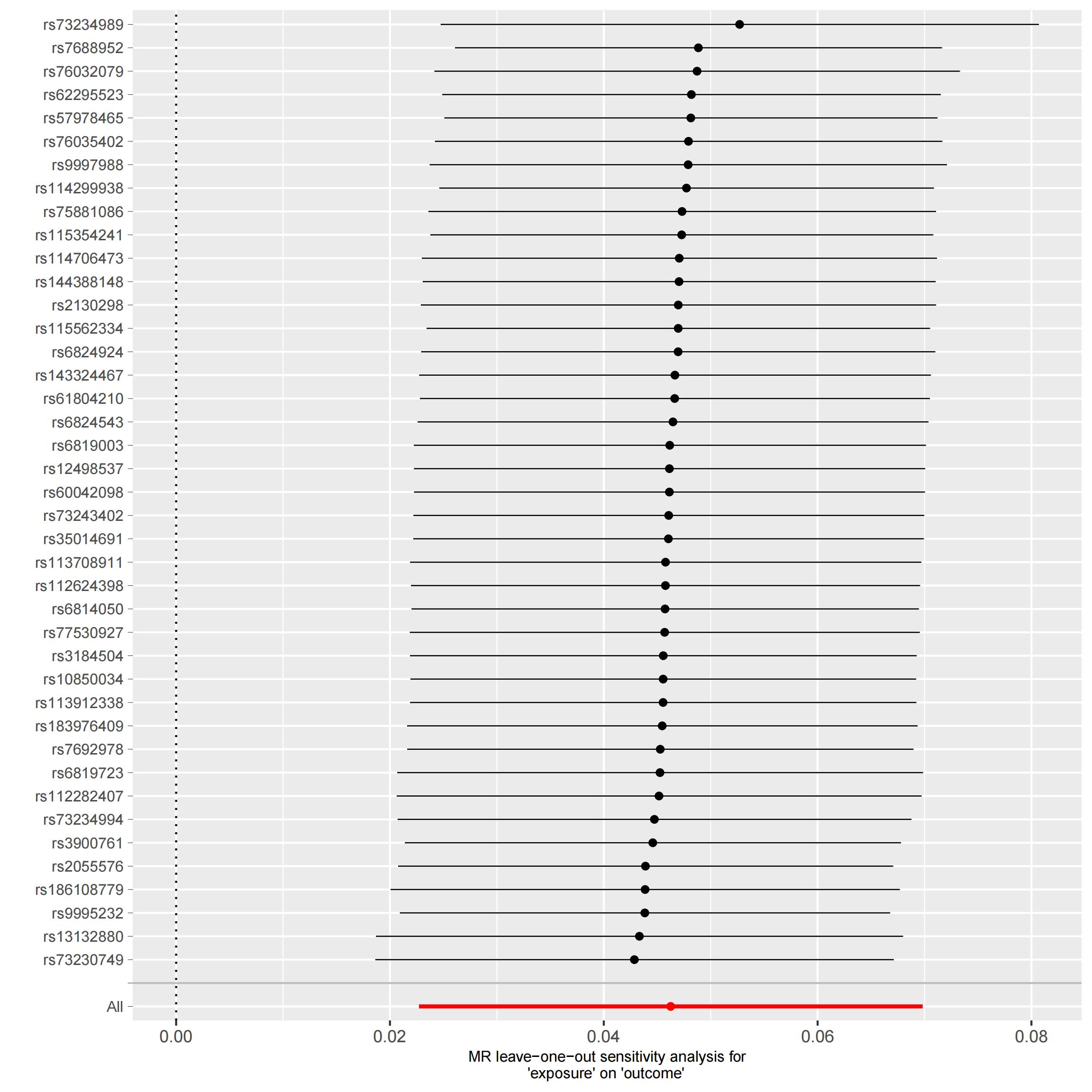  d |

Supplementary Figure S14 Forest plot (a), scatter plot(b), funnel plot (c) and sensitivity analysis (d) of SNPs associated with TLR1 on FURIN.
